# Lead Iodide Perovskite Thin Film Formation: The Impact of Preparation Method Studied by In Situ GIWAXS

**DOI:** 10.1021/acsami.5c18099

**Published:** 2025-12-03

**Authors:** Niels Scheffczyk, Ekaterina Kneschaurek, Paul Zimmermann, Lena Merten, Manuel Herbst, Florian Bertram, Ivan Zaluzhnyy, Alexander Hinderhofer, Frank Schreiber

**Affiliations:** † Institute of Applied Physics, University of Tübingen, Auf der Morgenstelle 10, 72076 Tübingen, Germany; ‡ 28332 Deutsches Elektronen-Synchrotron DESY, 22607 Hamburg, Germany

**Keywords:** perovskite, thin films, in situ GIWAXS, crystallization, spin-coating, antisolvent, gas-quenching

## Abstract

Lead halide perovskite materials have been successfully incorporated as the active layer into novel solar cells, the performance of which depends strongly on the structure and morphology of the perovskite thin film. This applies in particular to perovskites with a mixture of methylammonium (MA^+^) and formamidinium (FA^+^) as the A-site cation. Here, we present a thorough analysis of different mixed cation lead iodide perovskite crystallization scenarios using in situ grazing incidence wide-angle X-ray scattering (GIWAXS). We quantify the phase composition, crystallinity and orientational order of perovskite thin films for various preparation methods and the corresponding intermediate precursor phases. Specifically, we investigate one-step conversion (OSC), gas-quenching with nitrogen and antisolvent induced crystallization with three different antisolvents (chlorobenzene (CB), isopropanol (IPA), ethanol (EtOH)). We find that the average grain size is determined already during the formation of the intermediate phases and therefore it strongly depends on the preparation method. The alcoholic antisolvents introduce a more complex crystallization pathway, including new intermediate structures, and a preferred orientation, which is not necessarily retained by the perovskite thin film.

## Graphical abstract

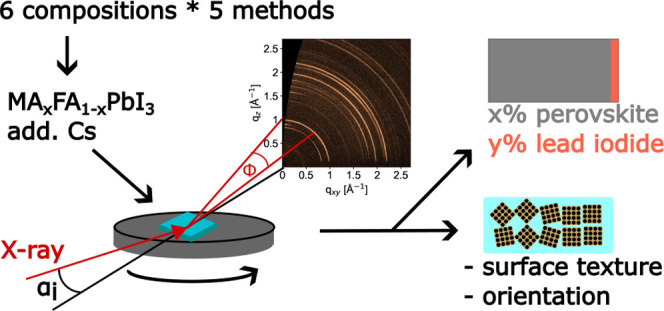

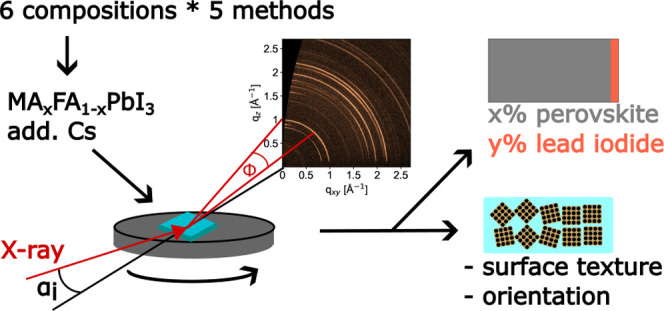

## Introduction

The global energy consumption keeps increasing every year and the demand for renewable energy sources continues to grow.^1^ Historically, the solar cell market and research is dominated by silicon solar cells,^2^ but in the past decade new thin film-based technologies emerged with the promise of lower production cost while achieving comparable efficiencies.^3,4^ One of the candidate materials used for these alternative solar cells are hybrid perovskites.^5^ They follow the general structure of ABX_3_, where A is a monovalent cation (often: methylammonium (MA^+^), formamidinium (FA^+^) or cesium (Cs^+^)), B is a bivalent cation (Pb^2+^ or Sn^2+^) and X is a halide anion (I^–^, Br^–^ or Cl^–^). The power conversion efficiency (PCE) of perovskite solar cells (PSCs) has increased from 3.8% to over 26% in only 15 years of research,^6,7^ meaning they can already compete with silicon-based solar cells regarding the PCE.

The first functional PSC contained MAPbI_3_ in the active layer, which is characterized by a small band gap (∼1.57 eV),^8^ enabling visible light absorption.^6^ MAPbI_3_ can form the perovskite structure at room temperature, which is beneficial for the cost and energy-efficient production of MAPbI_3_-based solar cells, but it decomposes into volatile organic compounds and PbI_2_ at elevated temperatures.^9,10^ The FAPbI_3_ composition with a different organic A-site cation features an even smaller band gap (∼1.48 eV)^8^ and is thermally more stable.^11^

However, at room temperature FAPbI_3_ mostly forms the yellow, photoinactive δ-phase.^12^ One way to facilitate the formation of the desired perovskite phase is to use a mixture of MA and FA cations, sometimes doped with inorganic cations, such as cesium and rubidium.^13,14^ Also, the incorporation of bromide has been shown to accelerate the formation of the perovskite phase, but it also introduces effects of halide segregation when used in combination with iodide, which are not yet entirely understood.^15–17^ Mixing different halide anions can be used to tune the band gap and increase the stability of the photoactive perovskite phase, with iodide and bromide being the most common combination.^18^

One of the biggest advantages of PSCs is the possibility of cost-efficient and scalable production of perovskite thin films via various solution-based techniques.^19^ The most common technique is a one-step method where the thin films are produced from the precursor solution by various deposition techniques, such as spin-coating^20^ and blade-coating.^21^ The analysis of thin films with X-rays is well established^22–24^ and a number of in situ studies during the annealing or spin-coating with subsequent annealing of the perovskite thin films revealed complex crystallization mechanisms.^25–28^ For most perovskite compositions, the formed intermediate phases contain solvent molecules.^29^ Thermal annealing is then needed to convert these intermediate phases to the perovskite phase.^30^ The one-step deposition method can be complemented by using different quenching methods, such as antisolvent-assisted or gas-quenching,^31,32^ to induce a supersaturation in the wet thin film and thus modify the properties of the resulting perovskite thin films such as crystallinity, surface morphology and the presence of defects, all influencing the electronic properties of the material and thereby the potential device efficiency and stability.^33,34^

In this study, we produce mixed cation lead iodide perovskite thin films to focus on the influence of the A-site cation and the fabrication method on the perovskite phase. Since the addition of bromide has been shown to influence the orientational order,^35^ the anion was chosen to be pure iodide. We are using our own, custom-built spin-coater,^36^ suitable for in situ GIWAXS experiments at synchrotron sources, and investigate the dependence of properties of the perovskite thin films, such as composition, average grain size and orientation, on the properties of the corresponding intermediate phases. This work provides comprehensive insights into these combinations of perovskite composition and fabrication methods with controlled experimental conditions.

## Results and Discussion

Perovskite thin films with different cation compositions with the general chemical formula (MA_*x*_FA_1–*x*_)­PbI_3_ (*x* = 0, 0.17, 0.5, 0.83, 1) and (MA_0.17_FA_0.83_)_0.95_Cs_0.05_PbI_3_ were prepared using spin-coating with three different protocols: the one-step conversion (OSC), the antisolvent-quenching^37^ and the gas-quenching technique.^32^ This specific triple cation composition was included in this study because it is a prominent candidate for perovskite solar cells (PSCs).^8^ For the gas-quenching route, we utilized dry nitrogen, and for the antisolvent routes we used chlorobenzene, a commonly used aromatic antisolvent,^38^ and two alcoholic antisolvents, namely ethanol and isopropanol, as they were observed to introduce a deviating crystallization behavior in an earlier work.^39^ The characterization of the final thin film and the observation of its formation via intermediate phases was performed using GIWAXS.^24,40–42^ To achieve a subsecond temporal resolution, the experiments were performed at the beamline P08 of PETRA III (DESY, Hamburg, Germany).^43^ In the following sections, we first demonstrate how the analysis of the GIWAXS data was done, followed by comparing the phase composition of the different samples. Then we investigate the surface texture in these samples and consequently analyze if the preferred orientation of the perovskite phase is inherited from the precursor phases.

Figure 1 shows GIWAXS patterns taken from the in situ measurements at different times during the perovskite thin film formation of the MA_0.17_FA_0.83_PbI_3_ composition, assisted by ethanol as an antisolvent. Diffraction peaks of different structures are occurring during the experiment: perovskite (gray circles), hexagonal FAPbI_3_ δ-phase (blue squares), (MA)_2_Pb_3_I_8_·2DMSO (cyan triangles), lead iodide (red triangles), a templating structure with unidentified composition (magenta diamonds) and the indium tin oxide (ITO) coating of the substrate (lime line). After the spin-coating started (Figure 1a) only the signal from the ITO coating of the substrate is visible. The dropped antisolvent (Figure 1b) induces multiple crystalline phases to form, starting with a short-lived (∼2 s) templating structure, also observed in our earlier work.^39^ Between spin-coating and annealing (Figure 1c), a second precursor phase, (MA)_2_Pb_3_I_8_·2DMSO, is forming. During the annealing (Figure 1d,e), the (MA)_2_Pb_3_I_8_·2DMSO phase rapidly converts into perovskite, while the δ-phase transforms at a lower rate. After annealing for 4 min (Figure 1f), all precursor phases are fully converted and some lead iodide formed alongside the perovskite structure.

**Figure 1:**
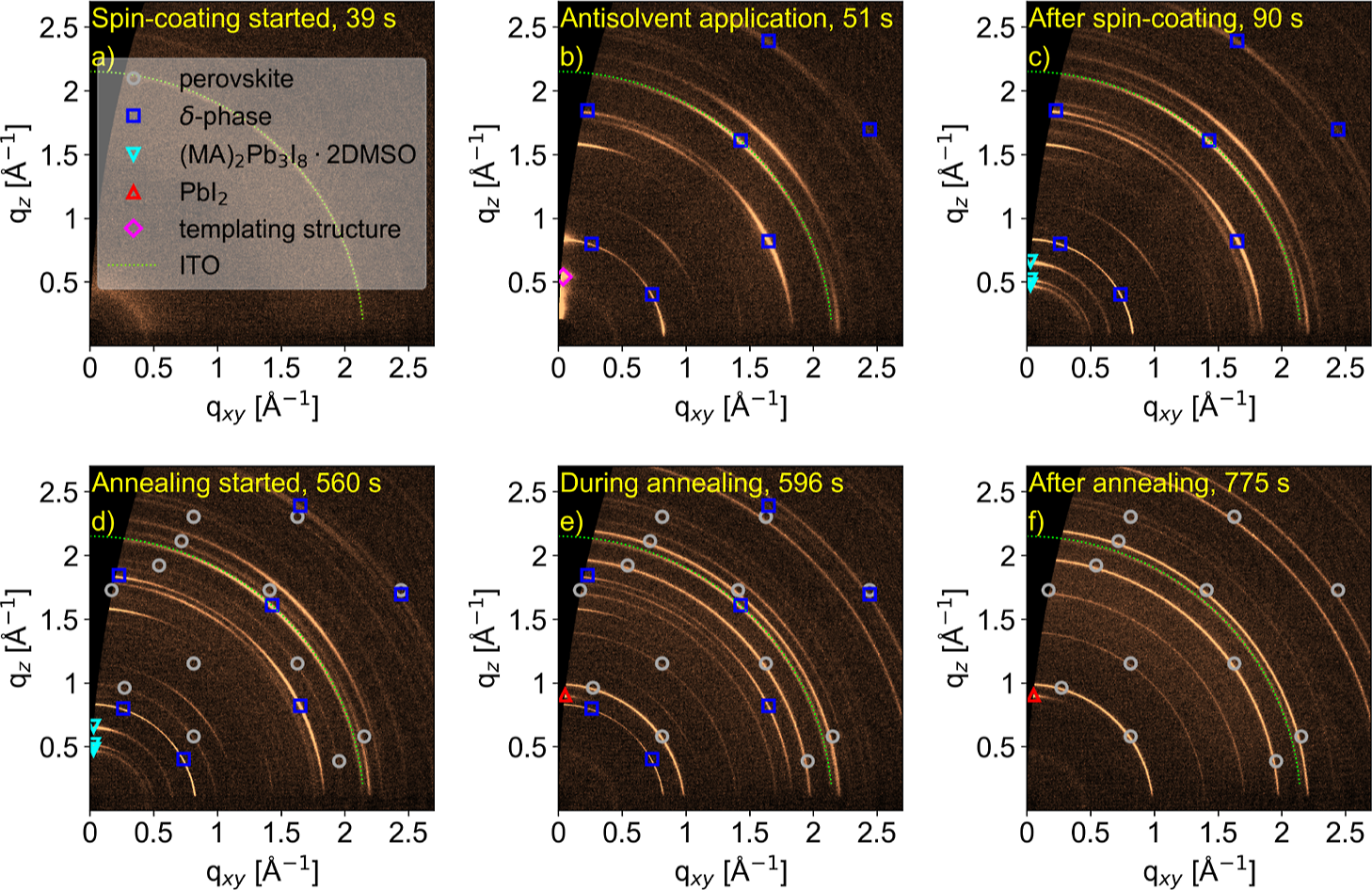
GIWAXS data during perovskite thin film formation with the composition MA_0.17_FA_0.83_PbI_3_ using ethanol as an antisolvent. Structures present at different times during the perovskite crystallization are perovskite (gray circles), FAPbI_3_ δ-phase (blue squares), (MA)_2_Pb_3_I_8_·2DMSO (cyan triangles), lead iodide (red triangles), a templating structure (magenta diamonds) and the ITO coating of the substrate (lime line). The GIWAXS patterns are taken before spin-coating started (a), at the time of the antisolvent application (b), between spin-coating and annealing (c), after the annealing started (d), at a later time during annealing (e) and after the annealing process (f).

To gain this knowledge, extensive analysis of the raw GIWAXS data is necessary. Figure 2 exemplarily shows the analysis carried out on all thin films investigated in this work. The first step in processing the GIWAXS real time data is generating the azimuthally integrated radial intensity profiles and comparing them to simulated XRD patterns of the known crystal structures, as shown in Figure 2a, to identify all crystalline phases observed during the thin film fabrication. The azimuthal profiles of the diffraction rings corresponding to the perovskite phase were then used to characterize both the surface texture and thereby the crystal grain size and the orientational order if present, as shown in Figure 2b. To gain insights into the crystallization pathway, the intensity evolution for all observed crystal phases during the perovskite crystallization is tracked during both spin-coating (Figure 2c) and annealing (Figure 2d) fabrication steps. The temperature during the annealing process is also shown in Figure 2d as a brown line. The intensity evolution during sample preparation for all other compositions and fabrication methods can be found in the Supporting Information (Figures S1–S29).

**Figure 2:**
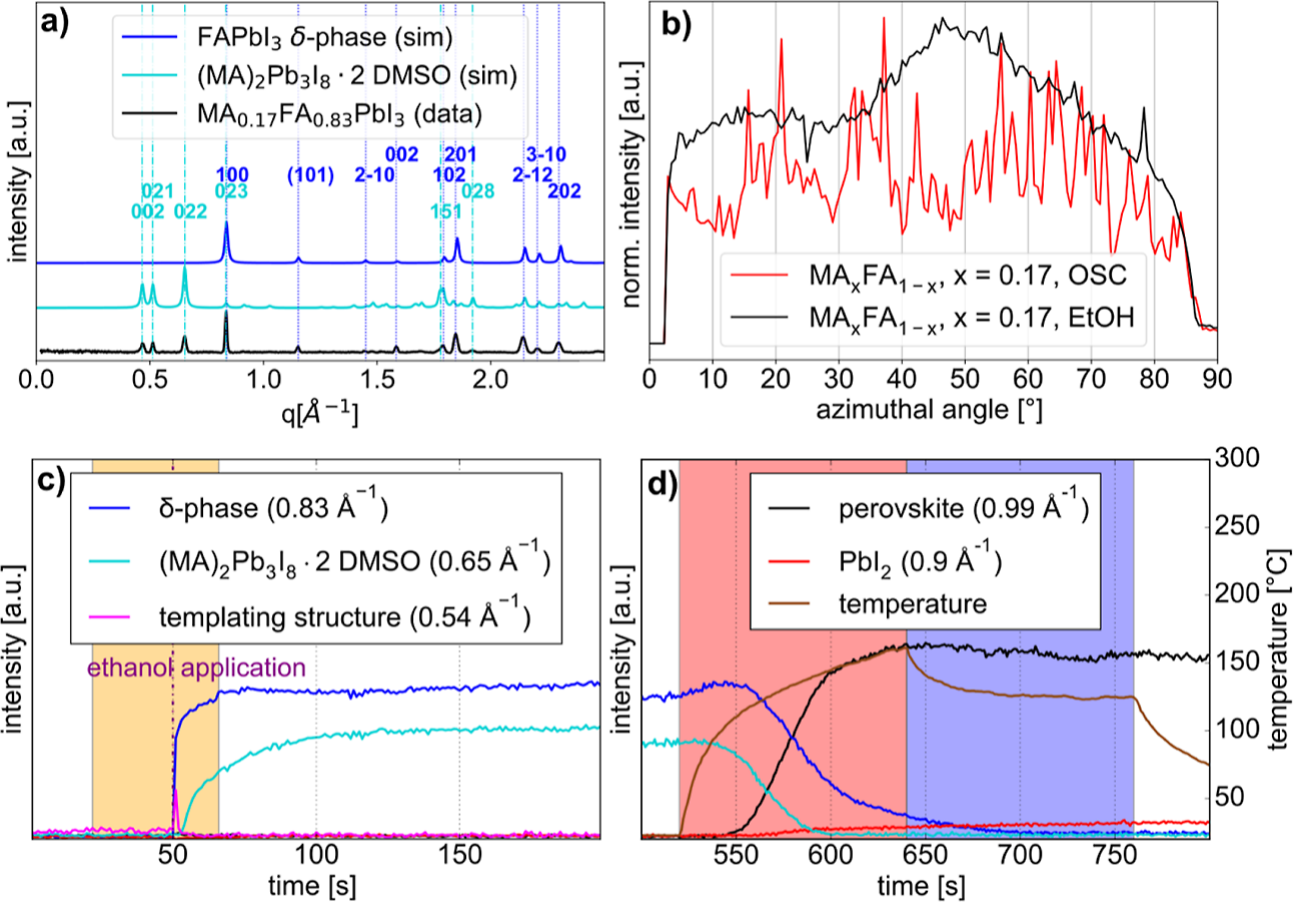
Step-by-step analysis of the MA_0.17_FA_0.83_PbI_3_ composition with ethanol applied as an antisolvent. (a) Experimentally measured radial intensity distribution (data) compared with simulated diffraction peaks (sim) from the known crystal structures.^44,45^ (b) Azimuthal profile of the 100 peak of the perovskite structure compared with the sample prepared via OSC. (c,d) Evolution of the intensity of selected diffraction peaks corresponding to the different crystal phases during the spin-coating and annealing of the sample, including the temperature during the annealing process. The chosen Bragg peak position is indicated in the legend. The yellow shaded region in (c) corresponds to the spinning time and the shaded regions in (d) correspond to the power of the halogen lamp used for IR annealing (red = 150 W and blue = 85 W).

### Crystal Structures in the Annealed Thin Films

For this study, perovskite films of six chemical compositions were prepared by five preparation methods, resulting in 6 × 5 = 30 combinations. Table 1 provides an overview over the relative amount of the crystalline phases present in the thin films after annealing: perovskite (gray in Table 1), lead iodide (red in Table 1) and the FAPbI_3_ δ-phase (blue in Table 1). Table S1 shows the percentages used for the visual representation. The determination of the relative amounts of these crystal phases was performed by comparing the integrated intensities of the selected characteristic peaks for each phase and structure factor calculations, see Experimental Details section for more details.^13^

**Table 1:**
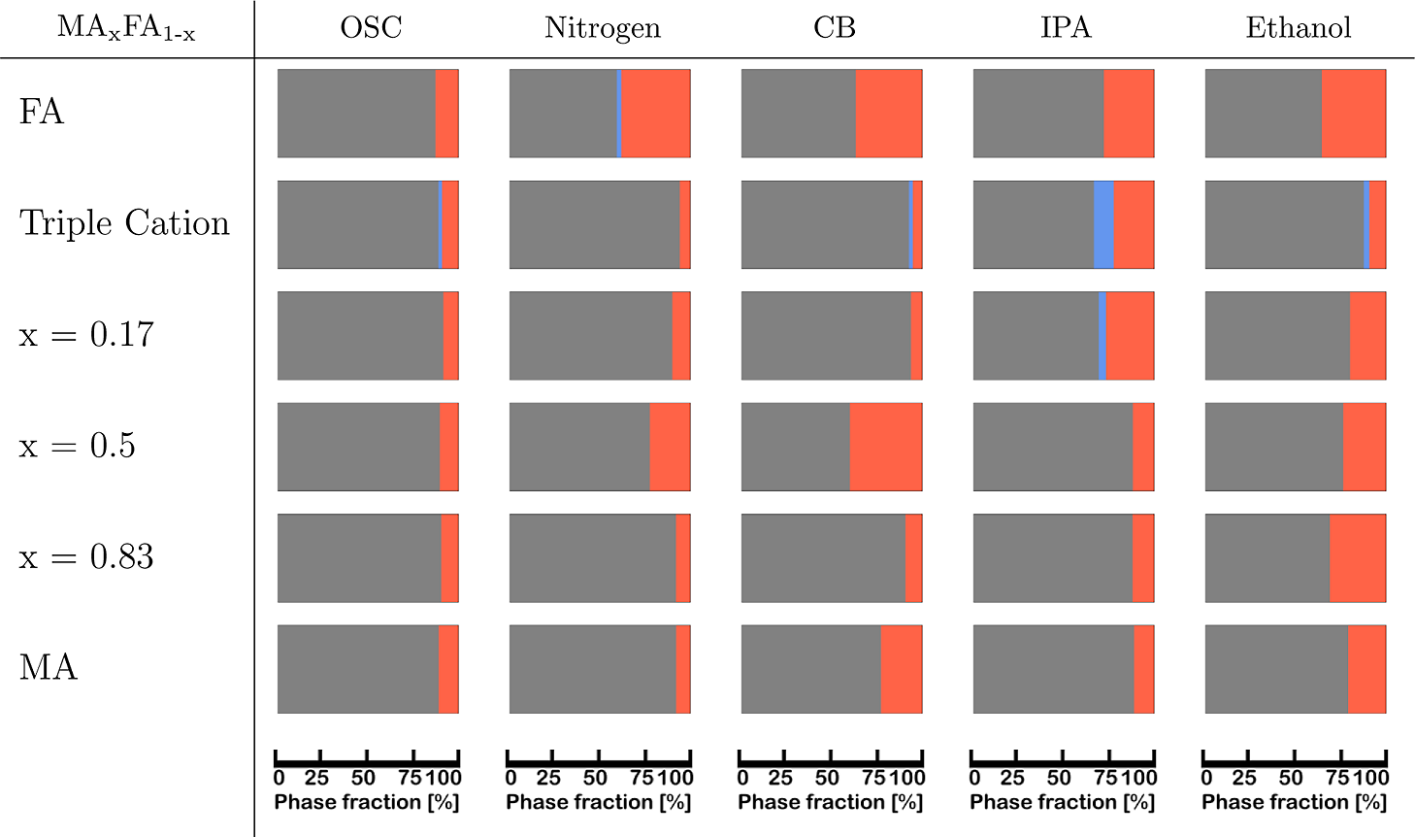
Crystal Structures Detected in the Annealed Thin Films, Presented as Molar Phase Fractions of Perovskite (Gray), the δ-Phase (Blue) and PbI_2_ (Red) in Percentages^a^

a The percentages used for the visual representation can be found in Table S1.

All investigated films contain the desired perovskite phase and a smaller amount of lead iodide after annealing. All compositions dominated by MA (>50%) fully converted their precursor phases and the thin films consist of only small amounts (<13%) of lead iodide. Only the pure MAPbI_3_ composition, fabricated with the CB antisolvent-assisted deposition technique, and both films fabricated with the ethanol antisolvent-assisted deposition technique contained a higher percentage of PbI_2_.

For the 1:1 composition, all precursor phases were fully converted to the perovskite phase as well. The lead iodide content is especially high for the antisolvent route using CB (40%), followed by ethanol (24%) and the gas-quenching technique (22%).

The FA-dominated (>50%) compositions require prolonged annealing, due to the higher conversion temperature of FAPbI_3_ compared to MAPbI_3_.^46,47^ The annealing setup with the IR heating lamp targeting the sample under an angle,^36^ due to geometric constraints within the spin-coating chamber, can lead to locally overheating the sample during the annealing procedure. This all results in some of the films not fully converting into the perovskite phase and a higher average presence of PbI_2_, compared to the corresponding MA-dominated compositions, as a result of degradation.

When 5% Cs^+^ is added to the cation mixture, the molar fraction of the perovskite phase increases to around 90% (except for the IPA antisolvent-assisted route, where the δ-phase and lead iodide coexist). Although all samples but the one prepared by the gas-quenching route still show residues of the δ-phase, the amount of non-perovskite phases is the smallest compared to the other FA-dominated mixtures. The assistance of cesium in the conversion of perovskite thin films with the composition of MA_0.17_FA_0.83_PbI_3_ is consistent with literature showing that the incorporation of Cs^+^ into the cation mixture is beneficial for the stability of mixed perovskite films and solar cells.^46,48^

Looking further into the differences between the fabrication methods, the antisolvent-assisted approach often results in a higher lead iodide content within the thin films, which is usually not desired, but can potentially be beneficial for the stability and performance of perovskite solar cells.^14,49^ Depending on the solubility of the different precursor components in the antisolvents,^50^ they wash out not only the solvent, but also some precursor components. Ethanol specifically possesses a non-negligible solubility for perovskite materials and has even been used as a solvent for perovskite precursor solutions with the addition of a Lewis base.^51,52^

In general, the rapidly introduced supersaturation of the precursor solution by either the gas-quenching or the antisolvent-assisted method works well for MA-dominated compositions. The formation of the (MA)_2_Pb_3_I_8_·2DMSO intermediate phase is energetically beneficial^53^ and the edge-sharing octahedra^54^ can act as a framework for the phase transformation, where at the appropriate annealing parameters DMSO, MA^+^ and I^–^ diffusion lead to an enhanced conversion.^55^ The formation energy of MAPbI_3_ is also negative (−0.95 eV^56^), leading to a stable perovskite composition.

For FA-dominated compositions, the formation of the δ-phase, featuring face-sharing octahedra, is energetically preferred in comparison to forming the FAPbI_3_ perovskite phase with the same chemical composition.^57–59^ The conversion of this crystal structure to the desired perovskite phase is energetically more expensive with a positive formation energy of 0.17 eV.^57,60^ This results in a longer and more complex annealing procedure at higher temperatures.

The increased PbI_2_ contents in thin films prepared with the antisolvent-assisted route could potentially be circumvented by adjusting the stoichiometry in the precursor solution, factoring in the increased solubility of the organic cations in the antisolvents.

### Crystallization Pathways

While the final phase composition of the annealed perovskite thin films could be determined by analyzing ex situ GIWAXS patterns, the in situ GIWAXS data provide unique insights into the crystallization pathways of the studied compositions and fabrication methods. For all combinations of preparation method and composition, data were acquired during both spin-coating and annealing fabrication steps. Figure 3 shows pseudo-2D XRD data, extracted from the in situ GIWAXS data by azimuthal integration, during spin-coating (left) and annealing (right) for the MA_0.17_FA_0.83_PbI_3_ composition using ethanol as an antisolvent. For the other 29 samples this data is shown in the Supporting Information, Figures S30–S58.

**Figure 3:**
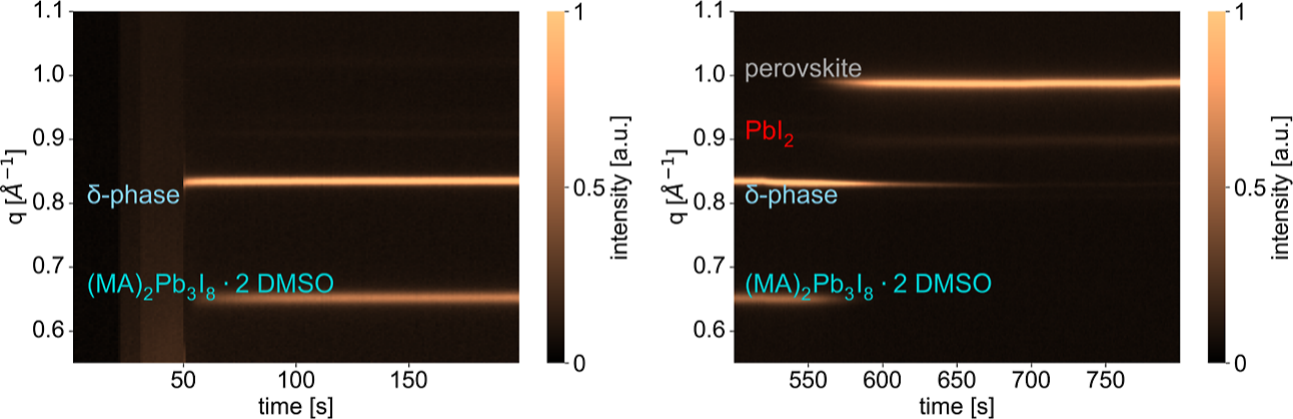
Pseudo-2D XRD data in a selected *q*-range showing the signals of interest, extracted from the in situ GIWAXS data during the spin-coating (left) and annealing (right) to follow the crystallization of the MA_0.17_FA_0.83_PbI_3_ composition using ethanol as an antisolvent. The crystal phases corresponding to the peaks visible are stated on the left side of the figures.

Table 2 shows the occurrence of precursor phases during the crystallization. Depending on the organic cation mixture, there are two different main precursor phases: the FAPbI_3_ δ-phase (blue in Table 2, occurring for FA-based compositions) and a DMSO-based solvent complex (MA)_2_Pb_3_I_8_·2DMSO (cyan in Table 2, occurring for MA-based compositions). For some compositions, multiple precursor phases can be identified, making the conversion into the perovskite phase more complex. In some samples, a small amount of the perovskite phase was formed after the respective quenching method was started, these are indicated by a gray star. The amount of perovskite phase was very low for all samples where it occurred before annealing and no clear trend for this behavior can be seen, other than the majority being fabricated with the antisolvent-assisted quenching method using ethanol.

**Table 2:**
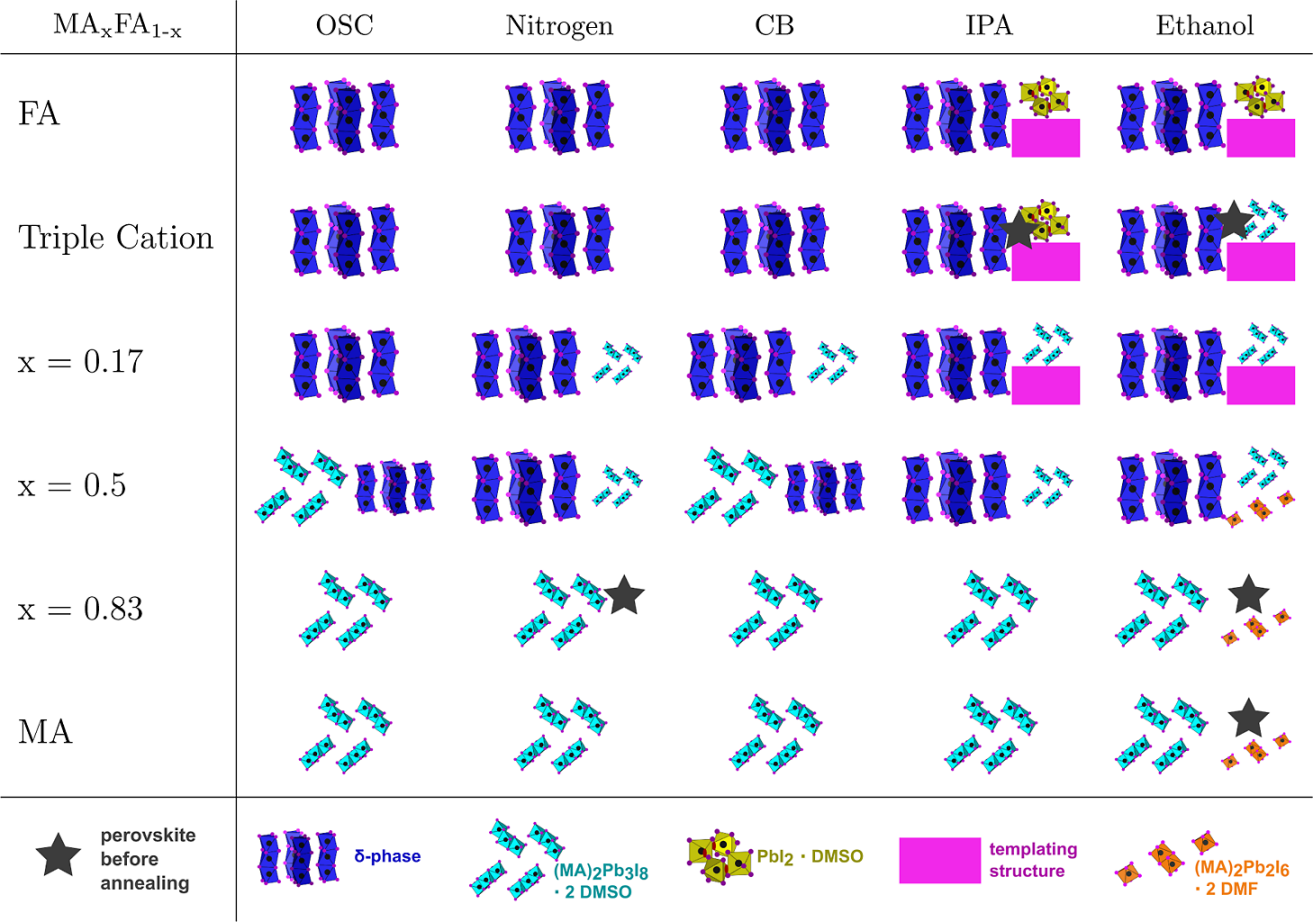
Crystalline Precursor Phases during Crystallization: The FAPbI_3_ δ-Phase (Blue), (MA)_2_Pb_3_I_8_·2DMSO (Cyan), the Templating Phase with an Unknown Crystal Structure^39^ (FAI—PbI_2_·*x*DMSO, Magenta), a DMF-Based Solvent Complex ((MA)_2_Pb_2_I_6_·2DMF—Orange) and a DMSO-Based Lead Iodide Complex (PbI_2_·DMSO—Yellow)^a^

a If more than one precursor phase is present throughout the crystallization process, the phase depicted in the left of a given column is the main precursor phase, while additional crystalline phases are shown smaller on the right side of the column. In some samples, a small amount of the perovskite phase was formed after the respective quenching method was started, these are indicated by a gray star.

The perovskite composition based on purely MA as the central cation shows a common crystallization pathway through the main precursor phase (MA)_2_Pb_3_I_8_·2DMSO (cyan in Table 2). Only ethanol as the quenching agent in the antisolvent-assisted fabrication route leads to an additional DMF-based solvent complex (orange in Table 2). When a relatively small amount of FA (16.6%) is added to the composition, the crystalline intermediate phases observed during crystallization are exactly the same as for the MAPbI_3_ composition. Further increasing and equalizing the amount of FA in the cation mixture (*x* = 0.5) leads to the formation of the FA-based δ-phase (blue in Table 2) as well in all samples. When FA is the dominating organic cation, the main precursor phase changes to the δ-phase for all fabrication methods and the (MA)_2_Pb_3_I_8_·2DMSO intermediate is found in smaller amounts during crystallization.

Comparing the influence of the different fabrication methods, for OSC there is a clear distinction in the exact time these two precursor phases are occurring on the path toward the perovskite phase for all compositions. The δ-phase forms quickly once the annealing process is started and needs the additional energy provided by the IR-lamp with the exception of the triple cation composition (compare Figures S1–S4), while the (MA)_2_Pb_3_I_8_·2DMSO intermediate develops gradually before annealing during the time after spin-coating when the precursor solvent evaporates at room temperature (compare Figures S4–S6). This highlights the necessity to control the timing between spinning and annealing as, depending on the composition and fabrication method, it can strongly influence the crystallization.

Employing any of the investigated quenching methods on compositions with ≥50% FA in the cation mixture leads to the δ-phase forming almost immediately after the quenching starts, while the MA-based precursor phase forms shortly after. Regarding the crystallization pathway, the gas-quenching route and CB in the antisolvent route show very similar results with only the main precursor phases present during crystallization.

The crystallization pathway becomes more complex if alcoholic antisolvents (IPA and ethanol) are used as quenching agents. A total of three additional precursor phases can be observed in the real-time data: PbI_2_·DMSO (yellow in Table 2), the templating structure observed in earlier work, which has been suggested to consist of FAI, PbI_2_ and DMSO,^39^ (magenta in Table 2) and (MA)_2_Pb_2_I_6_·2DMF (orange in Table 2). The short-lived (∼2 s) templating structure occurs for all compositions with more FA than MA as soon as the alcoholic antisolvent is dropped onto the sample. Furthermore, all compositions exhibit the PbI_2_·DMSO phase or the (MA)_2_Pb_3_I_8_·2DMSO precursor phase associated with the MA-based samples. The (MA)_2_Pb_2_I_6_·2DMF solvent complex occurs for compositions with at least 50% MA and ethanol used as the quenching agent in the antisolvent-assisted fabrication technique. While it is formed immediately, the intensity of the corresponding signal decreases to zero before the start of the annealing process as DMF evaporates faster than DMSO.^61^

### Surface Texture

Providing additional insight on the morphology of perovskite thin films, the azimuthal intensity distribution of the 100 diffraction ring of the perovskite phase was analyzed, as it was shown that the orientational order plays an important role in the performance of PSCs.^62^ During the calculation of the azimuthal profiles, geometric effects of ring distortion on a 2D detector were accounted for. Then, a background correction was applied to the azimuthal profiles. In order to disentangle intensity variations originating from a preferred crystallite orientation and large intensity fluctuations related to surface texture and grain distribution, all peaks along the azimuth (for |*q*| = 0.99 Å^–1^, representing the perovskite 100 diffraction signal), corresponding to a preferred crystallite orientation, were fitted by Gaussian functions and then subtracted from the intensity along the 100 ring. Generally, a spotty diffraction ring can be correlated to a thin film consisting of fewer grains compared to a thin film with a smooth diffraction ring.^63,64^

Figure 4 shows this procedure for two exemplary intensity curves and corresponding differences in the morphology of the thin films, visible even by eye. The azimuthal profile of the rough sample has large intensity variations and no orientational order, while the one of the smooth sample has smaller intensity variations and a preferred orientational order, as indicated by clear large scale maxima, fitted by the Gaussian curves in the azimuthal profile. For the remaining samples exhibiting a preferred orientation, the Gaussian fits can be found in the Supporting Information, Figures S59–S72. On the orientation-corrected azimuthal profile, the standard deviation of the signal was calculated to estimate the surface texture due to different grain sizes and grain counts. Table 3 shows all azimuthal profiles of the 100 perovskite diffraction ring for the studied compositions and deposition methods after the background correction has been applied. The bar chart on the background shows the standard deviation of the corresponding azimuthal profile, normalized to the standard deviations of the sample set. A higher standard deviation corresponds to a higher intensity fluctuation and thereby bigger and fewer crystal grains. Note that the *y*-axes of the different plots are not the same, resulting in some samples appearing smoother by eye than they are (e.g., triple cation composition prepared by OSC or CB as antisolvent).

**Figure 4:**
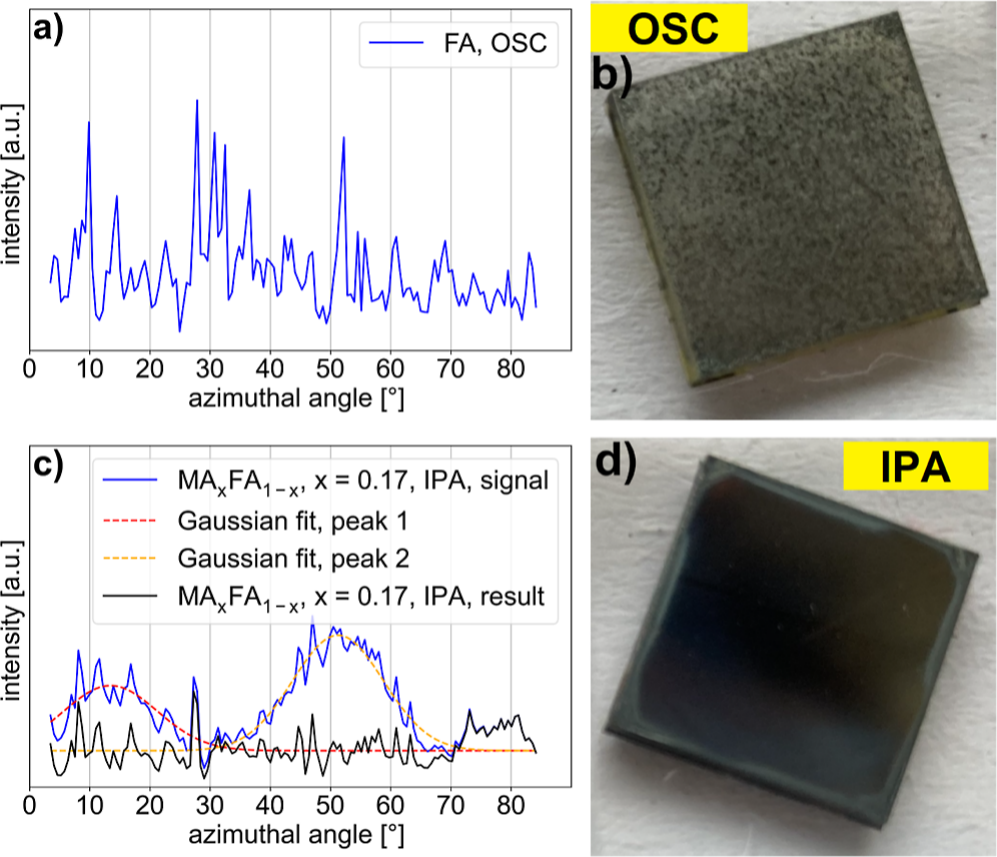
(a) Background-corrected azimuthal profile of the 100 peak of the perovskite structure for a sample prepared by OSC and (b) an image of the corresponding rough thin film. (c) Background-corrected azimuthal profile of the 100 peak of the perovskite structure for a sample prepared by using IPA as antisolvent (blue line), including Gaussian fits (red and orange dashed lines) of peaks in the azimuthal profile due to a preferred orientation and the resulting azimuthal profile (black line) after subtraction of the fitted peaks. (d) Image of a corresponding smooth thin film.

**Table 3:**
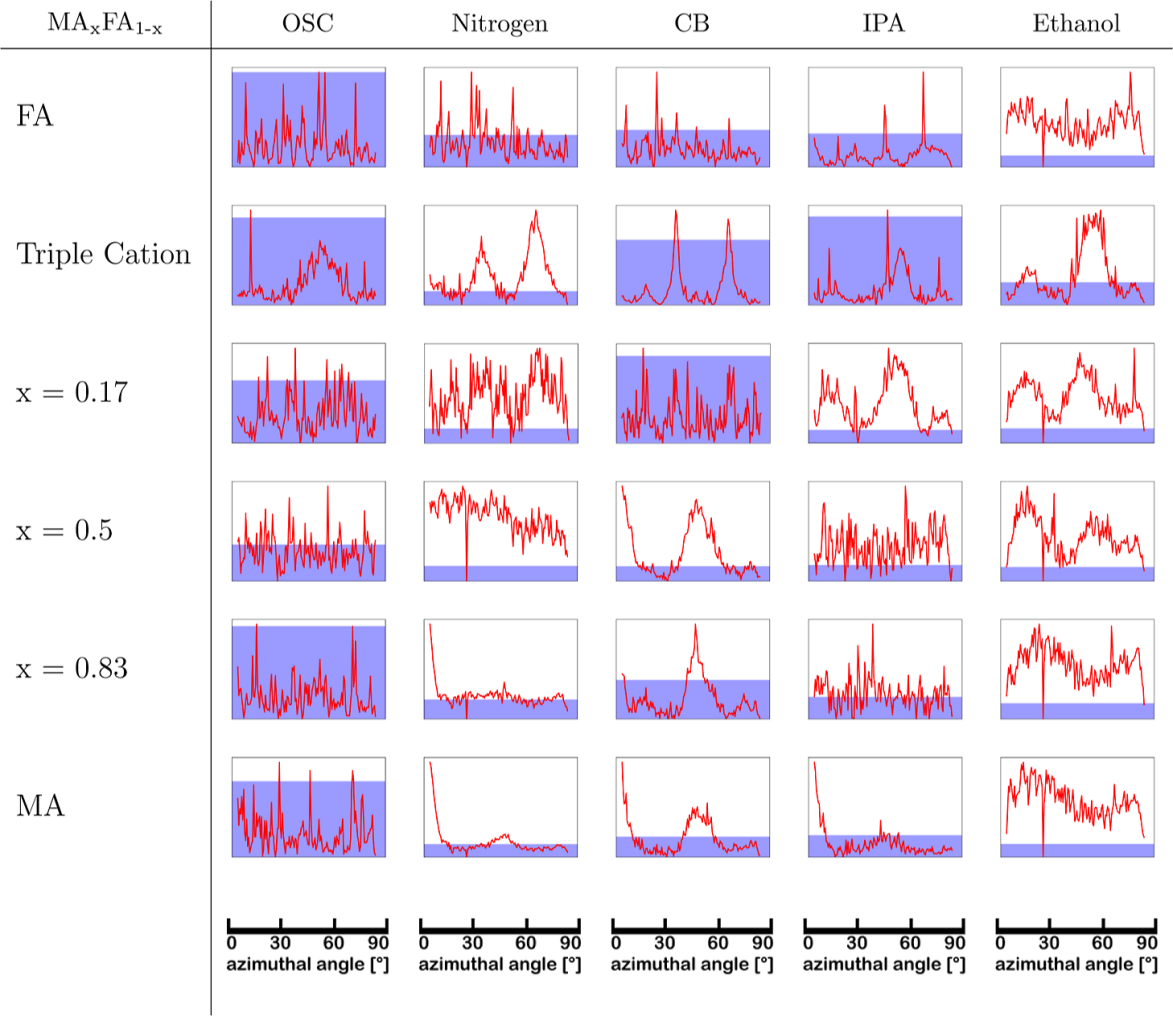
Surface Texture Derived from the 100 Perovskite Diffraction Ring by Calculating the Standard Deviation on the Background-Corrected Azimuthal Profiles after Peaks Originating from a Preferred Orientation Were Fitted by Gaussians and Subtracted from the Signal^a^

a The background-corrected azimuthal profiles after annealing *I*(ϕ) are plotted in red. The bar chart on the background shows the standard deviation of the corresponding azimuthal profile, normalized to the sample set. A higher standard deviation corresponds to a higher intensity fluctuation and thereby bigger and fewer crystal grains.

A few trends are visible in Table 3: using OSC as a fabrication route in general leads to thin films with few and large grains, resulting in rough film surfaces, which is consistent with the literature.^65^ Employing CB as an antisolvent also results in relatively large crystal grains, whereas gas-quenching results in very uniform azimuthal profiles (after considering preferred orientation). When comparing the surface texture among the studied compositions, we observe two of them (MA_*x*_FA_1–*x*_PbI_3_, *x* = 0.17 and *x* = 0.5) with a very low standard deviation of their azimuthal profiles in comparison to the other compositions indicating comparatively many small crystal grains.

While this comparison of the surface texture derived from the 100 diffraction ring of the perovskite structure confirms that using the OSC fabrication route results in rather rough thin films featuring few and large grains (see Figure 4), it also shows that different preferred orientations within the perovskite phase are introduced by the investigated fabrication methods. These features will be discussed in the next section.

### Orientation

From Table 3 it is already apparent that there are multiple preferred orientations present in the deposited thin films. It is important to note that the orientational order exhibited by all samples is weak and a significant amount of the perovskite crystallites is randomly oriented. Hence, the orientations investigated here are always present on top of a generally randomly oriented thin film. The results of this investigation are shown in Table 4. In general, the perovskite phase in the investigated samples has been observed in four different preferred orientations: azimuthal intensity maxima at 15° and 54° (green), maxima at 35° and 66° (red), maxima at 0° and 45° (cyan) and no maxima (yellow) in the azimuthal profile of the 100 diffraction ring. All azimuthal angles are measured relative to the surface normal, so 0° is along *q*_*z*_, which is not available in the GIWAXS measuring geometry due to the missing wedge. In this section 0° means that the intensity maximum is localized next to the missing wedge, i.e. as close to the *q*_*z*_ as observable in the data. The orientation resulting in azimuthal intensity maxima at 15° and 54°, meaning that the perovskite unit cells are oriented with their corners facing up ((111) plane parallel to the substrate), was also observed in earlier work^39^ with a triple cation lead halide perovskite, featuring 10% of bromide. In the case of the purely iodide-based composition, the degree of orientation is much lower, as a uniform Debye–Scherrer ring is observed, which sometimes shows intensity maxima alongside for all samples. This orientation (green) is adopted as well for the mixed compositions with more FA than MA, using alcoholic antisolvents. The triple cation composition features another preferred orientation (red) for gas-quenching and CB. For this orientation none of the accessible crystal planes are facing up, meaning that the perovskite unit cells are oriented at an angle for all (accessible) planes compared to the surface normal on top of the random distribution present in all samples. MA-dominated compositions often result in the (100) plane of the perovskite unit cell stacking parallel to the substrate (peak at 0°, cyan) or the (110) plane of the perovskite unit cell stacking parallel to the substrate (peak at 45°, cyan), especially for gas-quenching and antisolvent-quenching with CB. The rest of the samples, in particular all purely FA-based thin films, did not show any preferred orientation (yellow).

**Table 4:**
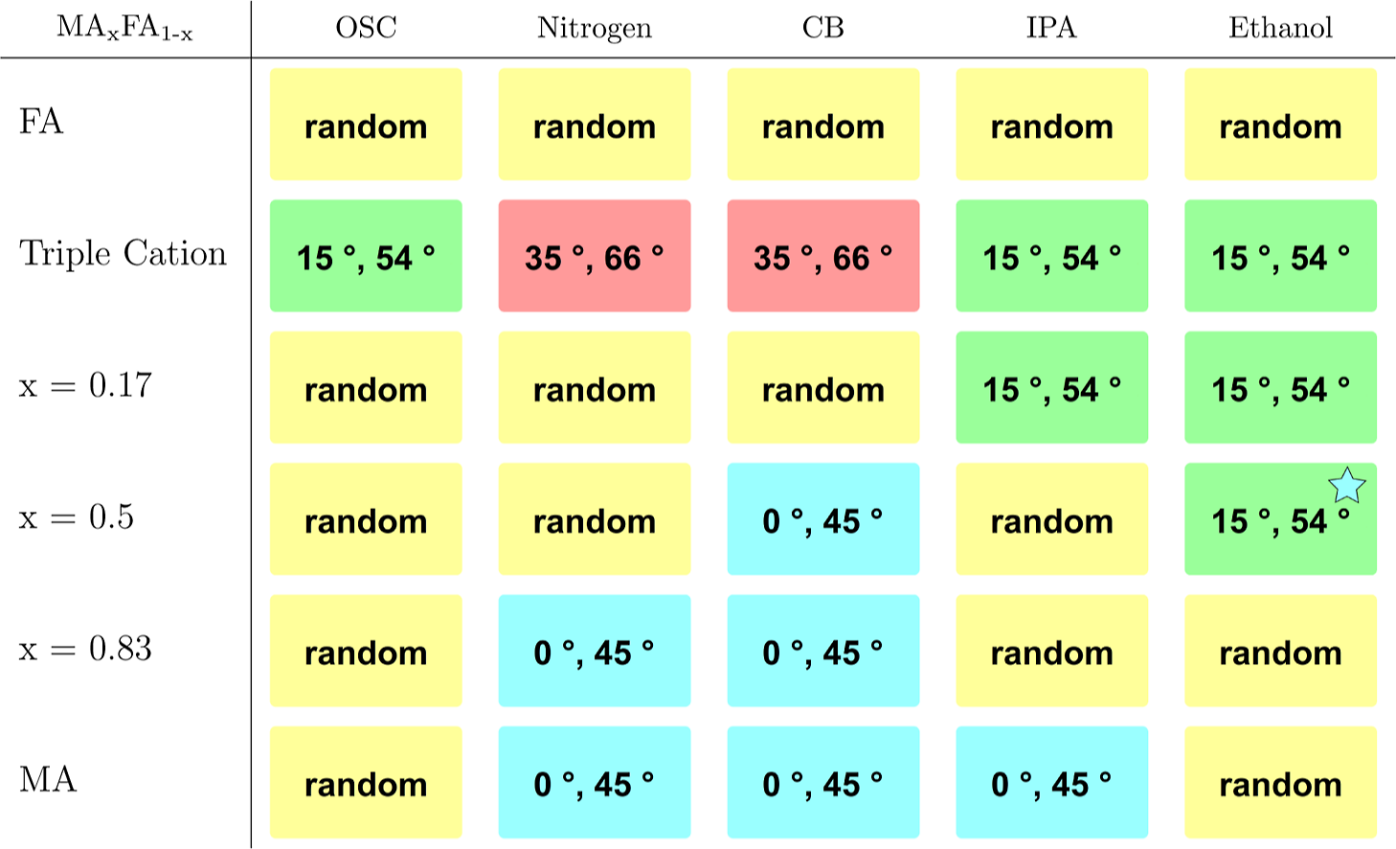
Orientation in the Perovskite Phase for All Compositions and Methods^a^

a There are four different preferred orientations: intensity maxima at 15° and 54° (green), maxima at 35° and 66° (red), maxima at 0° and 45° (cyan) and randomly distributed intensity in the Debye–Scherrer ring (yellow). For the green orientation marked with a cyan star the modulation around 54° features a broad peak shifted towards 45°, compare Figure S71.

Comparing the occurrence of the templating phase during the spinning process and the (111) orientation of the perovskite phase in the final thin film (green), they coincide very well. To further look into the influence of precursor phases and their orientation on the orientation of the perovskite phase, in Figure 5 we present some selected azimuthal profiles of the 100 diffraction signal of both the FAPbI_3_ δ-phase and the perovskite phase of the corresponding sample. All shown compositions are with a majority of FA, prepared by either the gas-quenching route or using CB as an antisolvent.

**Figure 5:**
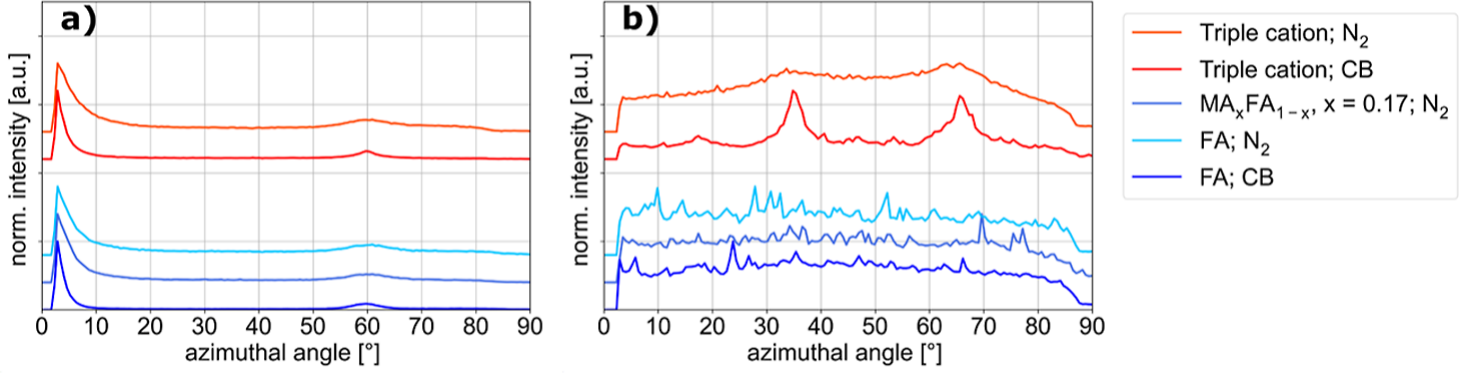
Azimuthal profile of the 100 diffraction signal of δ-phases before annealing (a) and of the 100 peak of their corresponding perovskite phases after annealing (b) in selected compositions. All compared compositions have *x* > 0.5 and are prepared by either the gas-quenching route or using CB as antisolvent. Curves are shifted for better visibility.

The azimuthal profiles suggest that for the samples with a shared orientation of the perovskite phase (red lines), their main precursor phases also share a preferred orientation. In Figure 5b, the azimuthal profiles of the perovskite phase (red curves) have maxima in intensity at 35° and 66°. The azimuthal profiles of the corresponding δ-phases (Figure 5a, red curves) show maxima in intensity at 0° and 60°. Extending the comparison to other samples with similar preparation parameters (composition and method) the azimuthal profiles of the δ-phase (Figure 5a, blue curves) look exactly like those mentioned before. In the corresponding annealed perovskite phases (Figure 5b, blue curves), no maxima in intensity can be seen. This indicates that the orientation suggested by the precursor phase is not always retained to the perovskite phase, but also no different orientation is adopted by the perovskite phase. To further elucidate this for all combinations of composition and fabrication method, Table 5 shows which preferred orientation we would expect the perovskite phase to have, based on the crystallization pathway, i.e. the occurrence of the templating phase, and the preferred orientation of the main intermediate phase. The azimuthal profiles of the 100 peak of the δ-phase and the 022 peak of the (MA)_2_Pb_3_I_8_·2DMSO phase for all applicable samples can be found in the Supporting Information (Figures S73–S79).

**Table 5:**
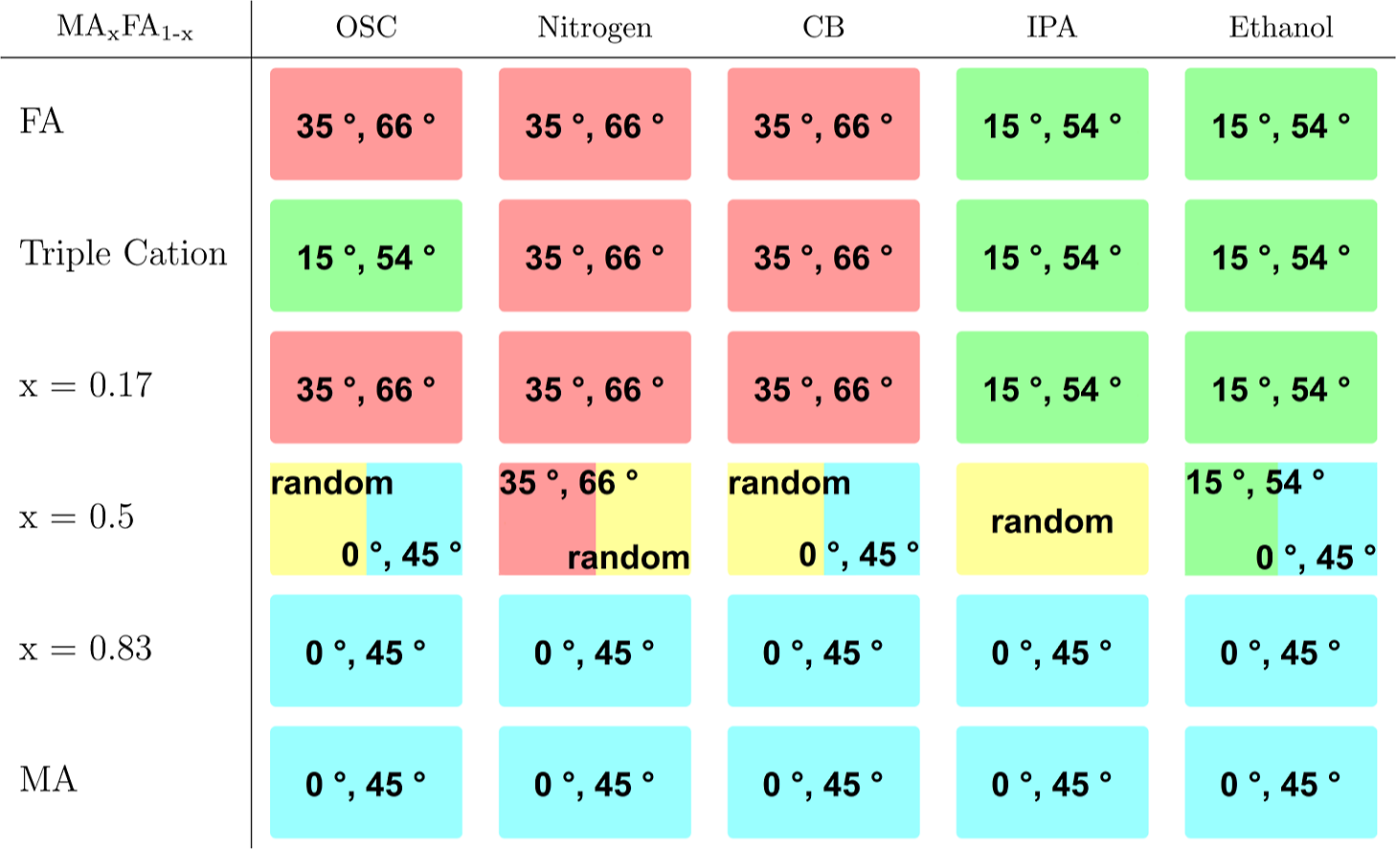
Expected Orientation of the Perovskite Phase Based on the Crystallization Pathway (i.e. Occurrence of the Templating Phase) and the Preferred Orientation of the Main Precursor Phases^a^

a The color code is the same as in Table 4. The angle pairs listed here do not refer to the precursor phases, but to the expected orientation of the perovskite phase. For the MA_*x*_FA_1–*x*_, *x* = 0.5 composition, both of the main precursor phases are present, so the left side of the column corresponds to the orientation the δ-phase suggests and the right side to the orientation suggested by the (MA)_2_Pb_3_I_8_·2DMSO phase.

The preferred orientations expected based on the precursor phases give a much clearer trend than the ones observed in the perovskite phases. All compositions with more MA than FA exhibit the same preferred orientation, which is retained to the perovskite phase mostly if the films are fabricated by either gas-quenching or antisolvent-quenching with CB. For the compositions with more FA than MA, the δ-phase shows two different preferred orientations, matching the occurrence of two different orientations in the perovskite films, depending on the preparation method. The alcoholic antisolvents, by facilitating the templating structure, give a different orientation than OSC, gas-quenching or CB as antisolvent fabrication routes. Notably, the triple cation composition is the only case in which the preferred orientation indicated by the δ-phase is consistent with the actual preferred orientations of the corresponding perovskite phase. This shows that the addition of Cs to the organic cation mix leads to a more reproducible and stable crystallization behavior, which is beneficial for the preparation of PSCs.

For the *x* = 0.5 mixture, the behavior regarding preferred orientations is slightly more complicated. All samples show both of the main precursor phases and usually at least one of them already shows no preferred orientation, which then results in a randomly oriented perovskite phase. For the sample prepared with EtOH as antisolvent, the orientation of the perovskite phase could feature the (110) orientation expected from the (MA)_2_Pb_3_I_8_·2DMSO intermediate in addition to the (111) orientation expected from the δ-phase, as the peak at around 54° is rather broad and shifted toward 45°.

In general, the results show that the orientation of the perovskite phase is predetermined by the orientation of the precursor phases, but often not retained through the annealing process. For pure FA-based samples, with the energetically expensive (positive formation energy) conversion from the δ-phase to the perovskite phase, no orientation is retained. This is in contrast to the MA-based compositions, where the (MA)_2_Pb_3_I_8_·2DMSO intermediate can act as a framework during the phase conversion.^55^ It is worth noting here that the influence of intermediate phases on the perovskite phase is limited due to the first order phase transformation, but they can provide local structural environments which can alter the growth of the perovskite crystallites.^39,55^ For the triple cation composition, the presence of Cs stabilizes the perovskite phase and the preferred orientation is retained for all fabrication methods investigated.^46,66^

## Conclusions

In summary, we investigated the formation of perovskite compositions with different cation mixtures and fabrication methods in this comprehensive study. The A-site cations strongly influence the crystal phases occurring during crystallization, where MA-based perovskite compositions exhibit a crystallization pathway via (MA)_2_Pb_3_I_8_·2DMSO and FA-based perovskite materials convert from the δ-phase to the perovskite phase. The latter conversion is energetically unfavorable and requires longer annealing at higher temperatures than MA-based perovskite compositions. For cation mixtures both of the pathways are occurring simultaneously until the annealing is combining all of the material to one perovskite phase. The gas-quenching route results in smooth, powdery films with smaller average grain sizes than the OSC route. Using alcoholic antisolvents as quenching agents during spinning leads to additional intermediate phases appearing, and also influences the preferred orientation of FA-based perovskite compositions. The orientational order of the precursor phase is frequently not retained in final perovskite phase, showcasing the high mobility of ions and complete grains during the annealing process. Interestingly, only for the triple cation composition the perovskite phase retained the orientation independent of the preparation method.

## Experimental Details

### Perovskite Precursor Solutions

The chemicals used to prepare the precursor solutions were: lead iodide (PbI_2_, purchased from Sigma-Aldrich with a purity of 99.999%), methylammonium iodide (MAI, purchased from Dyenamo with a purity of 99.99%), formamidinium iodide (FAI, purchased from Dyenamo with a purity of 99.99%) and cesium iodide (CsI, purchased from Dyenamo with a purity of 99.99%) The precursor solutions were prepared with a concentration of 1.4 M in a solvent mixture of dimethylformamide (DMF, purchased from Sigma-Aldrich with a purity of 99.9%) and dimethyl sulfoxide (DMSO, purchased from Sigma-Aldrich with a purity of 99.9%) in a ratio of 4 to 1. The solutions were stirred overnight at 80 °C in a nitrogen atmosphere and used within a few days of preparation. Before taking solution for experiments, the bottles were shaken to ensure a mixed solution.

### Thin Film Fabrication via OSC

The film fabrication was done in a custom-built sample environment,^36^ allowing in situ GIWAXS data acquisition. 60 μL of the precursor solution was deposited onto the 10 × 10 mm^2^ glass substrate with an ITO coating and then spun at 1000 rpm for 10 s followed by 4000 rpm for 30 s. Afterward, the samples were annealed for 4–10 min at 140 °C. GIWAXS data was recorded during the deposition process.

### Thin Film Fabrication via Antisolvent

The thin films were prepared similar to the OSC route. During the spinning process, the antisolvent was rapidly deposited onto the spinning sample 35 s into the procedure, inducing instant supersaturation within the thin film.^37,38^ Subsequently the thin films were also annealed for 4–10 min at 140 °C. GIWAXS data was recorded during the deposition process.

### Thin Film Fabrication via Gas-Quenching

The perovskite films were prepared in the same spin-coating chamber. 60 μL of precursor solution was put on 10 × 10 mm^2^ glass substrates with an ITO coating and then spun at 3000 rpm for 150 s. A nitrogen flow was directed at the center of the sample after 15 s of spinning to realize gas-quenching.^17,32^ The spin-coated thin films were then also annealed for 4–10 min at 140 °C. GIWAXS data was recorded during the deposition process.

### GIWAXS

All in situ GIWAXS experiments were done at the beamline P08,^43^ at DESY, Hamburg, Germany. The beam energy was set to 18 keV and the angle of incidence was 0.5°. The beam size was 100 μm (vertical) × 400 μm (horizontal). A 2D PerkinElmer detector (XRD 1621 CN3 EHS, 2048 × 2048 pixel) recorded diffraction patterns with a time resolution of 0.1 s at a distance of 750 mm to the sample. The beam energy of 18 keV was chosen to ensure a high *q*-range and a relatively flat Ewald-sphere to minimize the missing wedge area. Also, the area detector available at the time of the experiments is only efficient for the X-ray energy above 18 keV. For in situ experiments, the time of zero is defined by the beam shutter opening, note that the precursor solution is already on the substrate beforehand.

For the ex situ GIWAXS experiments, all parameters except the angle of incidence were the same as for the in situ experiments. The angle of incidence was varied between 0° and 0.3°, to gain more detailed information on the surface and the bulk, respectively.^67^ Prior to any analysis of the GIWAXS data, a polarization correction was applied during the data conversion to account for the predominantly horizontal polarization of the synchrotron X-ray beam.

### Phase Fraction Calculations

The peaks selected for the three different crystal structures were: 100 at |*q*| = 0.83 Å^–1^ for the δ-phase (multiplicity *m* = 2), 001 at |*q*| = 0.91 Å^–1^ for PbI_2_ (multiplicity *m* = 1) and 100 at |*q*| = 0.99 Å^–1^ for the perovskite phase (multiplicity *m* = 3). During the calculation of the structure factors, using the atomic positions in the crystal structures, the Lorentz correction was applied^68^

$$L\left(\theta \right)=\frac{1}{4\times \sin^{2}\theta \;\cos\theta }$$

The crystal structure factors *F*_*hkl*_ were calculated from the known atomic scattering form factors for the given crystal structures as^69^

$$F_{hkl}\left(Q\right)=\sum_{j}f_{j}\left(Q\right)e^{iQr_{j}}$$

where the atomic scattering form factors were estimated using the Cromer–Mann parametrization.^70^ Additionally, the multiplicity of the analyzed peaks was taken into account during the phase fraction calculations.^45,71^

## Supplementary Material



## Notes

Additional figures for intensity evolutions and azimuthal profiles (PDF)

## §

Division of Physical Chemistry, Lund University, Naturvetarvägen 14, 22100 Lund, Sweden
