# Supplementary material for: Lead Iodide Perovskite Thin Film Formation: The Impact of Preparation Method Studied by In Situ GIWAXS
Source: ACS Appl Mater Interfaces. 2025 Dec 3;17(50):67914–25. doi: 10.1021/acsami.5c18099 (PMC12723630; doi:10.1021/acsami.5c18099)
Supplement: Supplementary file 1 [file am5c18099_si_001.pdf]

Supporting Information

Lead Iodide Perovskite Thin Film Formation: The Impact of Preparation Method Studied by In-situ GIWAXS

Niels Scheffczyk,[†] Ekaterina Kneschaurek,[†] Paul Zimmermann,[†] Lena Merten,[†,¶] Manuel Herbst,[†] Florian Bertram,[‡] Ivan Zaluzhnyy,[†] Alexander Hinderhofer,[*,†] and Frank Schreiber[*,†]

†*Institute of Applied Physics, University of Tübingen, Auf der Morgenstelle 10, 72076 Tübingen, Germany*

‡*Deutsches Elektronen-Synchrotron DESY, 22607 Hamburg, Germany*

¶*Current adress: Division of Physical Chemistry, Lund University, Naturvetarvägen 14, 22100 Lund, Sweden*

E-mail: alexander.hinderhofer@uni-tuebingen.de; frank.schreiber@uni-tuebingen.de

Supporting Information

Figures S1 - S29 show the intensity evolution over time for all methods and compositions. Starting with the one-step-conversion (OSC), followed by the gas-quenching, finishing with the three antisolvent-assisted quenching routes (AS): chlorobenzene (CB), isopropanol (IPA) and ethanol (EtOH). Table S1 show the values of the phase fraction of all crystal phases detected in the finished thin films. Figures S30 - S58 show pseudo-2D XRD data for all

methods and compositions during spin-coating and annealing. Figures S59 - S72 show the azimuthal profiles of the 100 diffraction signal of the perovskite phase for the oriented samples with the gaussian fits to those peaks. Figures S73 - S79 show the azimuthal profiles of the main intermediate phases for all samples.

All information is extracted from grazing incidence wide-angle X-ray scattering (GIWAXS) data.

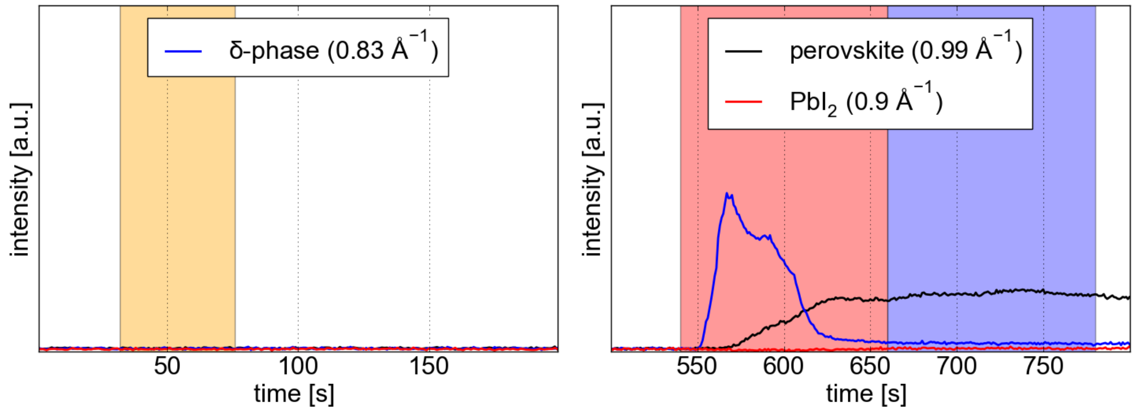

Figure S1: Method: OSC; Composition: $FAPbI_3$. Evolution of the intensity of selected diffraction peaks corresponding to the different phases during the spin-coating (left) and annealing (right) of the sample. The chosen peak position is indicated in the legend. The yellow shaded region in the left panel corresponds to the spinning time and the shaded regions in the right panel correspond to the power of the halogen lamp used for IR annealing (red = 150 W and blue = 85 W).

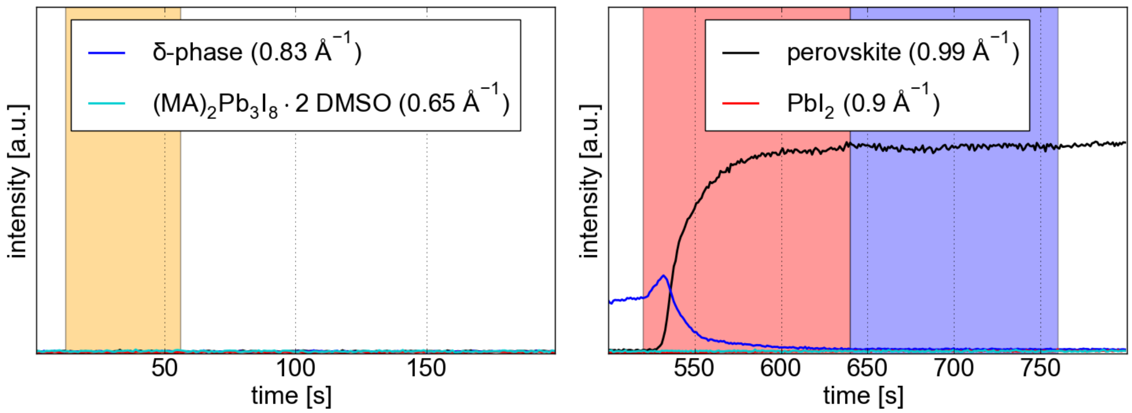

Figure S2: Method: OSC; Composition: $(MA_{0.17}FA_{0.83})_{0.95}Cs_{0.05}PbI_3$. Evolution of the intensity of selected diffraction peaks corresponding to the different phases during the spin-coating (left) and annealing (right) of the sample. The chosen peak position is indicated in the legend. The yellow shaded region in the left panel corresponds to the spinning time and the shaded regions in the right panel correspond to the power of the halogen lamp used for IR annealing (red = 150 W and blue = 85 W).

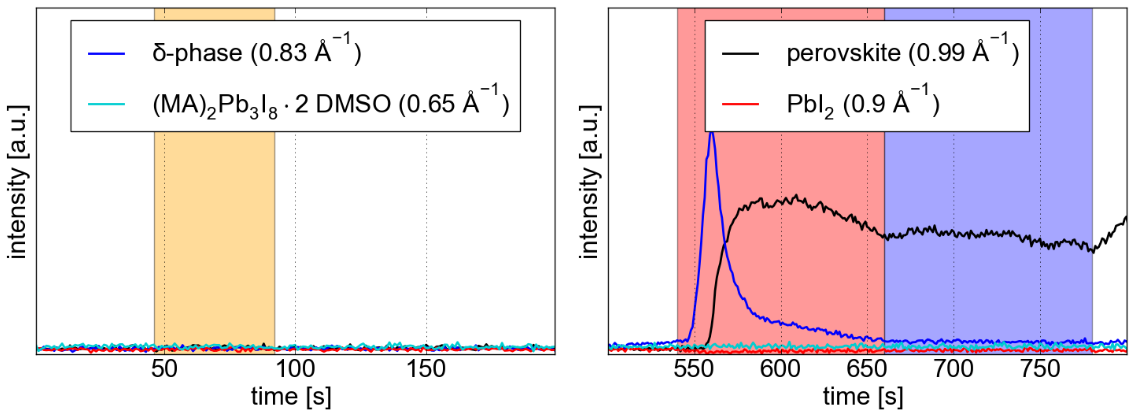

Figure S3: Method: OSC; Composition: $MA_{0.17}FA_{0.83}PbI_3$. Evolution of the intensity of selected diffraction peaks corresponding to the different phases during the spin-coating (left) and annealing (right) of the sample. The chosen peak position is indicated in the legend. The yellow shaded region in the left panel corresponds to the spinning time and the shaded regions in the right panel correspond to the power of the halogen lamp used for IR annealing (red = 150 W and blue = 85 W).

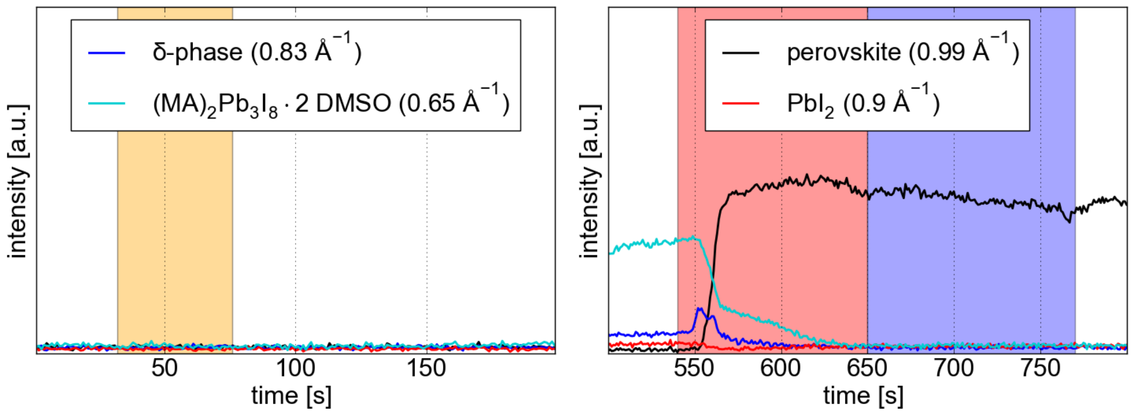

Figure S4: Method: OSC; Composition: $MA_{0.5}FA_{0.5}PbI_3$. Evolution of the intensity of selected diffraction peaks corresponding to the different phases during the spin-coating (left) and annealing (right) of the sample. The chosen peak position is indicated in the legend. The yellow shaded region in the left panel corresponds to the spinning time and the shaded regions in the right panel correspond to the power of the halogen lamp used for IR annealing (red = 150 W and blue = 85 W).

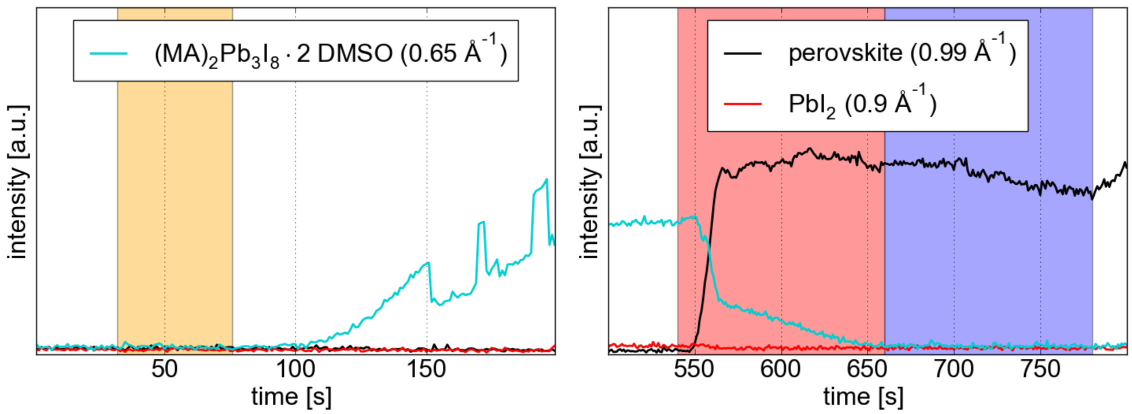

Figure S5: Method: OSC; Composition: $MA_{0.83}FA_{0.17}PbI_3$. Evolution of the intensity of selected diffraction peaks corresponding to the different phases during the spin-coating (left) and annealing (right) of the sample. The chosen peak position is indicated in the legend. The yellow shaded region in the left panel corresponds to the spinning time and the shaded regions in the right panel correspond to the power of the halogen lamp used for IR annealing (red = 150 W and blue = 85 W). Intensity jumps after 150 s in the left panel are due to sample position changes during ongoing realignment.

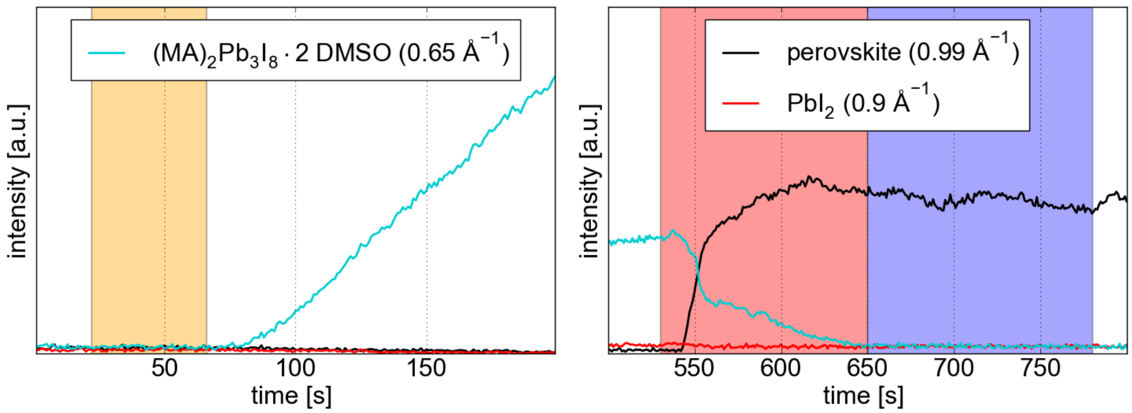

Figure S6: Method: OSC; Composition: $MAPbI_3$. Evolution of the intensity of selected diffraction peaks corresponding to the different phases during the spin-coating (left) and annealing (right) of the sample. The chosen peak position is indicated in the legend. The yellow shaded region in the left panel corresponds to the spinning time and the shaded regions in the right panel correspond to the power of the halogen lamp used for IR annealing (red = 150 W and blue = 85 W).

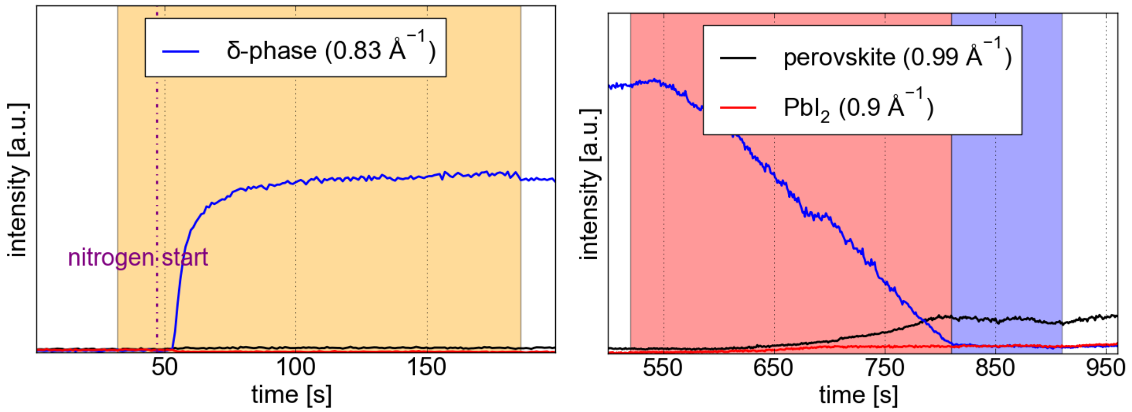

Figure S7: Method: gas-quenching; Composition: $FAPbI_3$. Evolution of the intensity of selected diffraction peaks corresponding to the different phases during the spin-coating (left) and annealing (right) of the sample. The chosen peak position is indicated in the legend. The yellow shaded region in the left panel corresponds to the spinning time and the shaded regions in the right panel correspond to the power of the halogen lamp used for IR annealing (red = 150 W and blue = 85 W). The purple dotted line indicates the start of the nitrogen gas flow.

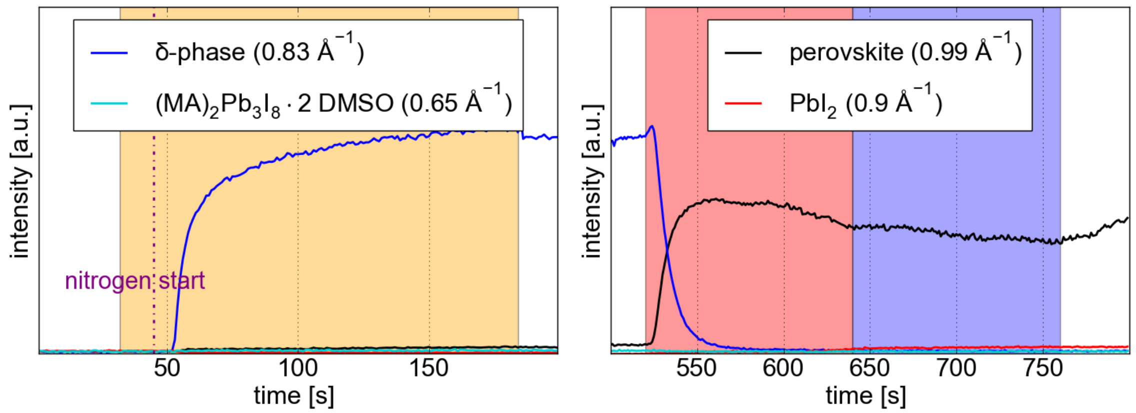

Figure S8: Method: gas-quenching; Composition: $(MA_{0.17}FA_{0.83})_{0.95}Cs_{0.05}PbI_3$. Evolution of the intensity of selected diffraction peaks corresponding to the different phases during the spin-coating (left) and annealing (right) of the sample. The chosen peak position is indicated in the legend. The yellow shaded region in the left panel corresponds to the spinning time and the shaded regions in the right panel correspond to the power of the halogen lamp used for IR annealing (red = 150 W and blue = 85 W). The purple dotted line indicates the start of the nitrogen gas flow.

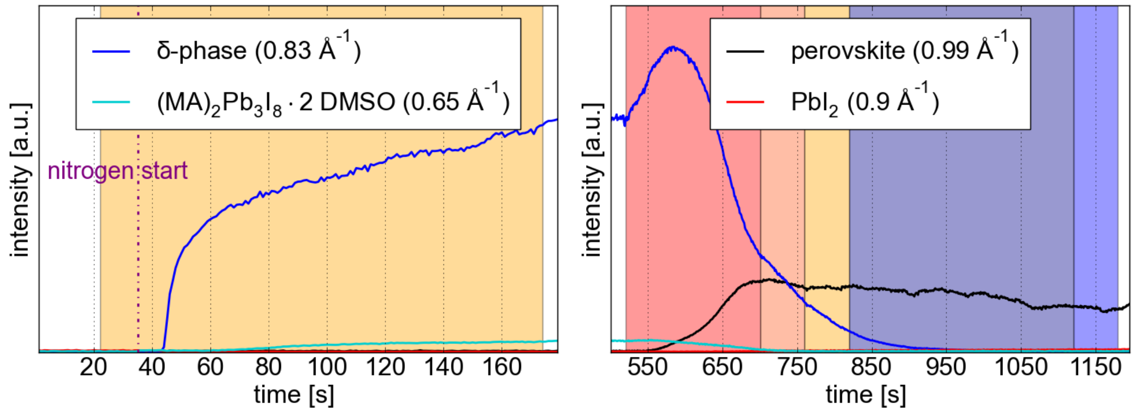

Figure S9: Method: gas-quenching; Composition: $MA_{0.17}FA_{0.83}PbI_3$. Evolution of the intensity of selected diffraction peaks corresponding to the different phases during the spin-coating (left) and annealing (right) of the sample. The chosen peak position is indicated in the legend. The yellow shaded region in the left panel corresponds to the spinning time and the shaded regions in the right panel correspond to the power of the halogen lamp used for IR annealing (red = 150 W, orange = 140 W, yellow = 130 W, darkblue = 120 W and blue = 110 W). The purple dotted line indicates the start of the nitrogen gas flow.

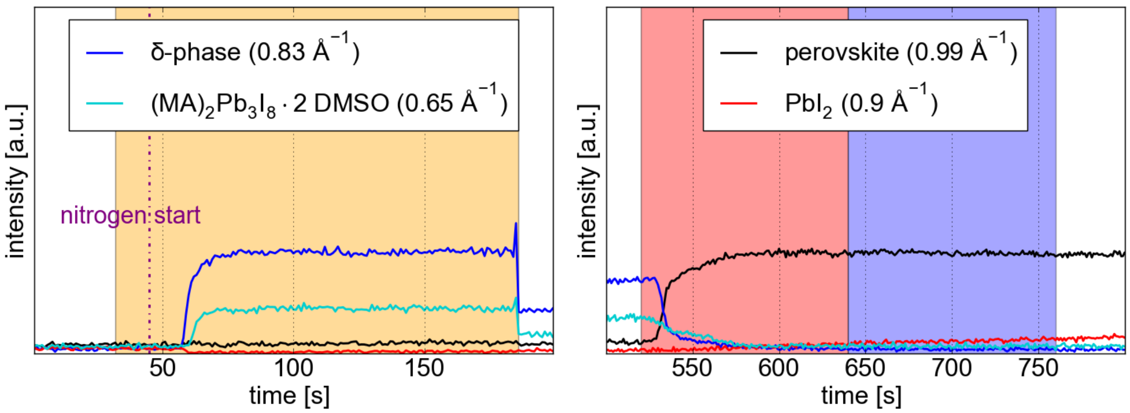

Figure S10: Method: gas-quenching; Composition: $MA_{0.5}FA_{0.5}PbI_3$. Evolution of the intensity of selected diffraction peaks corresponding to the different phases during the spin-coating (left) and annealing (right) of the sample. The chosen peak position is indicated in the legend. The yellow shaded region in the left panel corresponds to the spinning time and the shaded regions in the right panel correspond to the power of the halogen lamp used for IR annealing (red = 150 W and blue = 85 W). The purple dotted line indicates the start of the nitrogen gas flow.

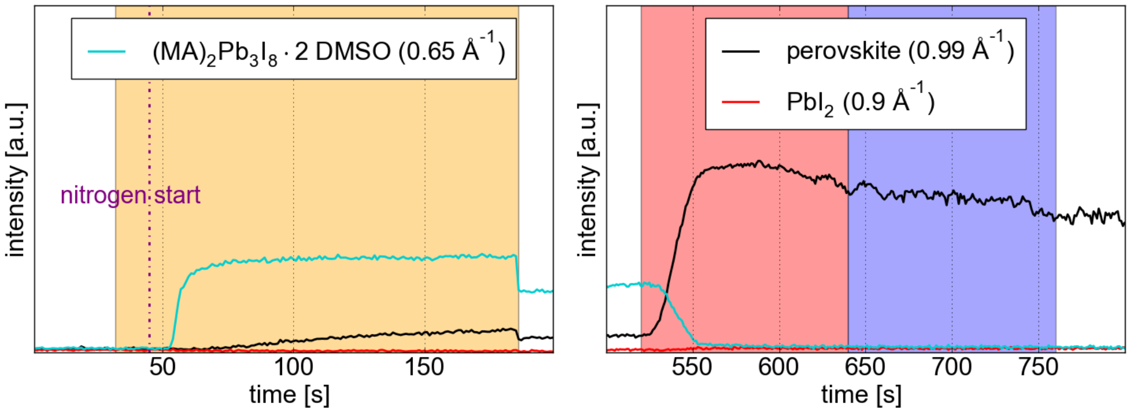

Figure S11: Method: gas-quenching; Composition: $MA_{0.83}FA_{0.17}PbI_3$. Evolution of the intensity of selected diffraction peaks corresponding to the different phases during the spin-coating (left) and annealing (right) of the sample. The chosen peak position is indicated in the legend. The yellow shaded region in the left panel corresponds to the spinning time and the shaded regions in the right panel correspond to the power of the halogen lamp used for IR annealing (red = 150 W and blue = 85 W). The purple dotted line indicates the start of the nitrogen gas flow.

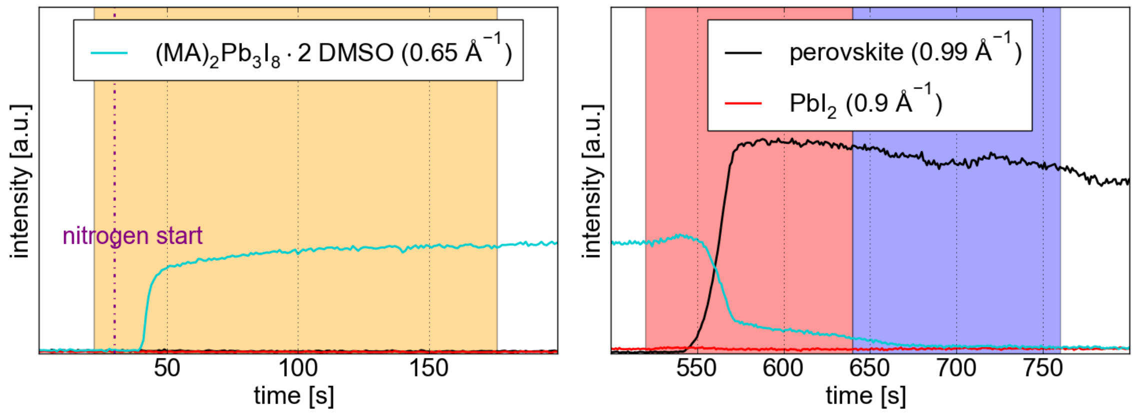

Figure S12: Method: gas-quenching; Composition: $MAPbI_3$. Evolution of the intensity of selected diffraction peaks corresponding to the different phases during the spin-coating (left) and annealing (right) of the sample. The chosen peak position is indicated in the legend. The yellow shaded region in the left panel corresponds to the spinning time and the shaded regions in the right panel correspond to the power of the halogen lamp used for IR annealing (red = 150 W and blue = 85 W). The purple dotted line indicates the start of the nitrogen gas flow.

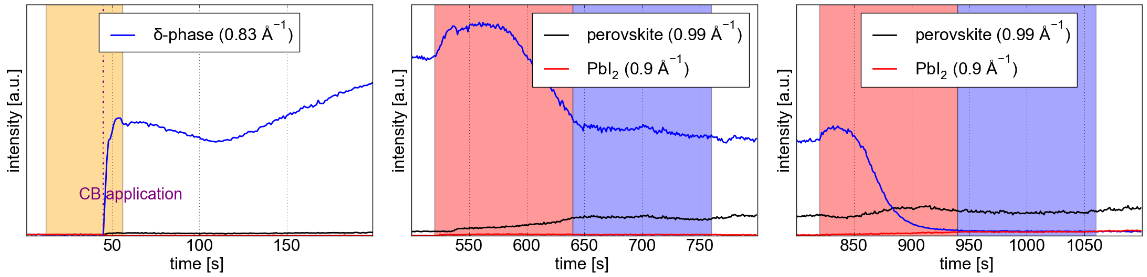

Figure S13: Method: antisolvent (CB); Composition: $FAPbI_3$. Evolution of the intensity of selected diffraction peaks corresponding to the different phases during the spin-coating (left) and annealing (middle and right) of the sample. The chosen peak position is indicated in the legend. The yellow shaded region in the left panel corresponds to the spinning time and the shaded regions in the right panel correspond to the power of the halogen lamp used for IR annealing (red = 150 W and blue = 85 W). A second annealing step was done to fully convert the δ-phase to pervoskite. The purple dotted line marks the antisolvent application.

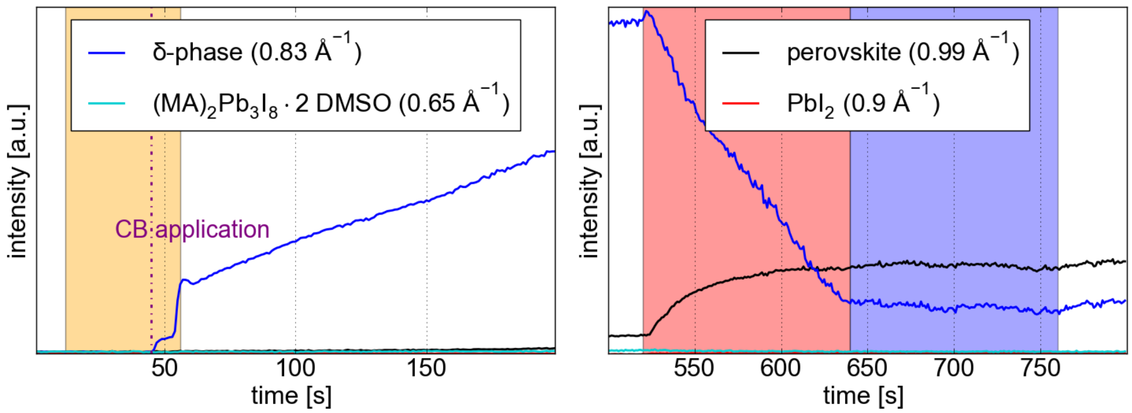

Figure S14: Method: antisolvent (CB); Composition: $(MA_{0.17}FA_{0.83})_{0.95}Cs_{0.05}PbI_3$. Evolution of the intensity of selected diffraction peaks corresponding to the different phases during the spin-coating (left) and annealing (right) of the sample. The chosen peak position is indicated in the legend. The yellow shaded region in the left panel corresponds to the spinning time and the shaded regions in the right panel correspond to the power of the halogen lamp used for IR annealing (red = 150 W and blue = 85 W). The purple dotted line marks the antisolvent application.

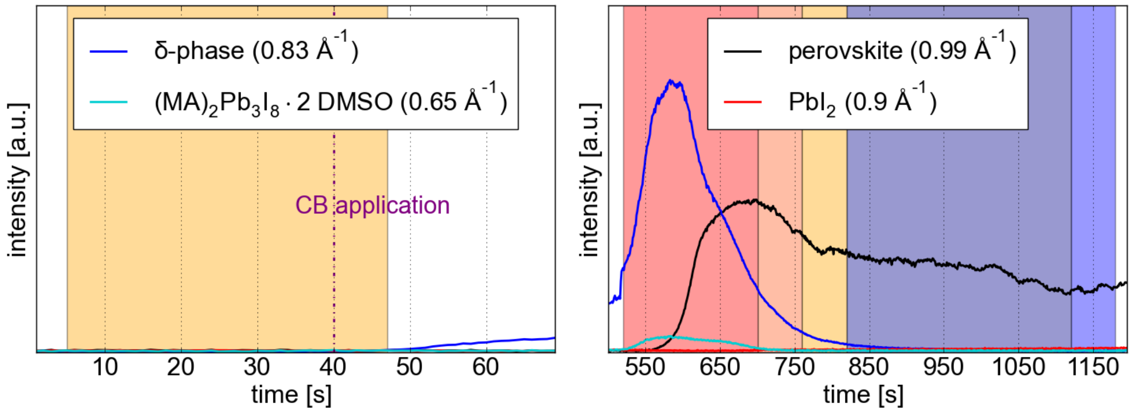

Figure S15: Method: antisolvent (CB); Composition: $MA_{0.17}FA_{0.83}PbI_3$. Evolution of the intensity of selected diffraction peaks corresponding to the different phases during the spin-coating (left) and annealing (right) of the sample. The chosen peak position is indicated in the legend. The yellow shaded region in the left panel corresponds to the spinning time and the shaded regions in the right panel correspond to the power of the halogen lamp used for IR annealing (red = 150 W, orange = 140 W, yellow = 130 W, darkblue = 120 W and blue = 110 W). The purple dotted line marks the antisolvent application.

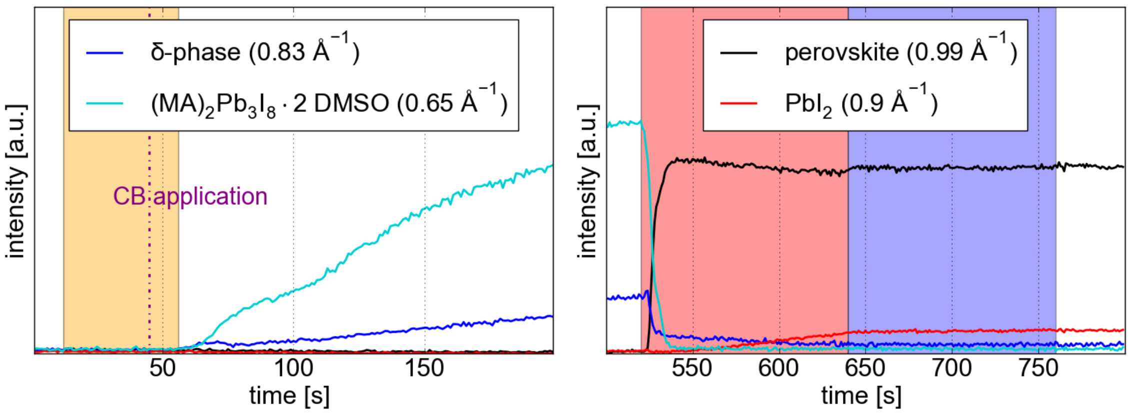

Figure S16: Method: antisolvent (CB); Composition: $MA_{0.5}FA_{0.5}PbI_3$. Evolution of the intensity of selected diffraction peaks corresponding to the different phases during the spin-coating (left) and annealing (right) of the sample. The chosen peak position is indicated in the legend. The yellow shaded region in the left panel corresponds to the spinning time and the shaded regions in the right panel correspond to the power of the halogen lamp used for IR annealing (red = 150 W and blue = 85 W). The purple dotted line marks the antisolvent application.

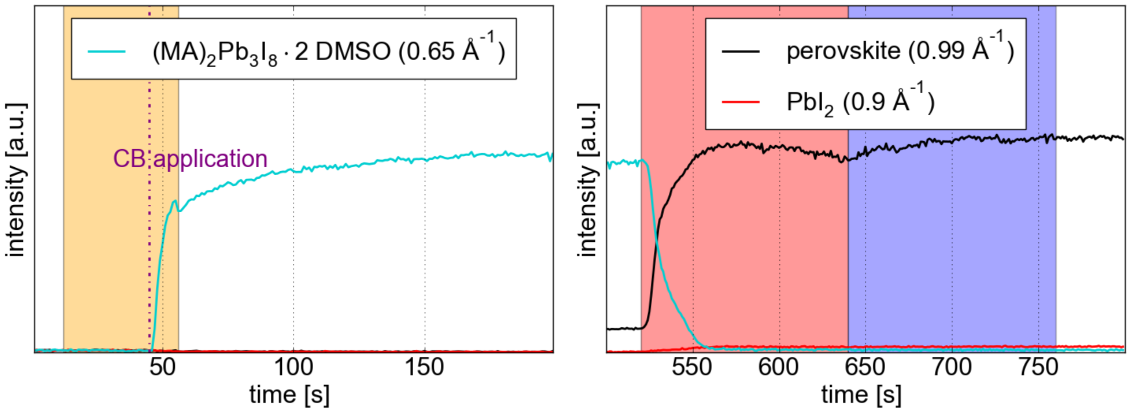

Figure S17: Method: antisolvent (CB); Composition: $MA_{0.83}FA_{0.17}PbI_3$. Evolution of the intensity of selected diffraction peaks corresponding to the different phases during the spin-coating (left) and annealing (right) of the sample. The chosen peak position is indicated in the legend. The yellow shaded region in the left panel corresponds to the spinning time and the shaded regions in the right panel correspond to the power of the halogen lamp used for IR annealing (red = 150 W and blue = 85 W). The purple dotted line marks the antisolvent application.

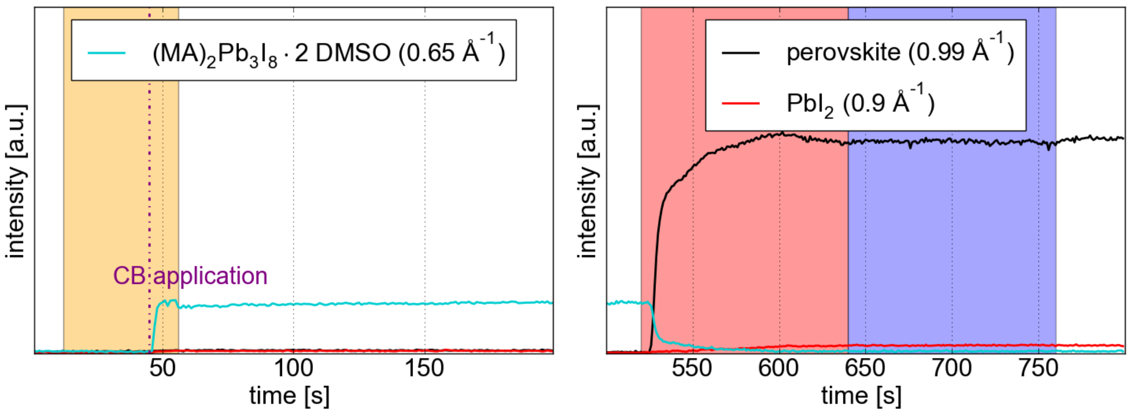

Figure S18: Method: antisolvent (CB); Composition: $MAPbI_3$. Evolution of the intensity of selected diffraction peaks corresponding to the different phases during the spin-coating (left) and annealing (right) of the sample. The chosen peak position is indicated in the legend. The yellow shaded region in the left panel corresponds to the spinning time and the shaded regions in the right panel correspond to the power of the halogen lamp used for IR annealing (red = 150 W and blue = 85 W). The purple dotted line marks the antisolvent application.

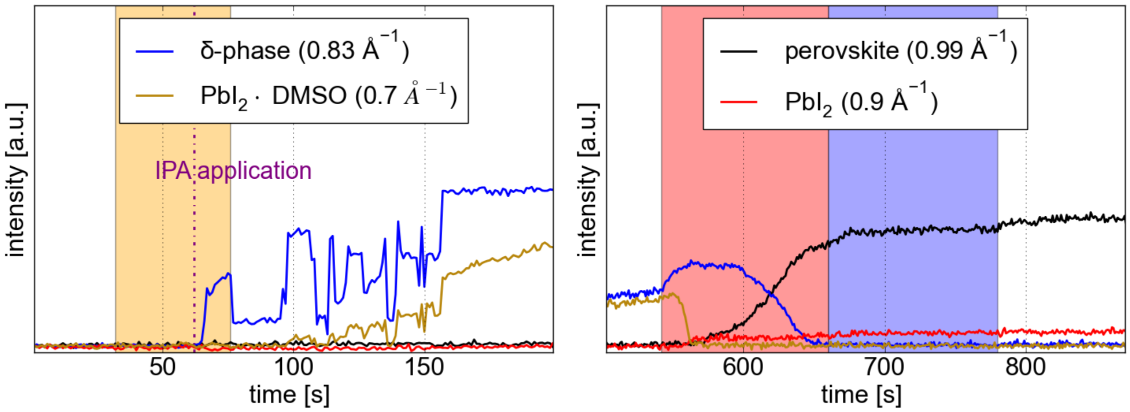

Figure S19: Method: antisolvent (IPA); Composition: $FAPbI_3$. Evolution of the intensity of selected diffraction peaks corresponding to the different phases during the spin-coating (left) and annealing (right) of the sample. The chosen peak position is indicated in the legend. The yellow shaded region in the left panel corresponds to the spinning time and the shaded regions in the right panel correspond to the power of the halogen lamp used for IR annealing (red = 150 W and blue = 85 W). The purple dotted line marks the antisolvent application. Intensity jumps after 90 s in the left panel are due to sample position changes during ongoing realignment.

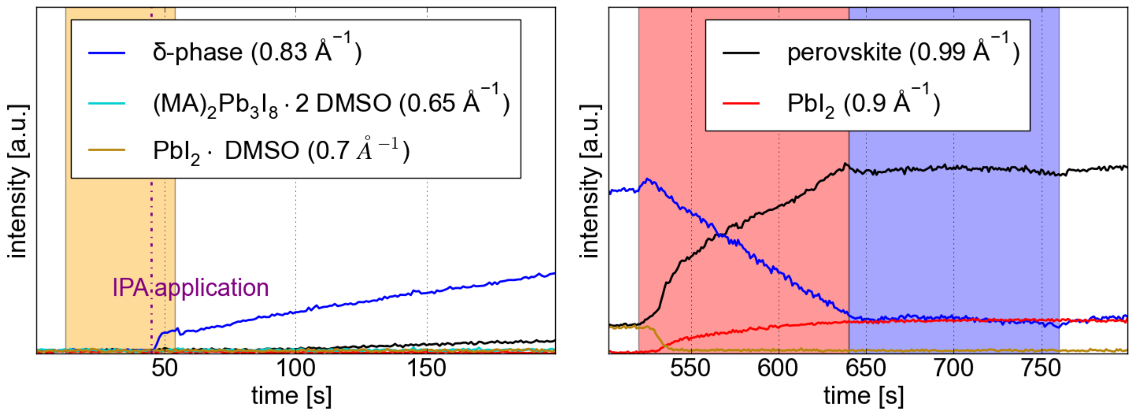

Figure S20: Method: antisolvent (IPA); Composition: $(MA_{0.17}FA_{0.83})_{0.95}Cs_{0.05}PbI_3$. Evolution of the intensity of selected diffraction peaks corresponding to the different phases during the spin-coating (left) and annealing (right) of the sample. The chosen peak position is indicated in the legend. The yellow shaded region in the left panel corresponds to the spinning time and the shaded regions in the right panel correspond to the power of the halogen lamp used for IR annealing (red = 150 W and blue = 85 W). The purple dotted line marks the antisolvent application.

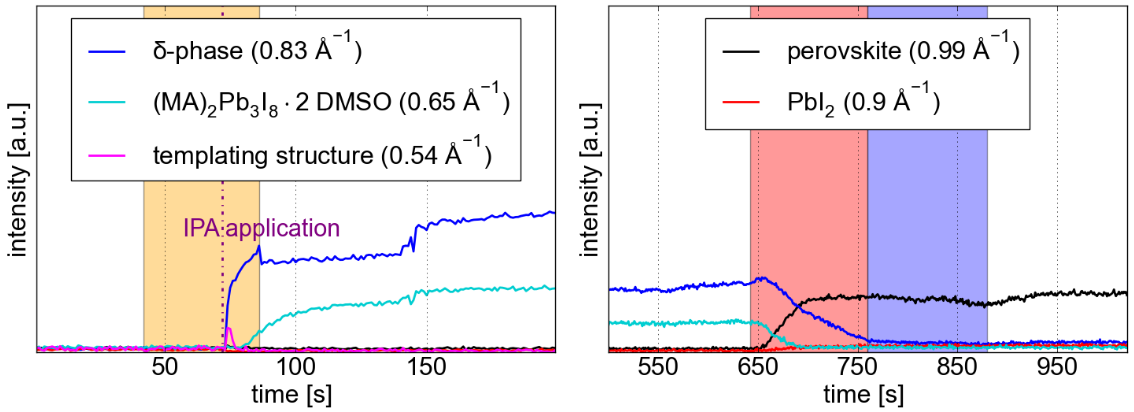

Figure S21: Method: antisolvent (IPA); Composition: $MA_{0.17}FA_{0.83}PbI_3$. Evolution of the intensity of selected diffraction peaks corresponding to the different phases during the spin-coating (left) and annealing (right) of the sample. The chosen peak position is indicated in the legend. The yellow shaded region in the left panel corresponds to the spinning time and the shaded regions in the right panel correspond to the power of the halogen lamp used for IR annealing (red = 150 W and blue = 85 W). The purple dotted line marks the antisolvent application.

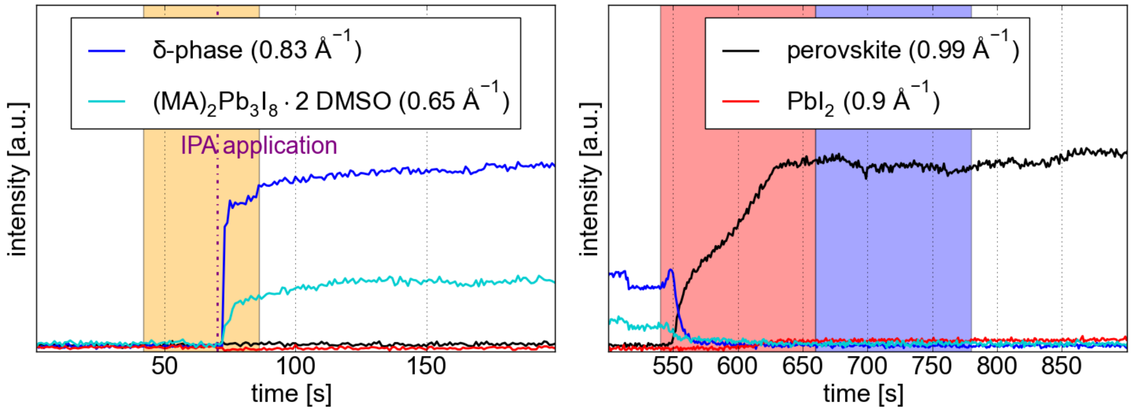

Figure S22: Method: antisolvent (IPA); Composition: $MA_{0.5}FA_{0.5}PbI_3$. Evolution of the intensity of selected diffraction peaks corresponding to the different phases during the spin-coating (left) and annealing (right) of the sample. The chosen peak position is indicated in the legend. The yellow shaded region in the left panel corresponds to the spinning time and the shaded regions in the right panel correspond to the power of the halogen lamp used for IR annealing (red = 150 W and blue = 85 W). The purple dotted line marks the antisolvent application.

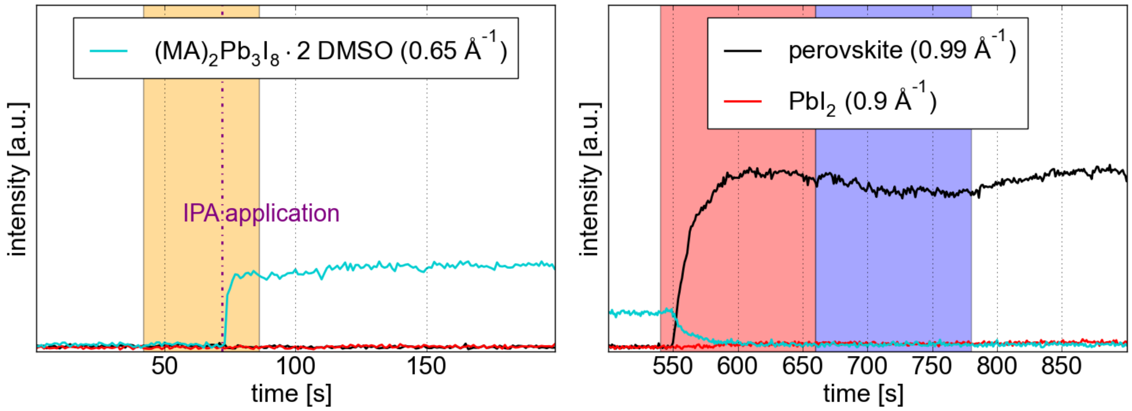

Figure S23: Method: antisolvent (IPA); Composition: $MA_{0.83}FA_{0.17}PbI_3$. Evolution of the intensity of selected diffraction peaks corresponding to the different phases during the spin-coating (left) and annealing (right) of the sample. The chosen peak position is indicated in the legend. The yellow shaded region in the left panel corresponds to the spinning time and the shaded regions in the right panel correspond to the power of the halogen lamp used for IR annealing (red = 150 W and blue = 85 W). The purple dotted line marks the antisolvent application.

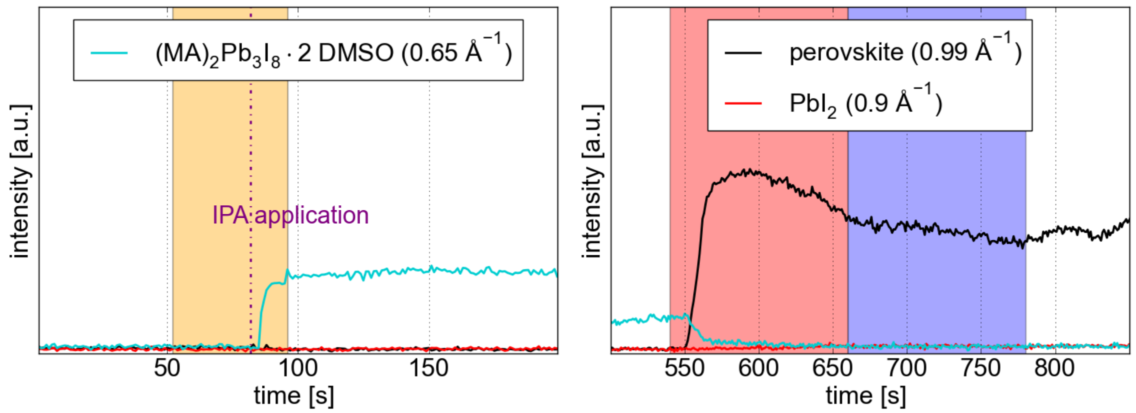

Figure S24: Method: antisolvent (IPA); Composition: $MAPbI_3$. Evolution of the intensity of selected diffraction peaks corresponding to the different phases during the spin-coating (left) and annealing (right) of the sample. The chosen peak position is indicated in the legend. The yellow shaded region in the left panel corresponds to the spinning time and the shaded regions in the right panel correspond to the power of the halogen lamp used for IR annealing (red = 150 W and blue = 85 W). The purple dotted line marks the antisolvent application.

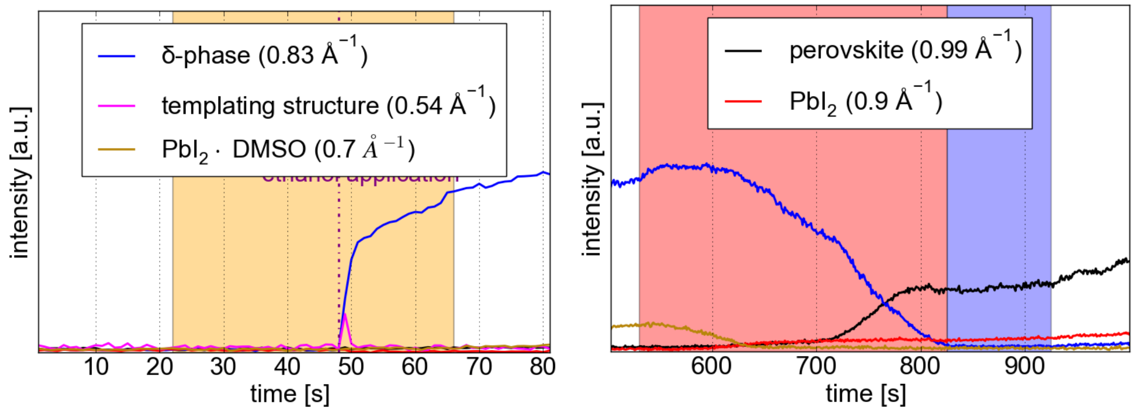

Figure S25: Method: antisolvent (EtOH); Composition: $FAPbI_3$. Evolution of the intensity of selected diffraction peaks corresponding to the different phases during the spin-coating (left) and annealing (right) of the sample. The chosen peak position is indicated in the legend. The yellow shaded region in the left panel corresponds to the spinning time and the shaded regions in the right panel correspond to the power of the halogen lamp used for IR annealing (red = 150 W and blue = 85 W). The purple dotted line marks the antisolvent application.

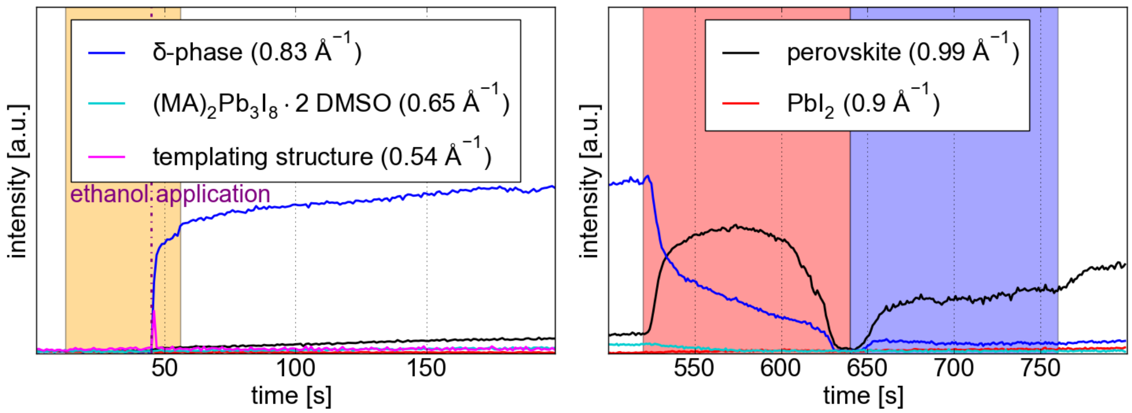

Figure S26: Method: antisolvent (EtOH); Composition: $(MA_{0.17}FA_{0.83})_{0.95}Cs_{0.05}PbI_3$. Evolution of the intensity of selected diffraction peaks corresponding to the different phases during the spin-coating (left) and annealing (right) of the sample. The chosen peak position is indicated in the legend. The yellow shaded region in the left panel corresponds to the spinning time and the shaded regions in the right panel correspond to the power of the halogen lamp used for IR annealing (red = 150 W and blue = 85 W). The purple dotted line marks the antisolvent application. The loss of intensity towards 640 s in the right panel is due to severe sample misalignment.

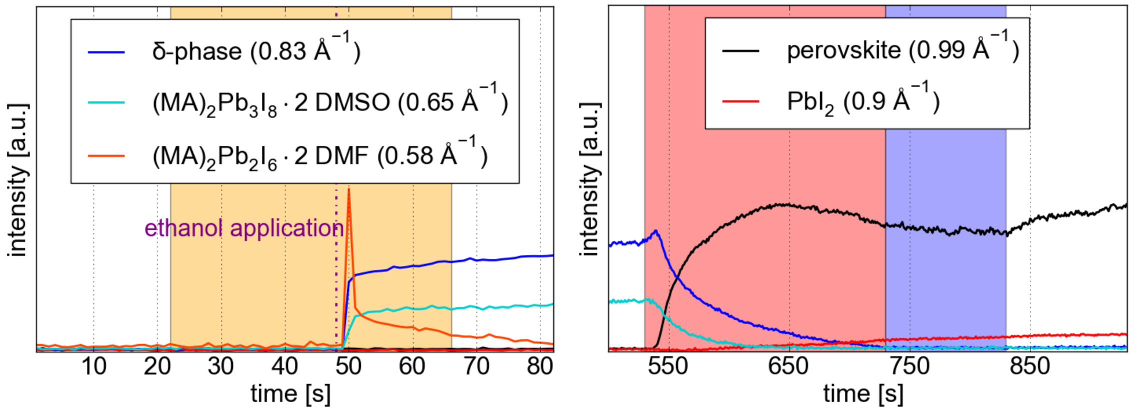

Figure S27: Method: antisolvent (EtOH); Composition: $MA_{0.5}FA_{0.5}PbI_3$. Evolution of the intensity of selected diffraction peaks corresponding to the different phases during the spin-coating (left) and annealing (right) of the sample. The chosen peak position is indicated in the legend. The yellow shaded region in the left panel corresponds to the spinning time and the shaded regions in the right panel correspond to the power of the halogen lamp used for IR annealing (red = 150 W and blue = 85 W). The purple dotted line marks the antisolvent application.

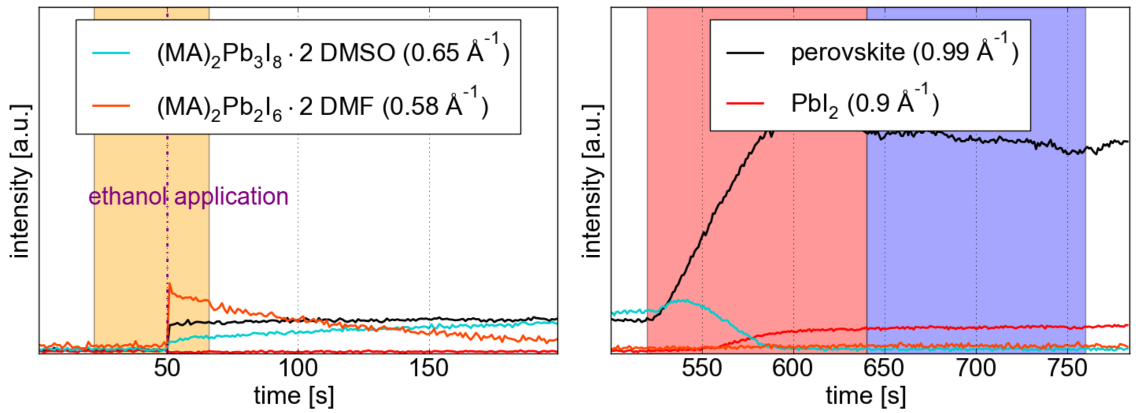

Figure S28: Method: antisolvent (EtOH); Composition: $MA_{0.83}FA_{0.17}PbI_3$. Evolution of the intensity of selected diffraction peaks corresponding to the different phases during the spin-coating (left) and annealing (right) of the sample. The chosen peak position is indicated in the legend. The yellow shaded region in the left panel corresponds to the spinning time and the shaded regions in the right panel correspond to the power of the halogen lamp used for IR annealing (red = 150 W and blue = 85 W). The purple dotted line marks the antisolvent application.

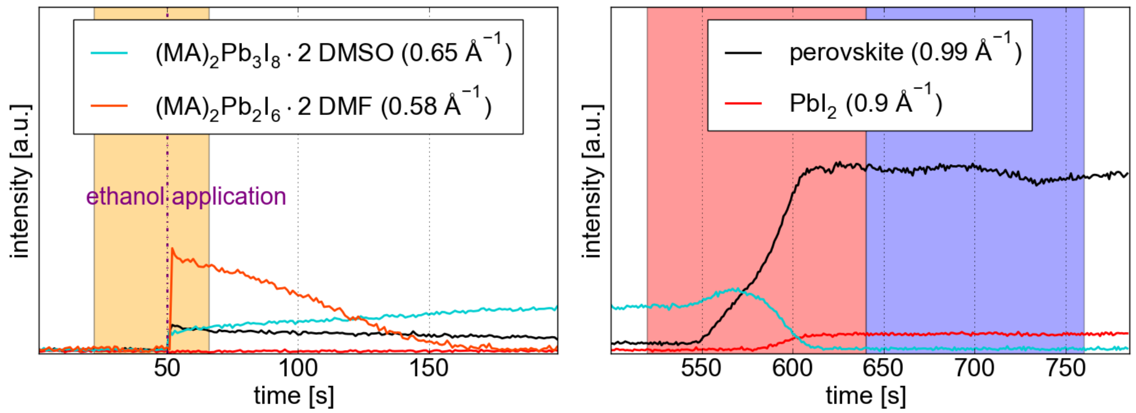

Figure S29: Method: antisolvent (EtOH); Composition: $MAPbI_3$. Evolution of the intensity of selected diffraction peaks corresponding to the different phases during the spin-coating (left) and annealing (right) of the sample. The chosen peak position is indicated in the legend. The yellow shaded region in the left panel corresponds to the spinning time and the shaded regions in the right panel correspond to the power of the halogen lamp used for IR annealing (red = 150 W and blue = 85 W). The purple dotted line marks the antisolvent application.

Table S1: Crystal structures detected in the annealed thin films, presented as relative phase fractions of perovskite (black, top row), PbI_2 (red, second row) and the δ-phase (blue, third row if present) in percentages.

[%]	OSC	Nitrogen	CB	IPA	Ethanol
FA	87	59	63	72	64
	13	38	37	28	36
	0	3	0	0	0
Triple Cation	89	94	93	67	88
	9	6	5	22	9
	2	0	2	11	3
x = 0.17	92	90	94	69	80
	8	10	6	27	20
	0	0	0	4	0
x = 0.5	90	78	60	88	76
	10	22	40	12	24
x = 0.83	91	92	91	88	69
	9	8	9	12	31
MA	89	92	77	89	79
	11	8	23	11	21

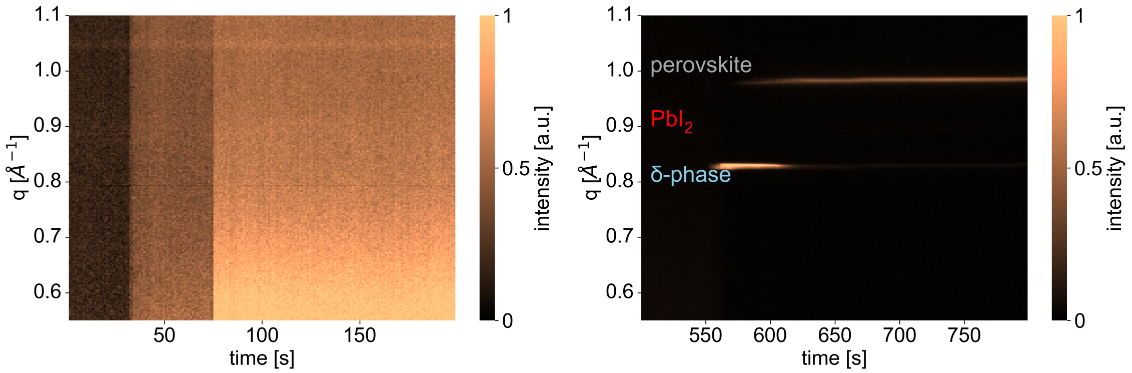

Figure S30: Method: OSC; Composition: $FAPbI_3$. Pseudo-2D XRD data in a selected q-range showing the signals of interest, extracted from the in-situ GIWAXS data during the spin-coating (left) and annealing (right) to follow the crystallization of the sample. The phases corresponding to the peaks visible, are stated on the left side of the figures.

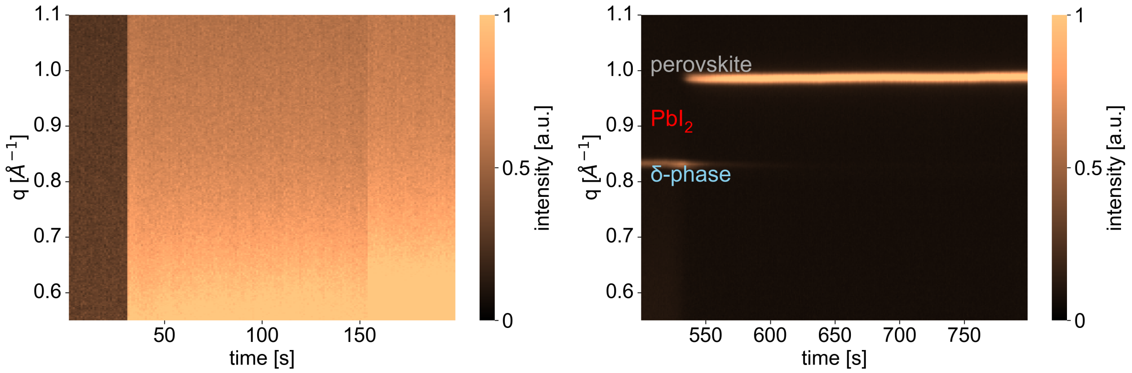

Figure S31: Method: OSC; Composition: $(MA_{0.17}FA_{0.83})_{0.95}Cs_{0.05}PbI_3$. Pseudo-2D XRD data in a selected q-range showing the signals of interest, extracted from the in-situ GIWAXS data during the spin-coating (left) and annealing (right) to follow the crystallization of the sample. The phases corresponding to the peaks visible, are stated on the left side of the figures.

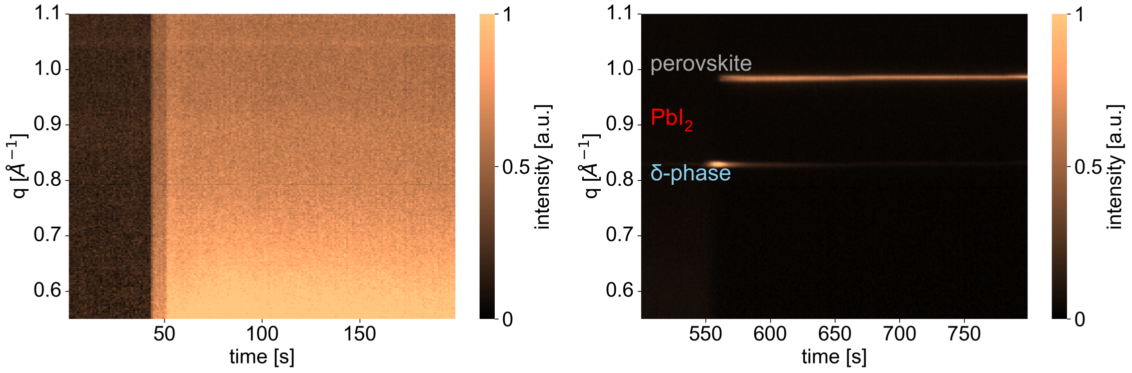

Figure S32: Method: OSC; Composition: $MA_{0.17}FA_{0.83}PbI_3$. Pseudo-2D XRD data in a selected q-range showing the signals of interest, extracted from the in-situ GIWAXS data during the spin-coating (left) and annealing (right) to follow the crystallization of the sample. The phases corresponding to the peaks visible, are stated on the left side of the figures.

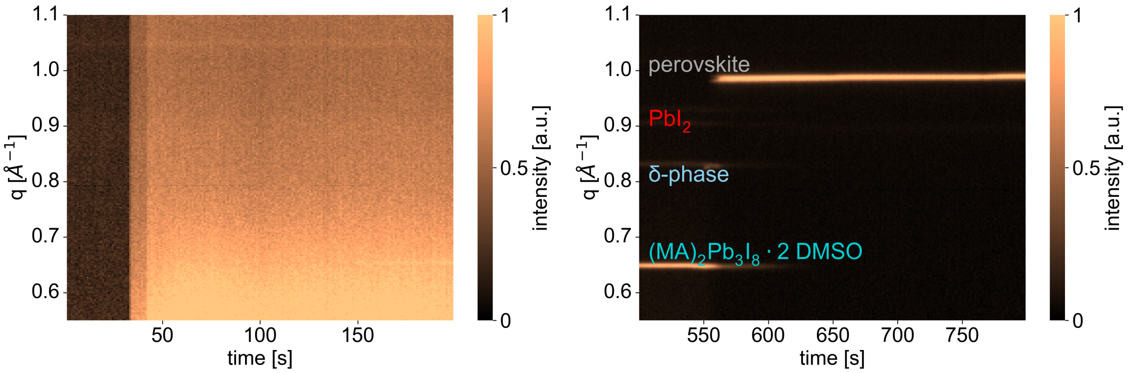

Figure S33: Method: OSC; Composition: $MA_{0.5}FA_{0.5}PbI_3$. Pseudo-2D XRD data in a selected q-range showing the signals of interest, extracted from the in-situ GIWAXS data during the spin-coating (left) and annealing (right) to follow the crystallization of the sample. The phases corresponding to the peaks visible, are stated on the left side of the figures.

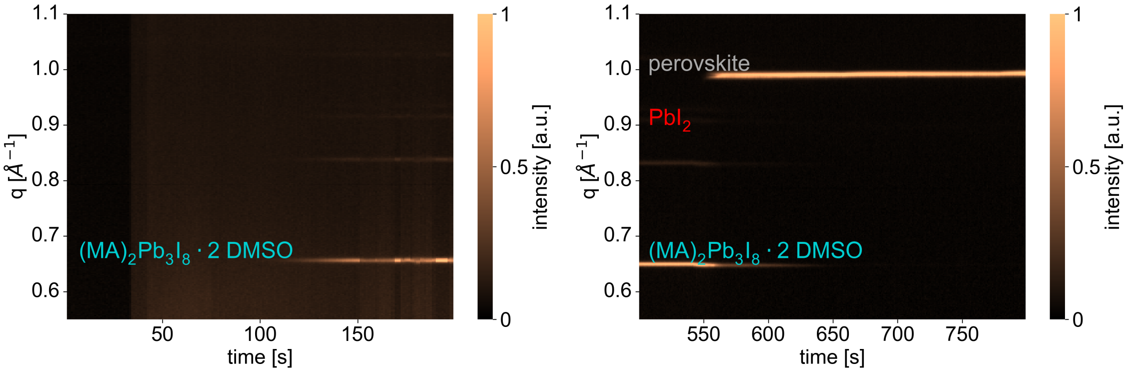

Figure S34: Method: OSC; Composition: $MA_{0.83}FA_{0.17}PbI_3$. Pseudo-2D XRD data in a selected q-range showing the signals of interest, extracted from the in-situ GIWAXS data during the spin-coating (left) and annealing (right) to follow the crystallization of the sample. The phases corresponding to the peaks visible, are stated on the left side of the figures.

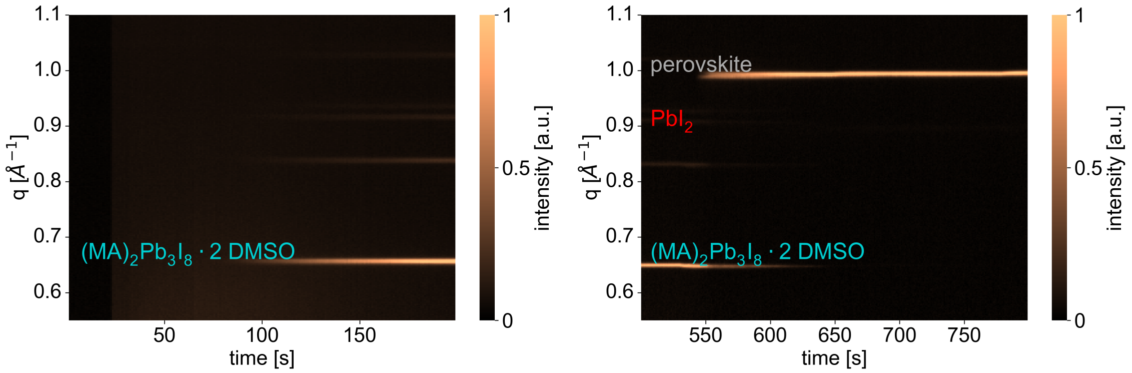

Figure S35: Method: OSC; Composition: $MAPbI_3$. Pseudo-2D XRD data in a selected q-range showing the signals of interest, extracted from the in-situ GIWAXS data during the spin-coating (left) and annealing (right) to follow the crystallization of the sample. The phases corresponding to the peaks visible, are stated on the left side of the figures.

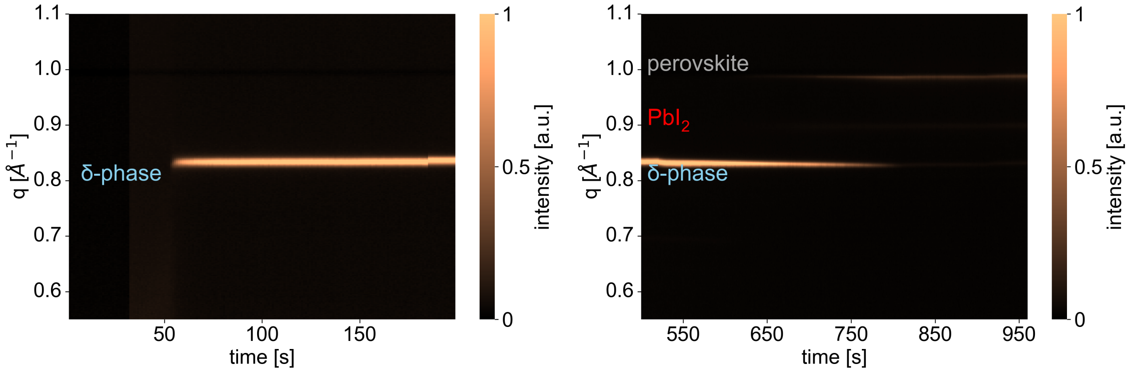

Figure S36: Method: gas-quenching; Composition: $FAPbI_3$. Pseudo-2D XRD data in a selected q-range showing the signals of interest, extracted from the in-situ GIWAXS data during the spin-coating (left) and annealing (right) to follow the crystallization of the sample. The phases corresponding to the peaks visible, are stated on the left side of the figures.

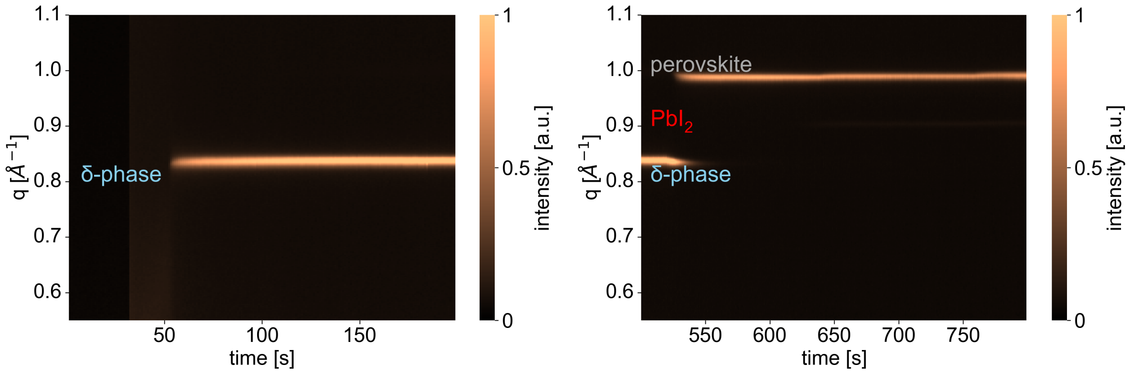

Figure S37: Method: gas-quenching; Composition: $(MA_{0.17}FA_{0.83})_{0.95}Cs_{0.05}PbI_3$. Pseudo-2D XRD data in a selected q-range showing the signals of interest, extracted from the in-situ GIWAXS data during the spin-coating (left) and annealing (right) to follow the crystallization of the sample. The phases corresponding to the peaks visible, are stated on the left side of the figures.

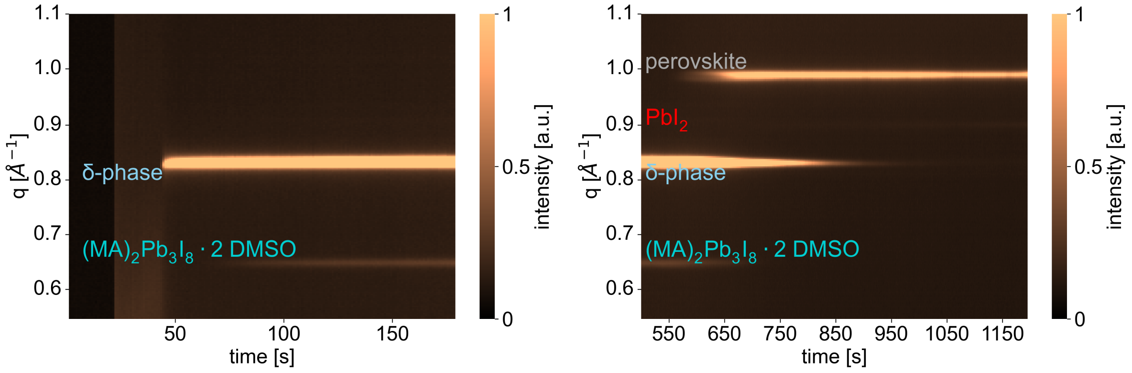

Figure S38: Method: gas-quenching; Composition: $MA_{0.17}FA_{0.83}PbI_3$. Pseudo-2D XRD data in a selected q-range showing the signals of interest, extracted from the in-situ GIWAXS data during the spin-coating (left) and annealing (right) to follow the crystallization of the sample. The phases corresponding to the peaks visible, are stated on the left side of the figures.

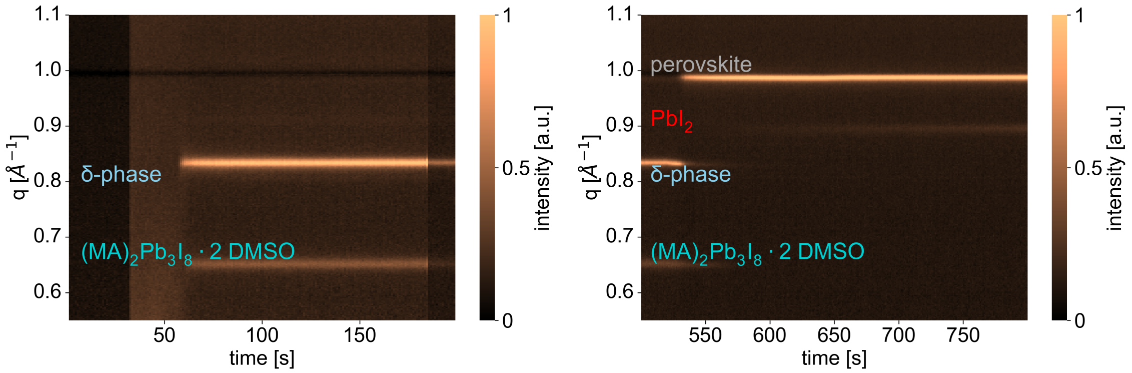

Figure S39: Method: gas-quenching; Composition: $MA_{0.5}FA_{0.5}PbI_3$. Pseudo-2D XRD data in a selected q-range showing the signals of interest, extracted from the in-situ GIWAXS data during the spin-coating (left) and annealing (right) to follow the crystallization of the sample. The phases corresponding to the peaks visible, are stated on the left side of the figures.

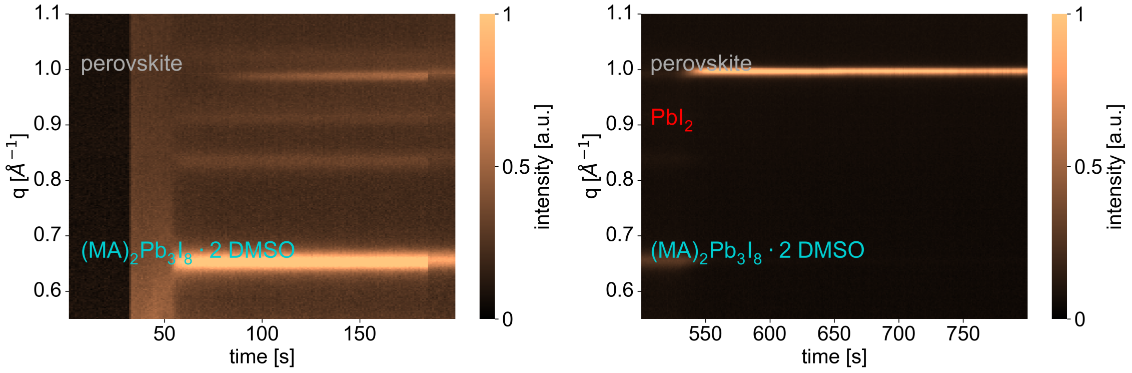

Figure S40: Method: gas-quenching; Composition: $MA_{0.83}FA_{0.17}PbI_3$. Pseudo-2D XRD data in a selected q-range showing the signals of interest, extracted from the in-situ GIWAXS data during the spin-coating (left) and annealing (right) to follow the crystallization of the sample. The phases corresponding to the peaks visible, are stated on the left side of the figures.

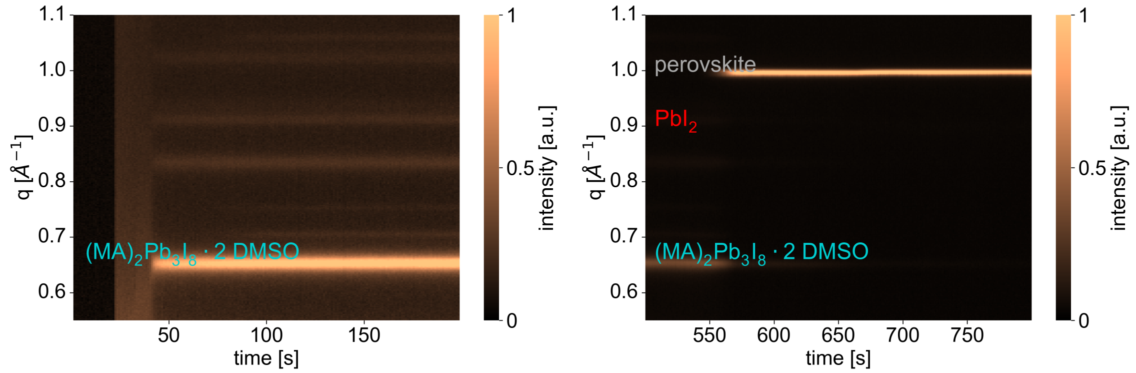

Figure S41: Method: gas-quenching; Composition: $MAPbI_3$. Pseudo-2D XRD data in a selected q-range showing the signals of interest, extracted from the in-situ GIWAXS data during the spin-coating (left) and annealing (right) to follow the crystallization of the sample. The phases corresponding to the peaks visible, are stated on the left side of the figures.

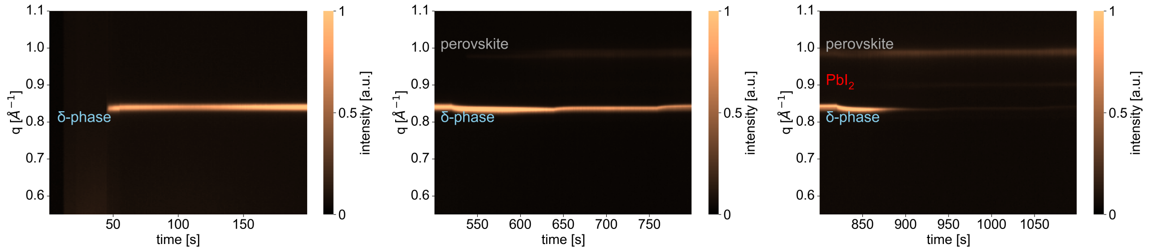

Figure S42: Method: antisolvent (CB); Composition: $FAPbI_3$. Pseudo-2D XRD data in a selected q-range showing the signals of interest, extracted from the in-situ GIWAXS data during the spin-coating (left) and annealing (right) to follow the crystallization of the sample. The phases corresponding to the peaks visible, are stated on the left side of the figures.

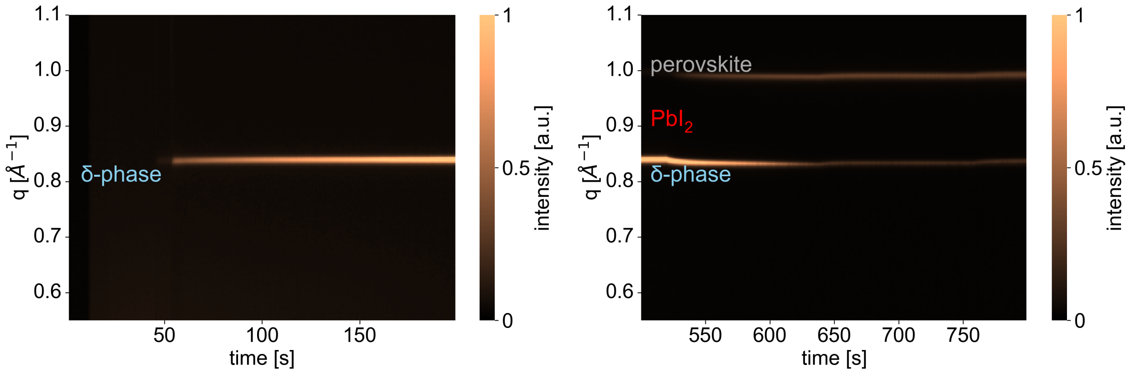

Figure S43: Method: antisolvent (CB); Composition: $(MA_{0.17}FA_{0.83})_{0.95}Cs_{0.05}PbI_3$. Pseudo-2D XRD data in a selected q-range showing the signals of interest, extracted from the in-situ GIWAXS data during the spin-coating (left) and annealing (right) to follow the crystallization of the sample. The phases corresponding to the peaks visible, are stated on the left side of the figures.

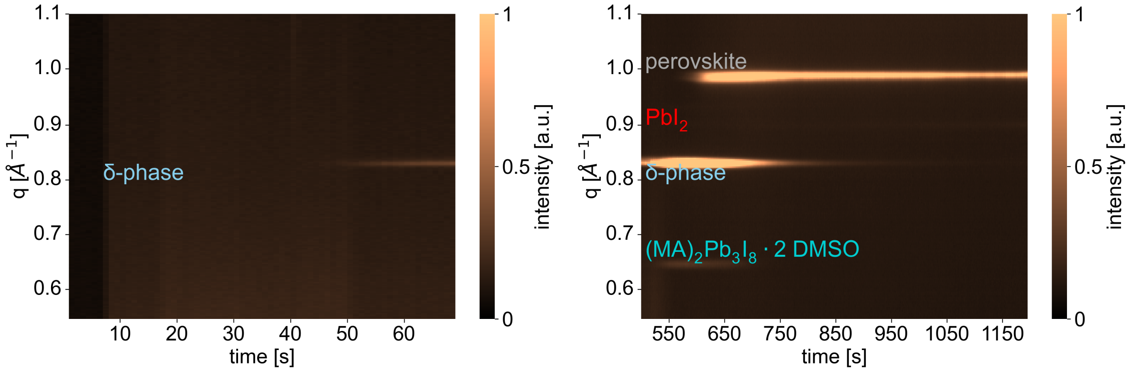

Figure S44: Method: antisolvent (CB); Composition: $MA_{0.17}FA_{0.83}PbI_3$. Pseudo-2D XRD data in a selected q-range showing the signals of interest, extracted from the in-situ GIWAXS data during the spin-coating (left) and annealing (right) to follow the crystallization of the sample. The phases corresponding to the peaks visible, are stated on the left side of the figures.

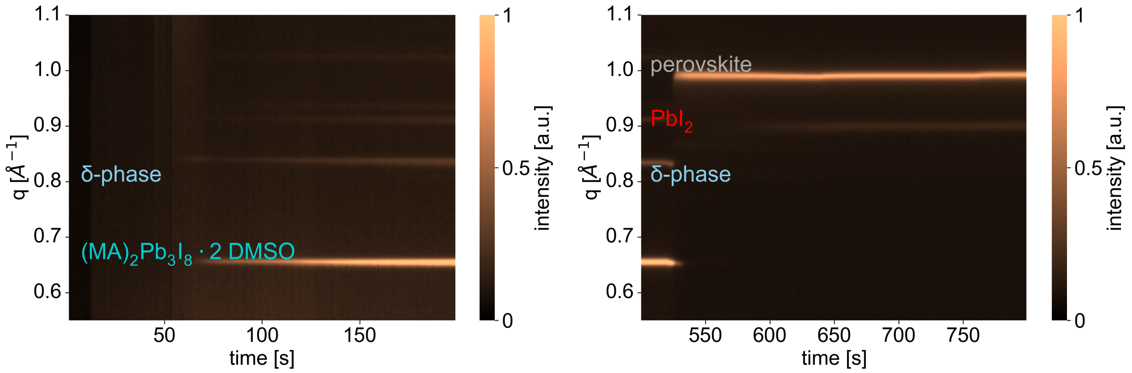

Figure S45: Method: antisolvent (CB); Composition: $MA_{0.5}FA_{0.5}PbI_3$. Pseudo-2D XRD data in a selected q-range showing the signals of interest, extracted from the in-situ GIWAXS data during the spin-coating (left) and annealing (right) to follow the crystallization of the sample. The phases corresponding to the peaks visible, are stated on the left side of the figures.

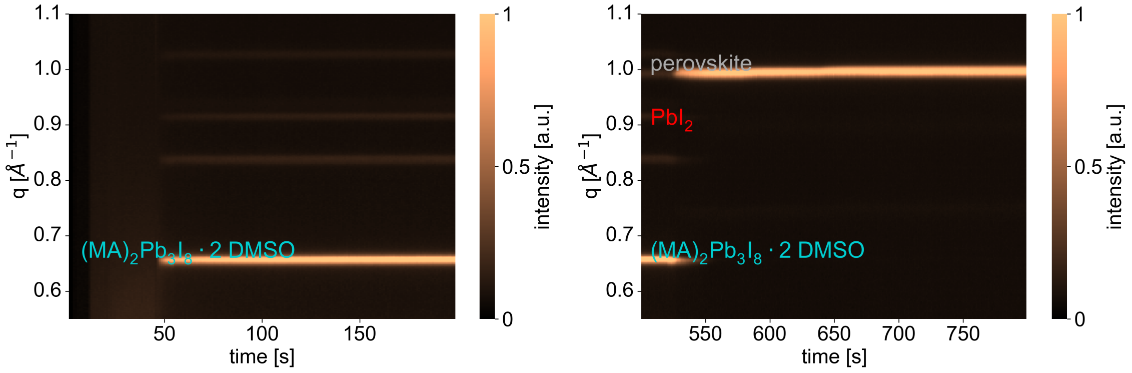

Figure S46: Method: antisolvent (CB); Composition: $MA_{0.83}FA_{0.17}PbI_3$. Pseudo-2D XRD data in a selected q-range showing the signals of interest, extracted from the in-situ GIWAXS data during the spin-coating (left) and annealing (right) to follow the crystallization of the sample. The phases corresponding to the peaks visible, are stated on the left side of the figures.

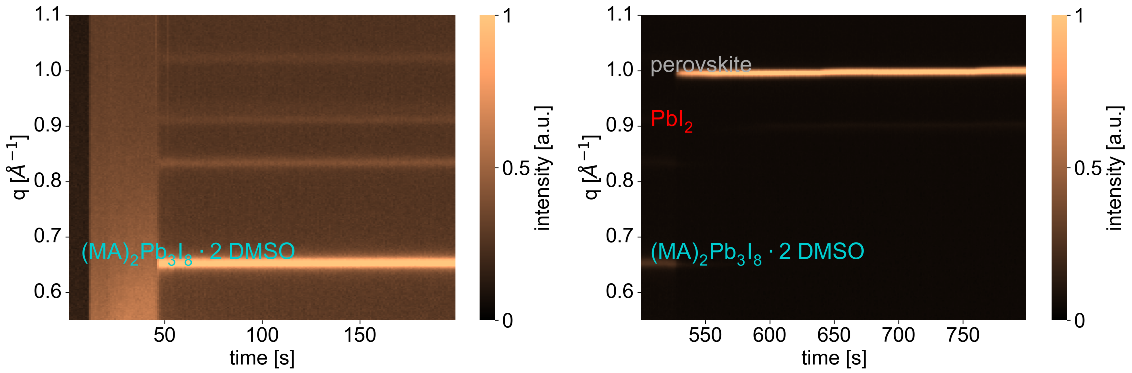

Figure S47: Method: antisolvent (CB); Composition: $MAPbI_3$. Pseudo-2D XRD data in a selected q-range showing the signals of interest, extracted from the in-situ GIWAXS data during the spin-coating (left) and annealing (right) to follow the crystallization of the sample. The phases corresponding to the peaks visible, are stated on the left side of the figures.

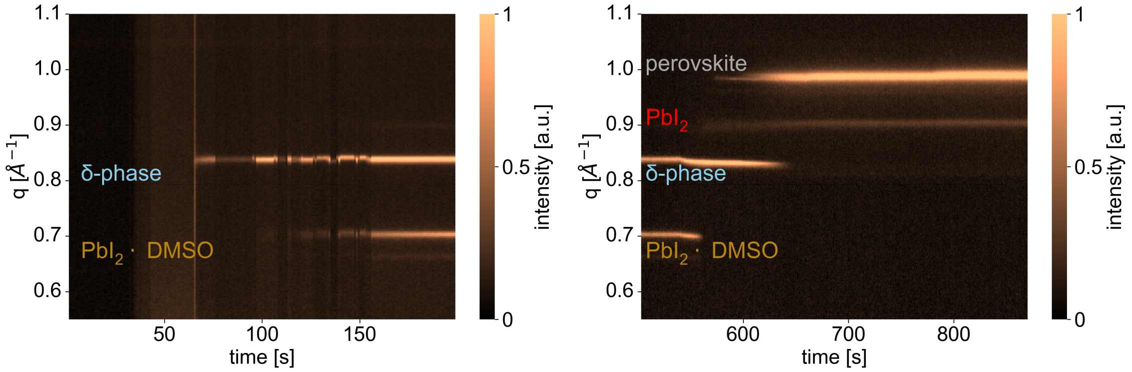

Figure S48: Method: antisolvent (IPA); Composition: $FAPbI_3$. Pseudo-2D XRD data in a selected q-range showing the signals of interest, extracted from the in-situ GIWAXS data during the spin-coating (left) and annealing (right) to follow the crystallization of the sample. The phases corresponding to the peaks visible, are stated on the left side of the figures.

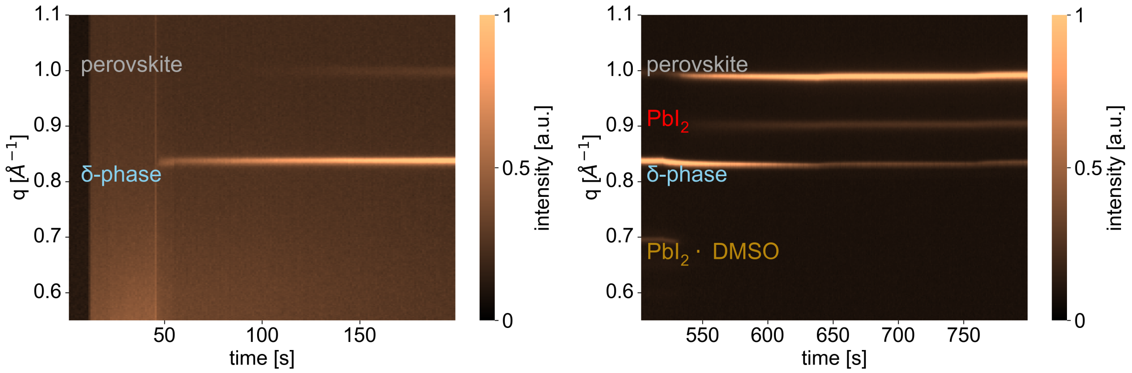

Figure S49: Method: antisolvent (IPA); Composition: $(MA_{0.17}FA_{0.83})_{0.95}Cs_{0.05}PbI_3$. Pseudo-2D XRD data in a selected q-range showing the signals of interest, extracted from the in-situ GIWAXS data during the spin-coating (left) and annealing (right) to follow the crystallization of the sample. The phases corresponding to the peaks visible, are stated on the left side of the figures.

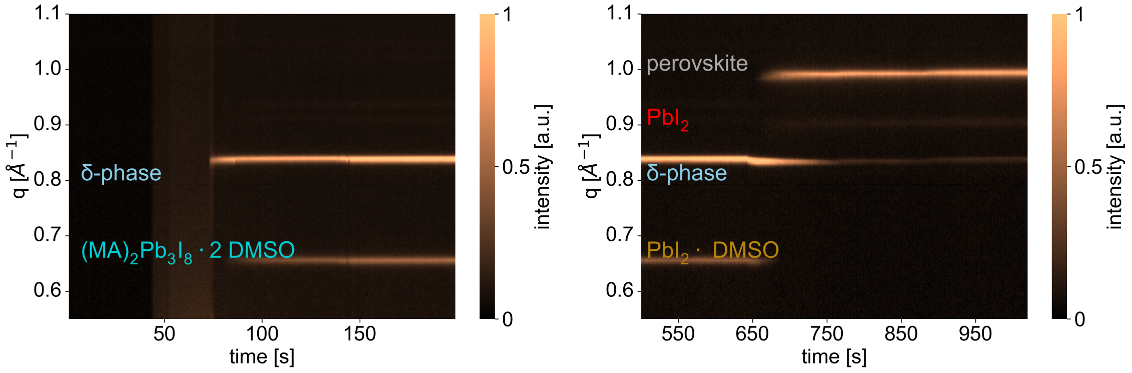

Figure S50: Method: antisolvent (IPA); Composition: $MA_{0.17}FA_{0.83}PbI_3$. Pseudo-2D XRD data in a selected q-range showing the signals of interest, extracted from the in-situ GIWAXS data during the spin-coating (left) and annealing (right) to follow the crystallization of the sample. The phases corresponding to the peaks visible, are stated on the left side of the figures.

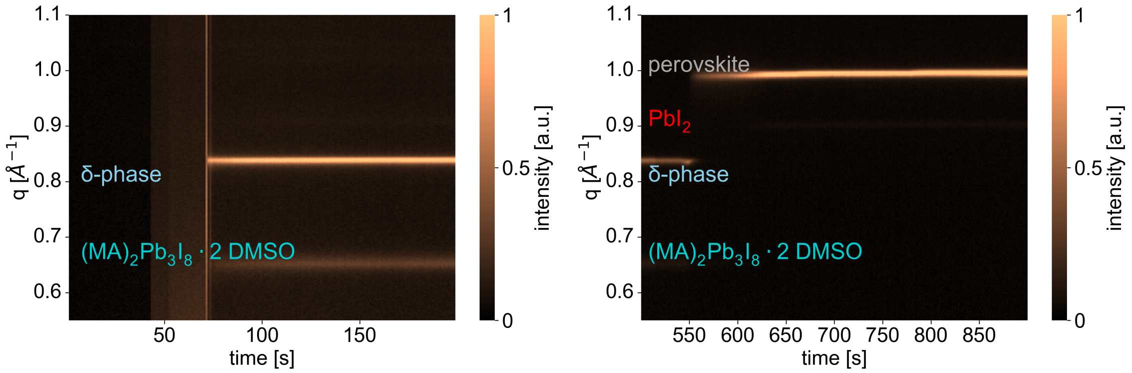

Figure S51: Method: antisolvent (IPA); Composition: $MA_{0.5}FA_{0.5}PbI_3$. Pseudo-2D XRD data in a selected q-range showing the signals of interest, extracted from the in-situ GIWAXS data during the spin-coating (left) and annealing (right) to follow the crystallization of the sample. The phases corresponding to the peaks visible, are stated on the left side of the figures.

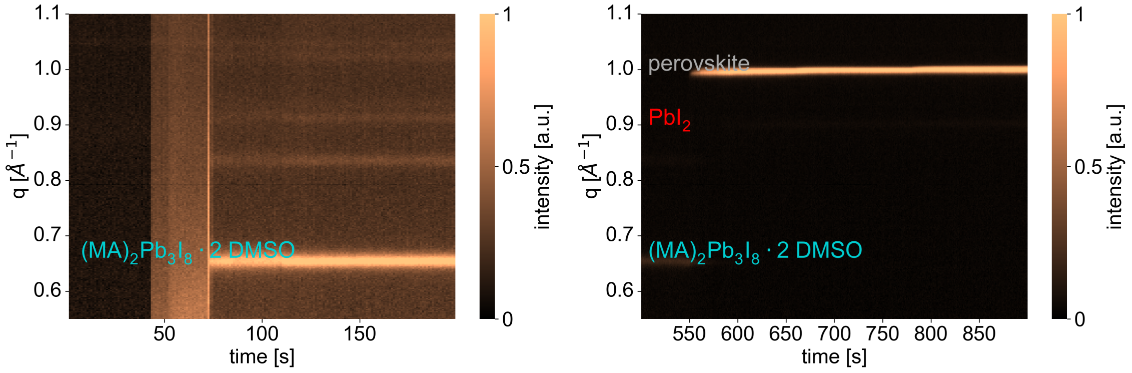

Figure S52: Method: antisolvent (IPA); Composition: $MA_{0.83}FA_{0.17}PbI_3$. Pseudo-2D XRD data in a selected q-range showing the signals of interest, extracted from the in-situ GIWAXS data during the spin-coating (left) and annealing (right) to follow the crystallization of the sample. The phases corresponding to the peaks visible, are stated on the left side of the figures.

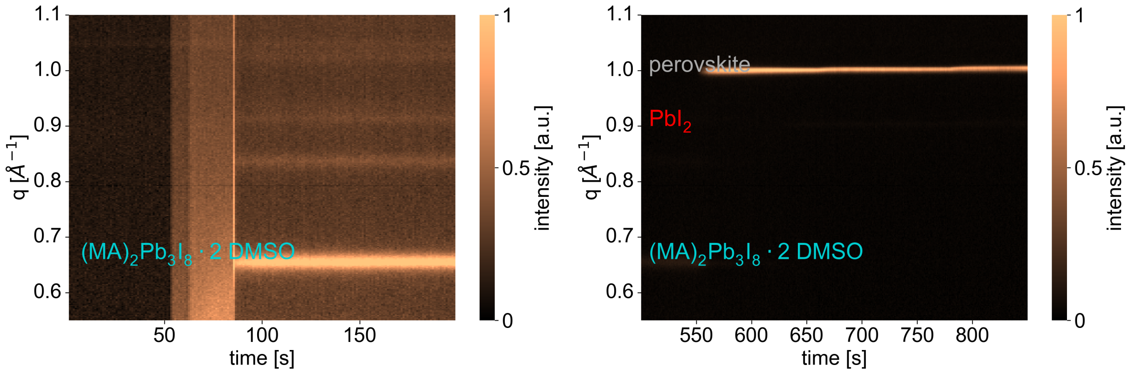

Figure S53: Method: antisolvent (IPA); Composition: $MAPbI_3$. Pseudo-2D XRD data in a selected q-range showing the signals of interest, extracted from the in-situ GIWAXS data during the spin-coating (left) and annealing (right) to follow the crystallization of the sample. The phases corresponding to the peaks visible, are stated on the left side of the figures.

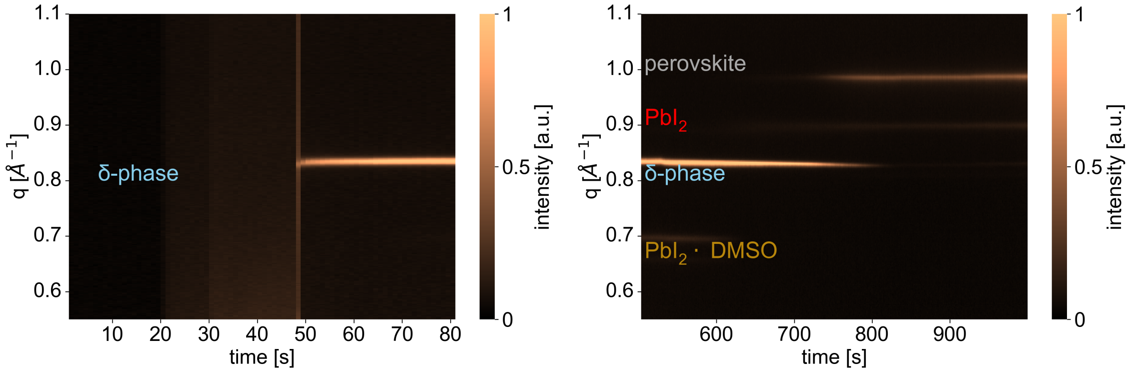

Figure S54: Method: antisolvent (EtOH); Composition: $FAPbI_3$. Pseudo-2D XRD data in a selected q-range showing the signals of interest, extracted from the in-situ GIWAXS data during the spin-coating (left) and annealing (right) to follow the crystallization of the sample. The phases corresponding to the peaks visible, are stated on the left side of the figures.

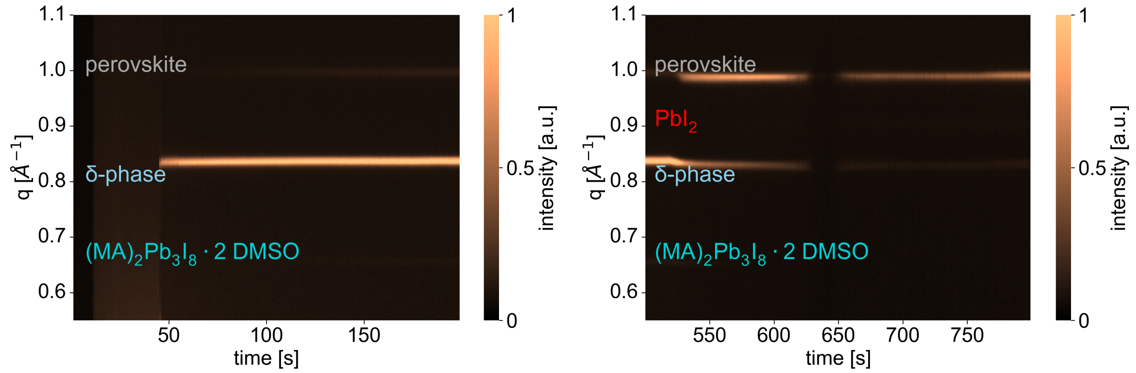

Figure S55: Method: antisolvent (EtOH); Composition: $(MA_{0.17}FA_{0.83})_{0.95}Cs_{0.05}PbI_3$. Pseudo-2D XRD in a selected q-range showing the signals of interest, data extracted from the in-situ GIWAXS data during the spin-coating (left) and annealing (right) to follow the crystallization of the sample. The phases corresponding to the peaks visible, are stated on the left side of the figures.

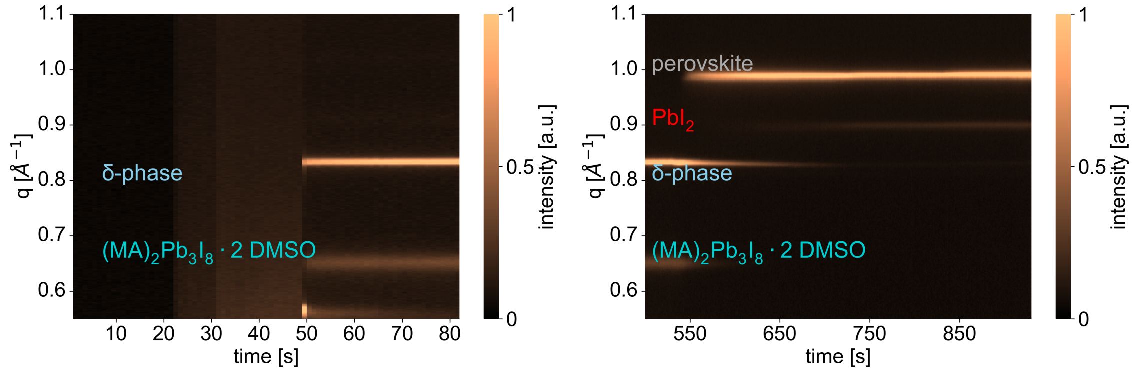

Figure S56: Method: antisolvent (EtOH); Composition: $MA_{0.5}FA_{0.5}PbI_3$. Pseudo-2D XRD data in a selected q-range showing the signals of interest, extracted from the in-situ GIWAXS data during the spin-coating (left) and annealing (right) to follow the crystallization of the sample. The phases corresponding to the peaks visible, are stated on the left side of the figures.

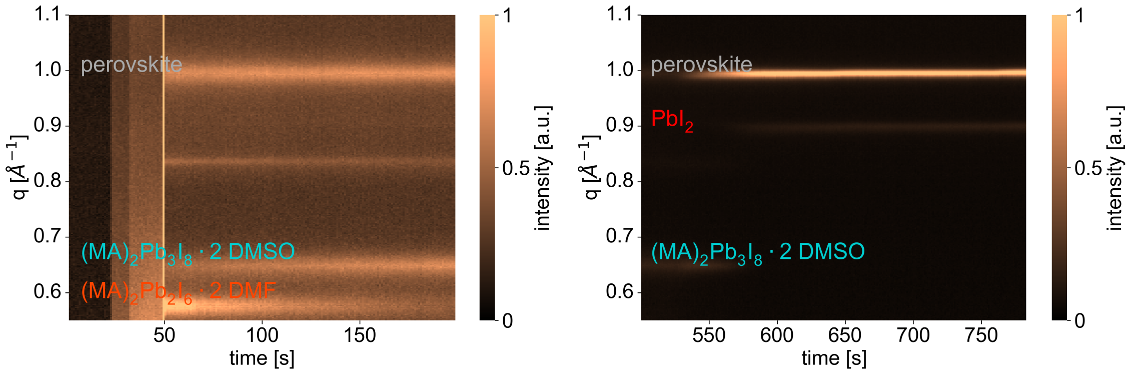

Figure S57: Method: antisolvent (EtOH); Composition: $MA_{0.83}FA_{0.17}PbI_3$. Pseudo-2D XRD data in a selected q-range showing the signals of interest, extracted from the in-situ GIWAXS data during the spin-coating (left) and annealing (right) to follow the crystallization of the sample. The phases corresponding to the peaks visible, are stated on the left side of the figures.

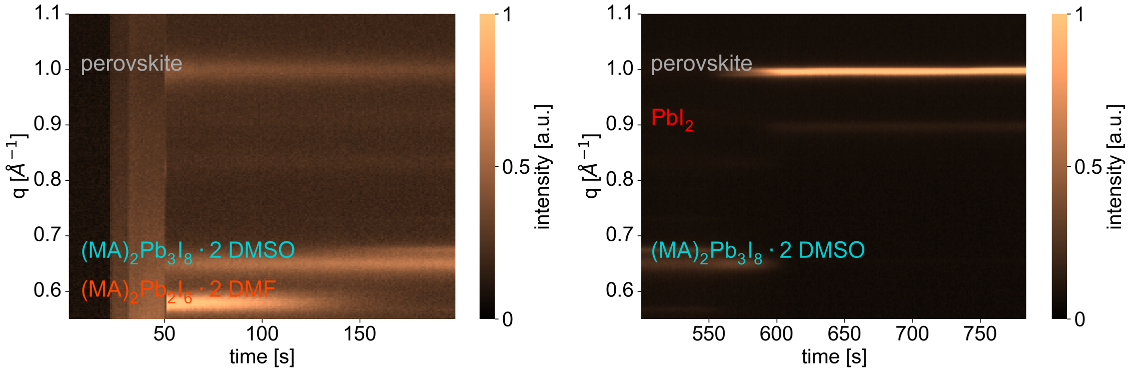

Figure S58: Method: antisolvent (EtOH); Composition: $MAPbI_3$. Pseudo-2D XRD data in a selected q-range showing the signals of interest, extracted from the in-situ GIWAXS data during the spin-coating (left) and annealing (right) to follow the crystallization of the sample. The phases corresponding to the peaks visible, are stated on the left side of the figures.

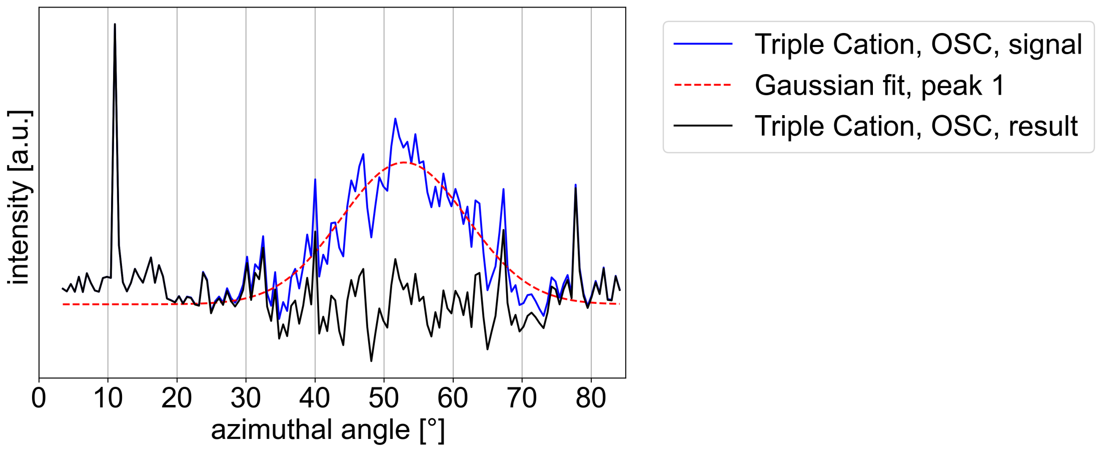

Figure S59: Method: OSC; Composition: $(MA_{0.17}FA_{0.83})_{0.95}Cs_{0.05}PbI_3$. Background-corrected azimuthal profile of the 100 peak of the perovskite phase (blue line), Gaussian fit of the peak in the profile due to a preferred orientation (red dashed line) and the orientation-corrected resulting azimuthal profile (black line).

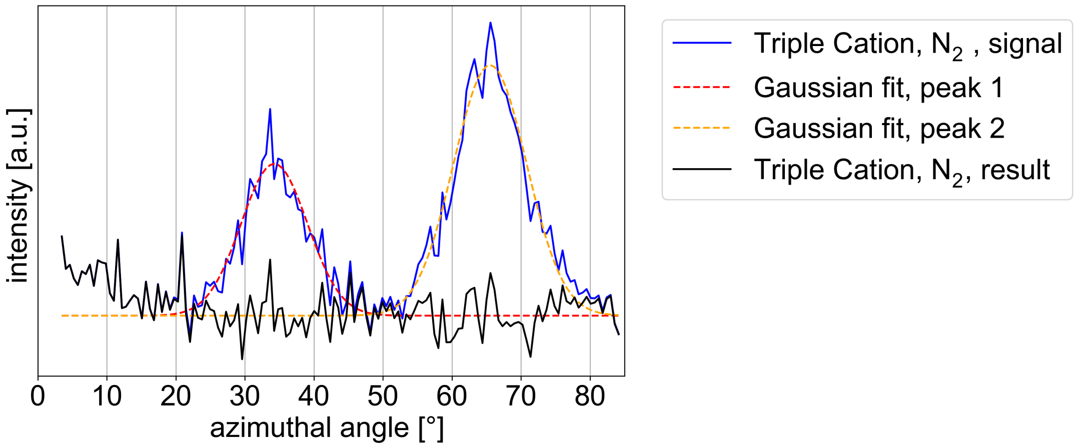

Figure S60: Method: gas-quenching; Composition: $(MA_{0.17}FA_{0.83})_{0.95}Cs_{0.05}PbI_3$. Background-corrected azimuthal profile of the 100 peak of the perovskite phase (blue line), Gaussian fits of peaks in the profile due to a preferred orientation (red and orange dashed lines) and the orientation-corrected resulting azimuthal profile (black line).

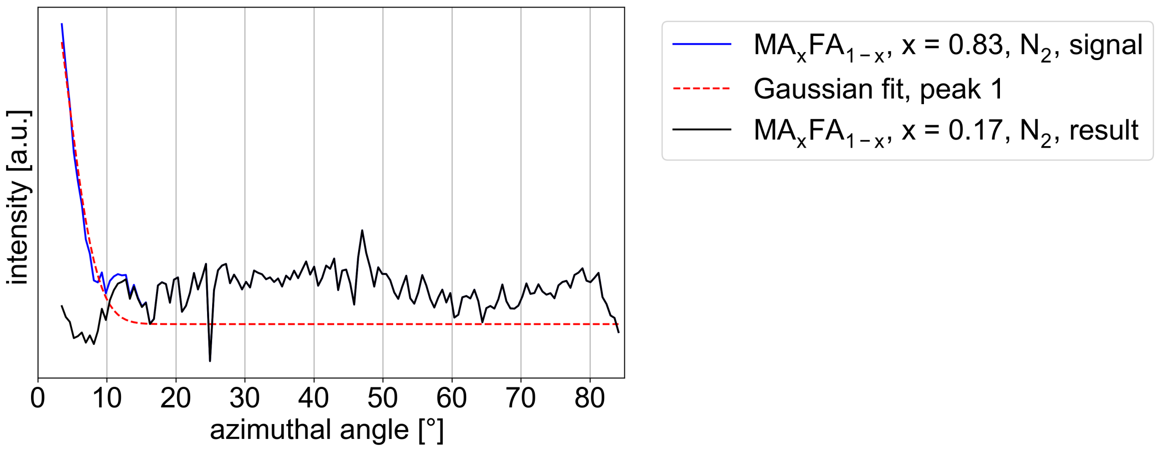

Figure S61: Method: gas-quenching; Composition: $MA_{0.83}FA_{0.17}PbI_3$. Background-corrected azimuthal profile of the 100 peak of the perovskite phase (blue line), Gaussian fit of the peak in the profile due to a preferred orientation (red dashed line) and the orientation-corrected resulting azimuthal profile (black line).

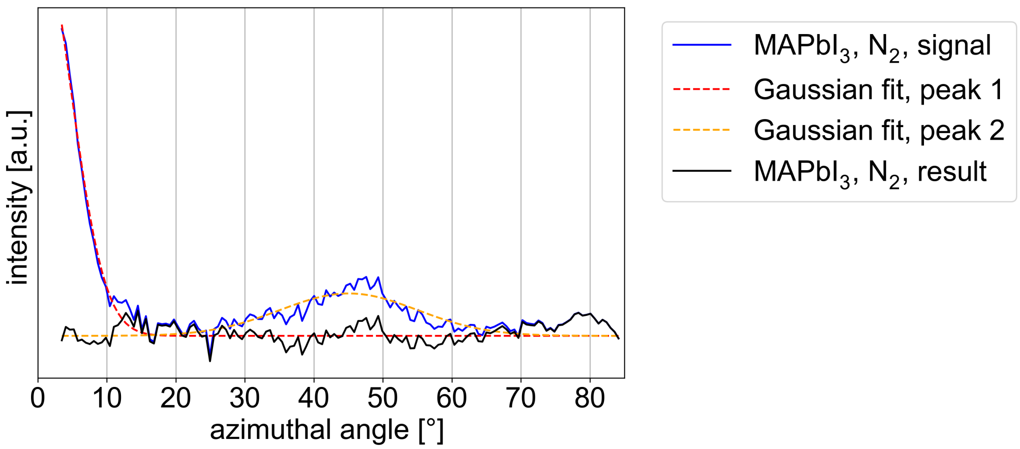

Figure S62: Method: gas-quenching; Composition: $MAPbI_3$. Background-corrected azimuthal profile of the 100 peak of the perovskite phase (blue line), Gaussian fits of peaks in the profile due to a preferred orientation (red and orange dashed lines) and the orientation-corrected resulting azimuthal profile (black line).

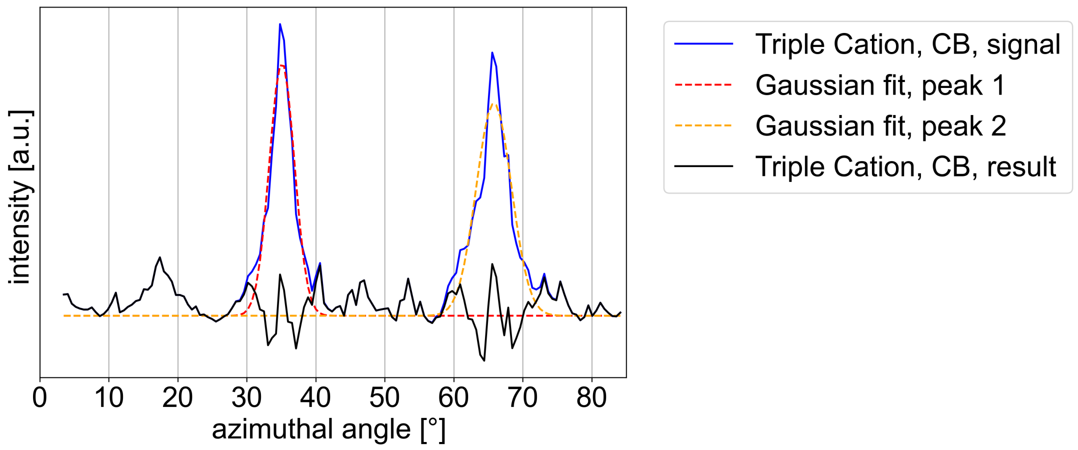

Figure S63: Method: antisolvent (CB); Composition: $(MA_{0.17}FA_{0.83})_{0.95}Cs_{0.05}PbI_3$. Background-corrected azimuthal profile of the 100 peak of the perovskite phase (blue line), Gaussian fits of peaks in the profile due to a preferred orientation (red and orange dashed lines) and the orientation-corrected resulting azimuthal profile (black line).

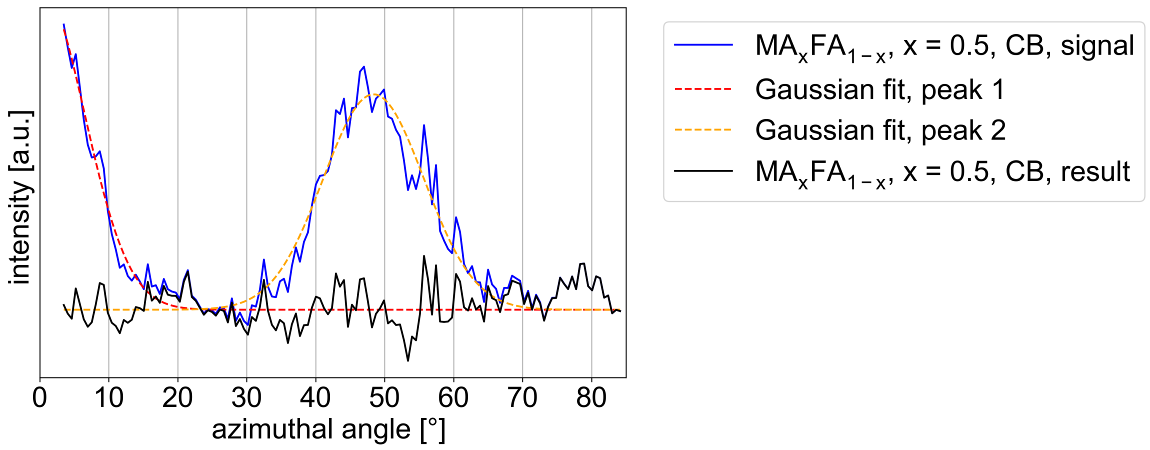

Figure S64: Method: antisolvent (CB); Composition: $MA_{0.5}FA_{0.5}PbI_3$. Background-corrected azimuthal profile of the 100 peak of the perovskite phase (blue line), Gaussian fits of peaks in the profile due to a preferred orientation (red and orange dashed lines) and the orientation-corrected resulting azimuthal profile (black line).

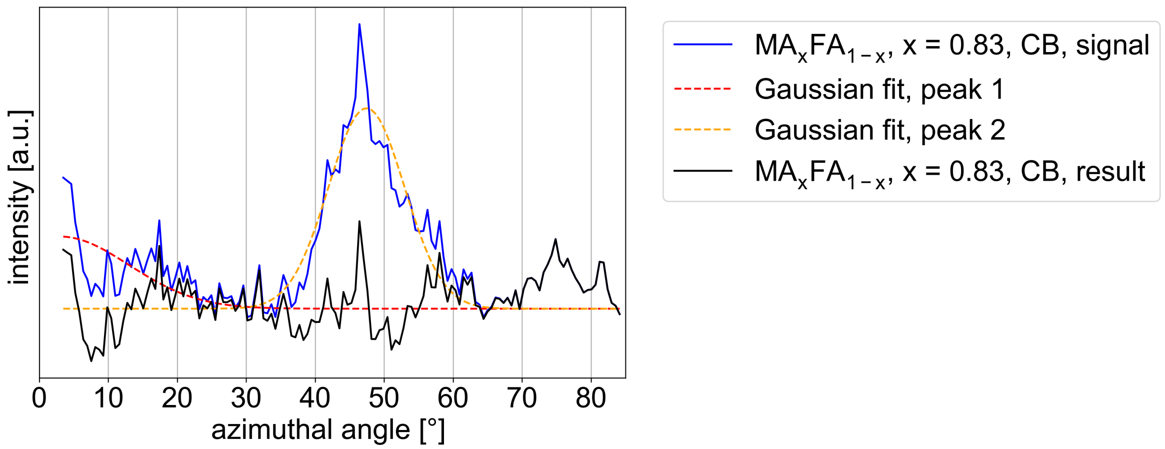

Figure S65: Method: antisolvent (CB); Composition: $MA_{0.83}FA_{0.17}PbI_3$. Background-corrected azimuthal profile of the 100 peak of the perovskite phase (blue line), Gaussian fits of peaks in the profile due to a preferred orientation (red and orange dashed lines) and the orientation-corrected resulting azimuthal profile (black line).

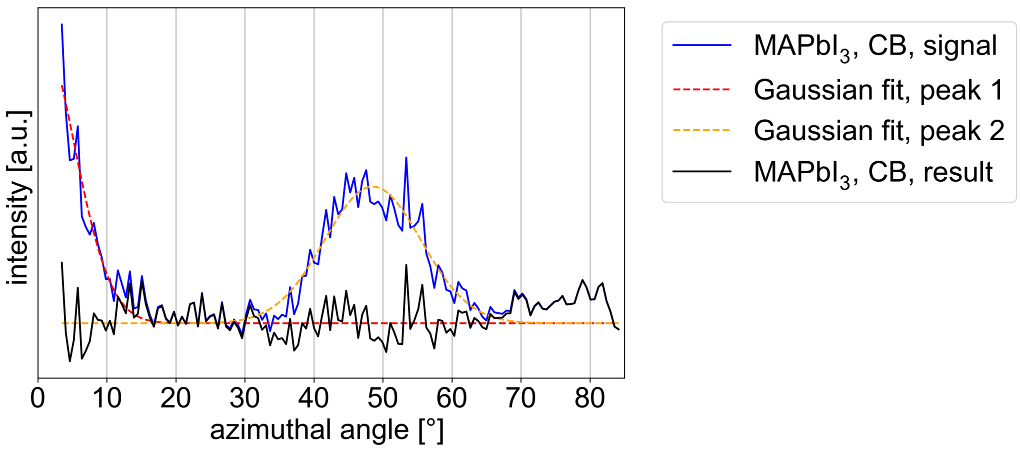

Figure S66: Method: antisolvent (CB); Composition: $MAPbI_3$. Background-corrected azimuthal profile of the 100 peak of the perovskite phase (blue line), Gaussian fits of peaks in the profile due to a preferred orientation (red and orange dashed lines) and the orientation-corrected resulting azimuthal profile (black line).

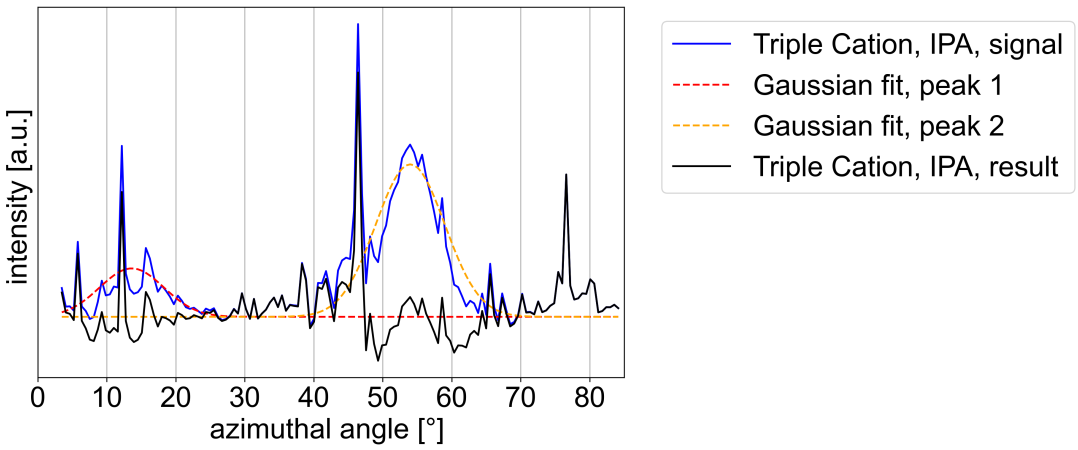

Figure S67: Method: antisolvent (IPA); Composition: $(MA_{0.17}FA_{0.83})_{0.95}Cs_{0.05}PbI_3$. Background-corrected azimuthal profile of the 100 peak of the perovskite phase (blue line), Gaussian fits of peaks in the profile due to a preferred orientation (red and orange dashed lines) and the orientation-corrected resulting azimuthal profile (black line).

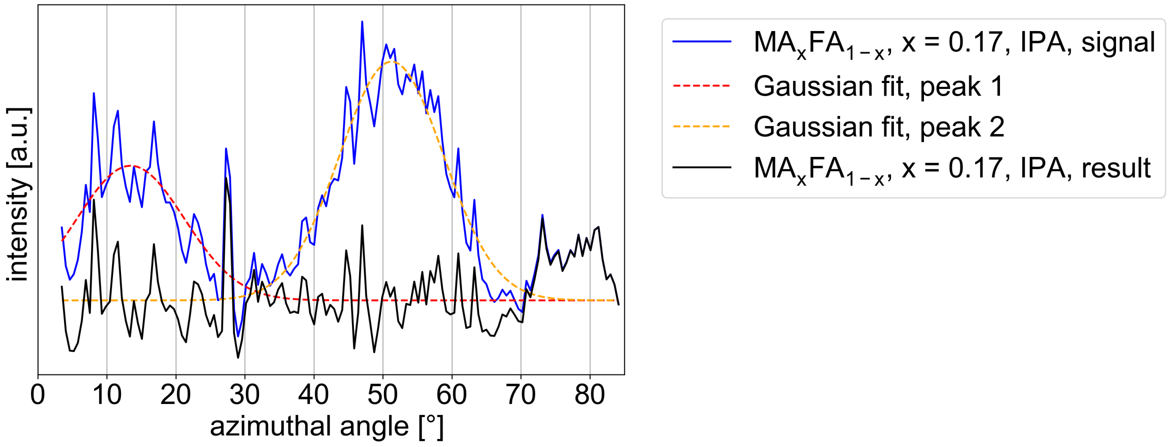

Figure S68: Method: antisolvent (IPA); Composition: $MA_{0.17}FA_{0.83}PbI_3$. Background-corrected azimuthal profile of the 100 peak of the perovskite phase (blue line), Gaussian fits of peaks in the profile due to a preferred orientation (red and orange dashed lines) and the orientation-corrected resulting azimuthal profile (black line).

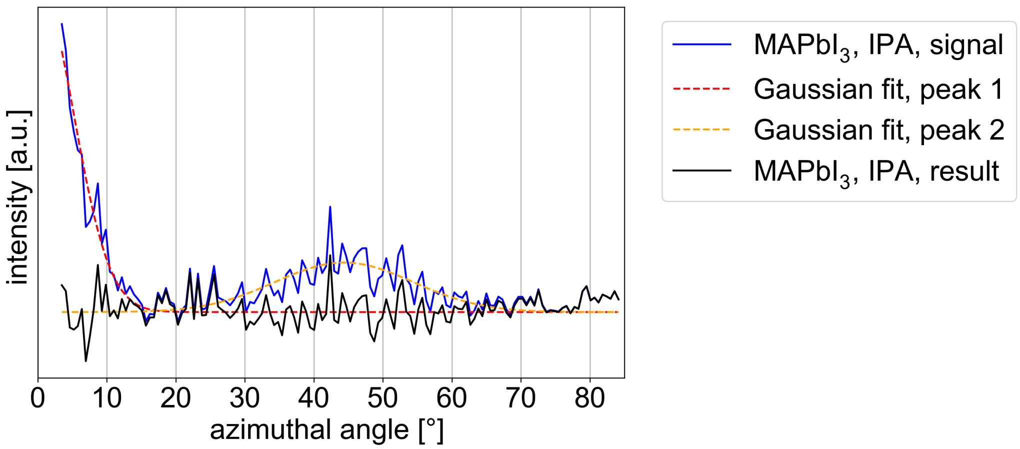

Figure S69: Method: antisolvent (IPA); Composition: $MAPbI_3$. Background-corrected azimuthal profile of the 100 peak of the perovskite phase (blue line), Gaussian fits of peaks in the profile due to a preferred orientation (red and orange dashed lines) and the orientation-corrected resulting azimuthal profile (black line).

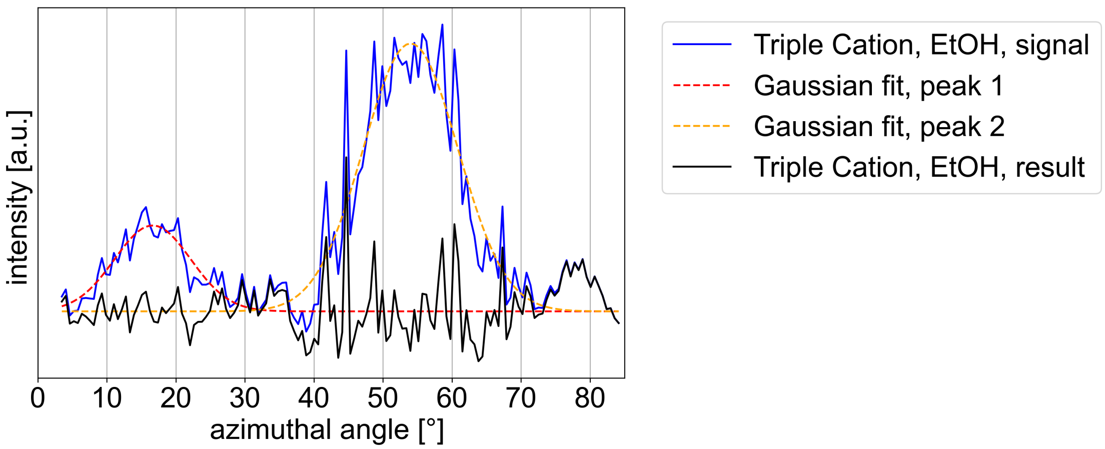

Figure S70: Method: antisolvent (EtOH); Composition: $(MA_{0.17}FA_{0.83})_{0.95}Cs_{0.05}PbI_3$. Background-corrected azimuthal profile of the 100 peak of the perovskite phase (blue line), Gaussian fits of peaks in the profile due to a preferred orientation (red and orange dashed lines) and the orientation-corrected resulting azimuthal profile (black line).

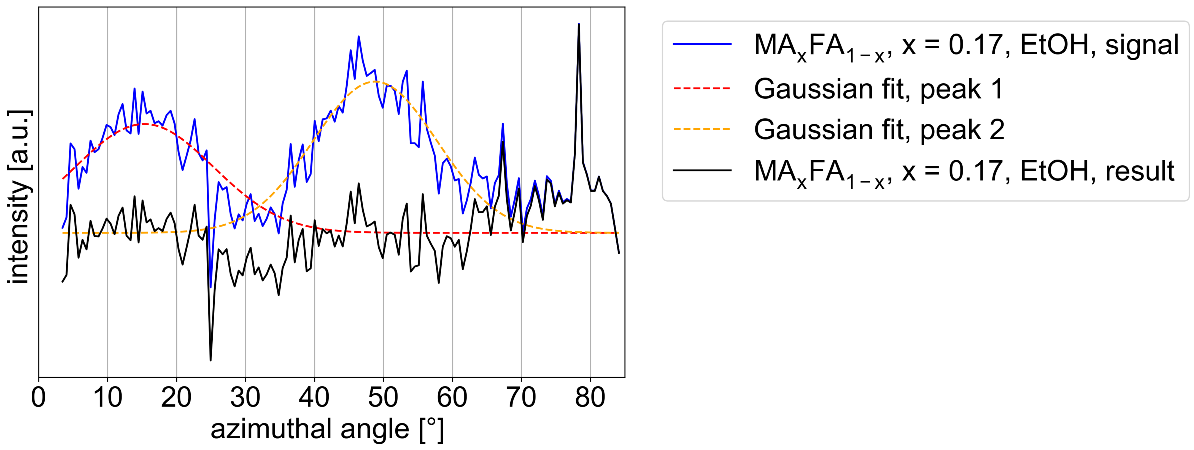

Figure S71: Method: antisolvent (EtOH); Composition: $MA_{0.17}FA_{0.83}PbI_3$. Background-corrected azimuthal profile of the 100 peak of the perovskite phase (blue line), Gaussian fits of peaks in the profile due to a preferred orientation (red and orange dashed lines) and the orientation-corrected resulting azimuthal profile (black line).

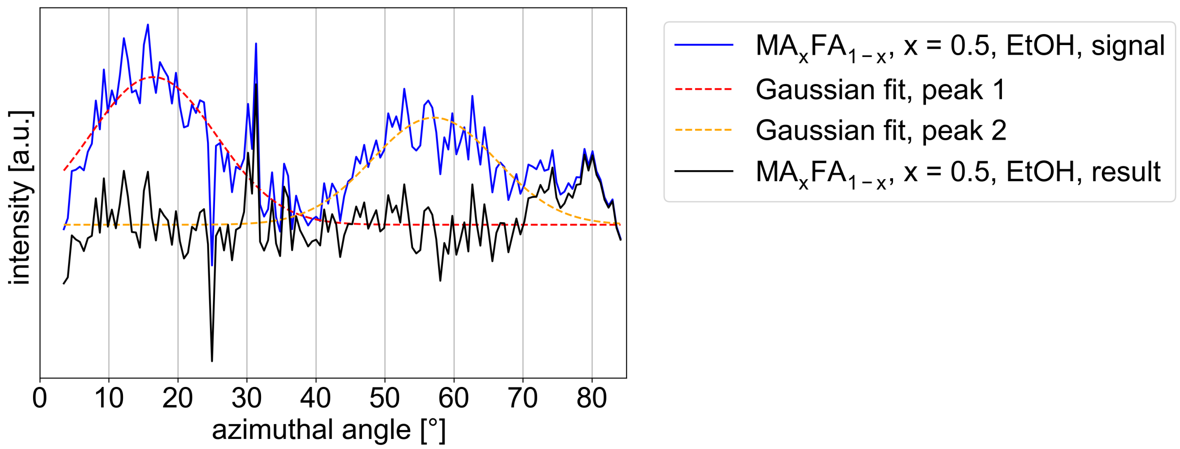

Figure S72: Method: antisolvent (EtOH); Composition: $MA_{0.5}FA_{0.5}PbI_3$. Background-corrected azimuthal profile of the 100 peak of the perovskite phase (blue line), Gaussian fits of peaks in the profile due to a preferred orientation (red and orange dashed lines) and the orientation-corrected resulting azimuthal profile (black line).

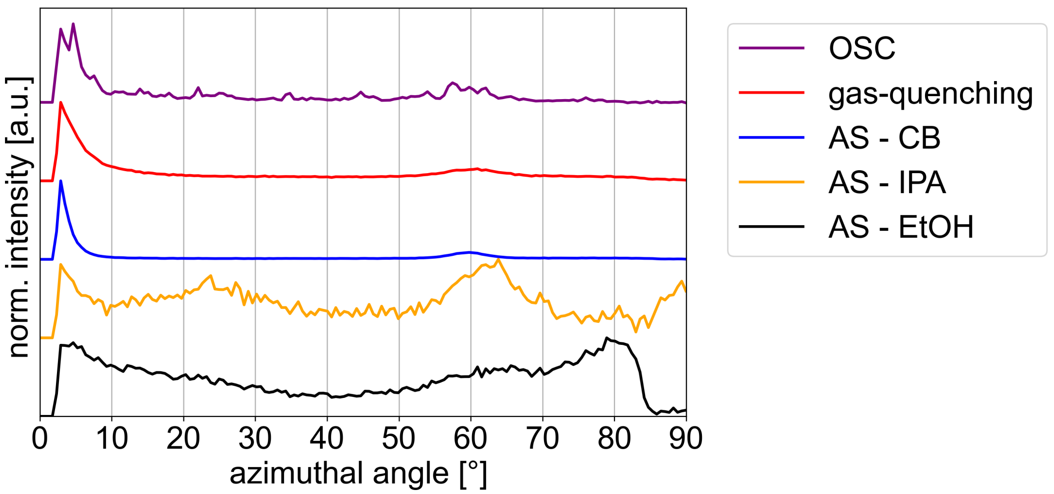

Figure S73: Normalized azimuthal profile of the 100 diffraction signal of the δ-phase ($|q| = 0.83$ Å^{-1}) for the $FAPbI_3$ composition for all fabrication methods. Profiles extracted from GIWAXS data taken during the annealing step of the film preparation with an angle of incidence of $\alpha = 0.5°$.

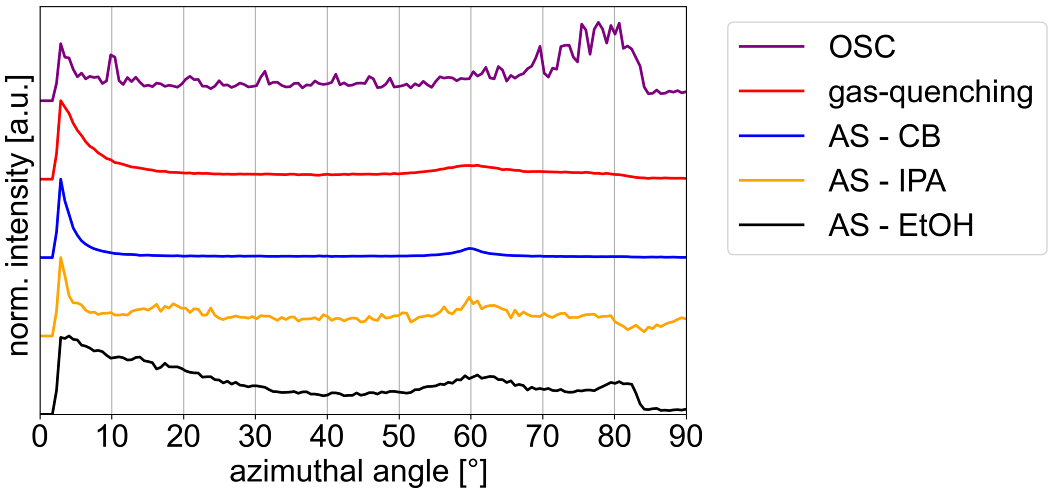

Figure S74: Normalized azimuthal profile of the 100 diffraction signal of the δ-phase ($|q| = 0.83$ Å^{-1}) for the $(MA_{0.17}FA_{0.83})_{0.95}Cs_{0.05}PbI_3$ composition for all fabrication methods. Profiles extracted from GIWAXS data taken during the annealing step of the film preparation with an angle of incidence of $\alpha = 0.5°$.

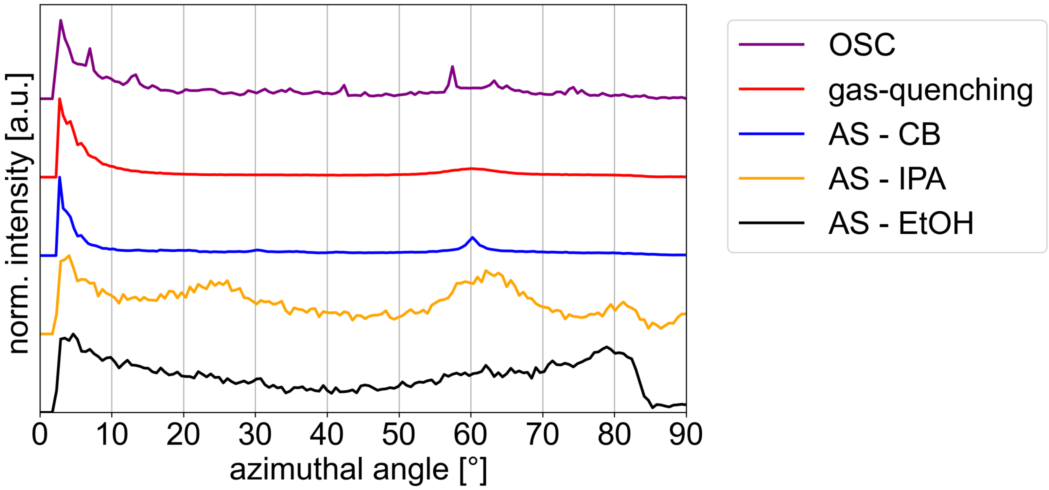

Figure S75: Normalized azimuthal profile of the 100 diffraction signal of the δ-phase ($|q| = 0.83$ Å^{-1}) for the $MA_{0.17}FA_{0.83}PbI_3$ composition for all fabrication methods. Profiles extracted from GIWAXS data taken during the annealing step of the film preparation with an angle of incidence of $\alpha = 0.5°$.

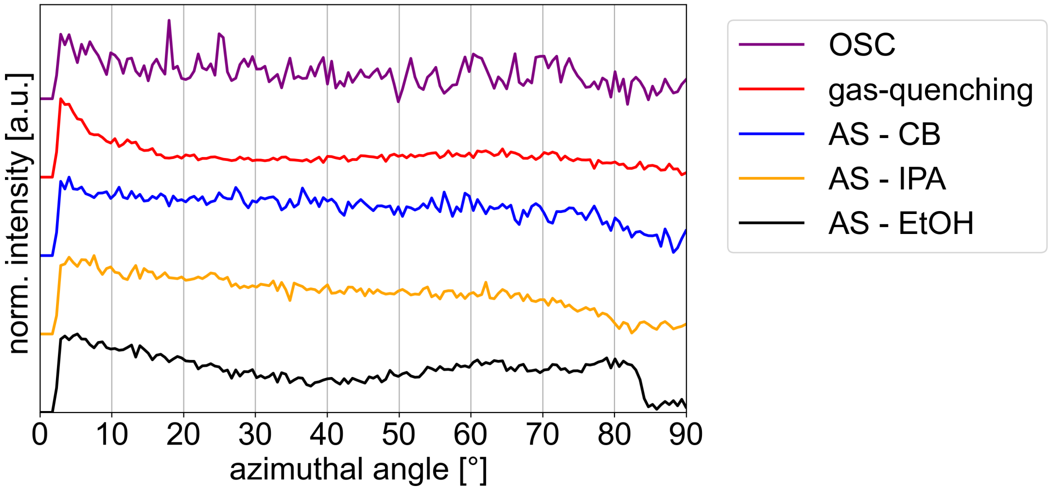

Figure S76: Normalized azimuthal profile of the 100 diffraction signal of the δ-phase ($|q| = 0.83$ Å^{-1}) for the $MA_{0.5}FA_{0.5}PbI_3$ composition for all fabrication methods. Profiles extracted from GIWAXS data taken during the annealing step of the film preparation with an angle of incidence of $\alpha = 0.5°$.

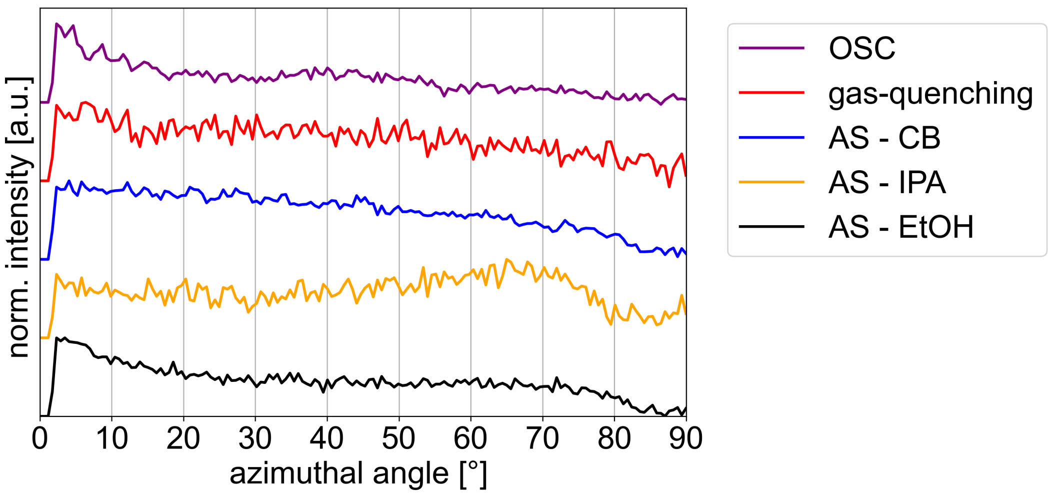

Figure S77: Normalized azimuthal profile of the 022 diffraction signal of the $(MA)_2Pb_3I_8 \cdot 2$ DMSO solvent complex ($|q| = 0.65$ Å^{-1}) for the $MA_{0.5}FA_{0.5}PbI_3$ composition for all fabrication methods. Profiles extracted from GIWAXS data taken during the annealing step of the film preparation with an angle of incidence of $\alpha = 0.5°$.

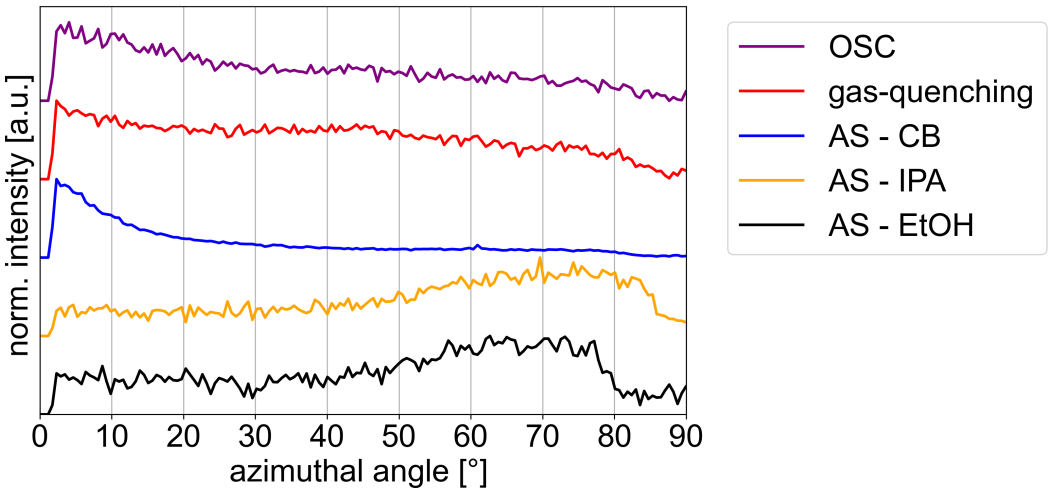

Figure S78: Normalized azimuthal profile of the 022 diffraction signal of the $(MA)_2Pb_3I_8 \cdot 2$ DMSO solvent complex ($|q| = 0.65$ Å^{-1}) for the $MA_{0.83}FA_{0.17}PbI_3$ composition for all fabrication methods. Profiles extracted from GIWAXS data taken during the annealing step of the film preparation with an angle of incidence of $\alpha = 0.5°$.

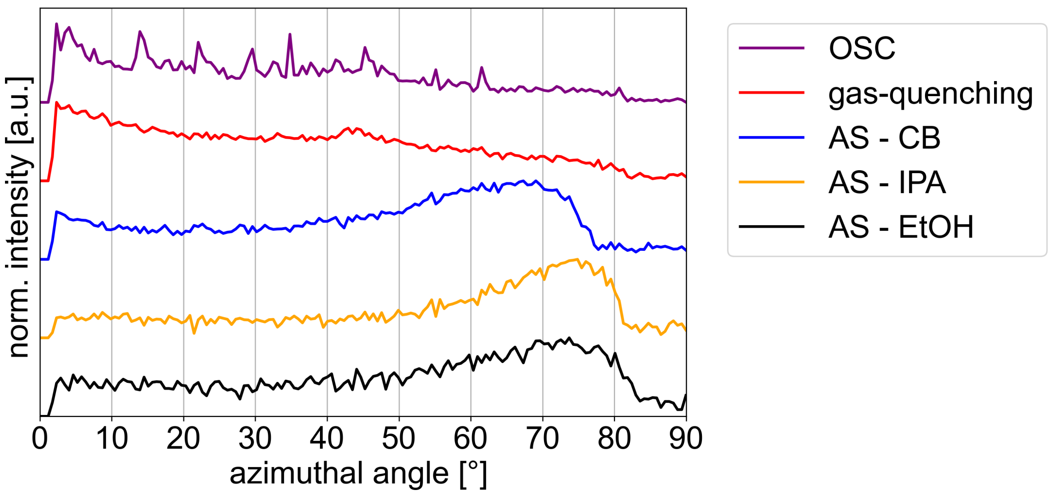

Figure S79: Normalized azimuthal profile of the 022 diffraction signal of the $(MA)_2Pb_3I_8 \cdot 2$ DMSO solvent complex ($|q| = 0.65$ Å^{-1}) for the $MAPbI_3$ composition for all fabrication methods. Profiles extracted from GIWAXS data taken during the annealing step of the film preparation with an angle of incidence of $\alpha = 0.5°$.
